# Is iron unique in promoting electrical conductivity in MOFs?†
†Electronic supplementary information (ESI) available: Experimental details, PXRD patterns, IR spectra, table of electrical conductivity, table of activation energies, current density *vs.* electrical field strength curves, current–voltage curves at various temperatures, temperature dependence of electrical conductivity, ^57^Fe Mössbauer spectra, magnetic susceptibility plots, BET surface area analysis, table of various properties of divalent metal ions, and calculation details. See DOI: 10.1039/c7sc00647k



**DOI:** 10.1039/c7sc00647k

**Published:** 2017-04-20

**Authors:** Lei Sun, Christopher H. Hendon, Sarah S. Park, Yuri Tulchinsky, Ruomeng Wan, Fang Wang, Aron Walsh, Mircea Dincă

**Affiliations:** a Department of Chemistry , Massachusetts Institute of Technology , Cambridge , MA 02139 , USA . Email: mdinca@mit.edu; b Department of Materials , Imperial College London , London SW7 2AZ , UK; c Department of Materials Science and Engineering , Yonsei University , Seoul 03722 , South Korea

## Abstract

Identifying the metal ions that optimize charge transport and charge density in metal–organic frameworks is critical for systematic improvements in the electrical conductivity in these materials. In this work, we measure the electrical conductivity and activation energy for twenty different MOFs pertaining to four distinct structural families: M_2_(DOBDC)(DMF)_2_ (M = Mg^2+^, Mn^2+^, Fe^2+^, Co^2+^, Ni^2+^, Cu^2+^, Zn^2+^); H_4_DOBDC = 2,5-dihydroxybenzene-1,4-dicarboxylic acid; DMF = *N*,*N*-dimethylformamide), M_2_(DSBDC)(DMF)_2_ (M = Mn^2+^, Fe^2+^; H_4_DSBDC = 2,5-disulfhydrylbenzene-1,4-dicarboxylic acid), M_2_Cl_2_(BTDD)(DMF)_2_ (M = Mn^2+^, Fe^2+^, Co^2+^, Ni^2+^; H_2_BTDD = bis(1*H*-1,2,3-triazolo[4,5-b],[4′,5′-*i*]dibenzo[1,4]dioxin), and M(1,2,3-triazolate)_2_ (M = Mg^2+^, Mn^2+^, Fe^2+^, Co^2+^, Cu^2+^, Zn^2+^, Cd^2+^). This comprehensive study allows us to single-out iron as the metal ion that leads to the best electrical properties. The iron-based MOFs exhibit at least five orders of magnitude higher electrical conductivity and significantly smaller charge activation energies across all different MOF families studied here and stand out materials made from all other metal ions considered here. We attribute the unique electrical properties of iron-based MOFs to the high-energy valence electrons of Fe^2+^ and the Fe^3+/2+^ mixed valency. These results reveal that incorporating Fe^2+^ in the charge transport pathways of MOFs and introducing mixed valency are valuable strategies for improving electrical conductivity in this important class of porous materials.

## Abstract 2

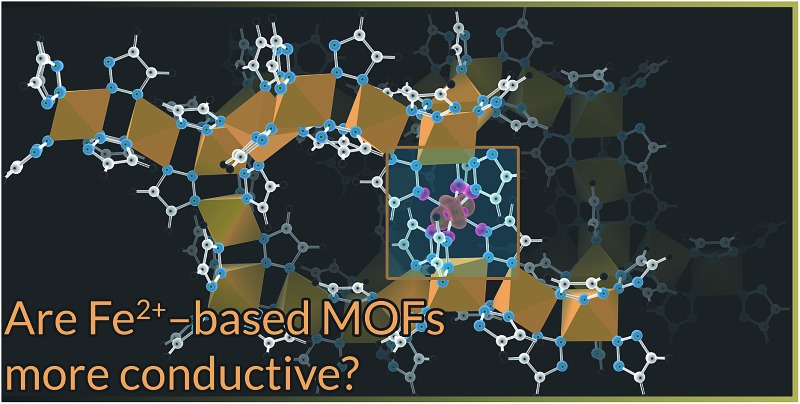
Identifying the metal ions that optimize charge transport and charge density in metal–organic frameworks is critical for systematic improvements in the electrical conductivity in these materials.

## Introduction

Metal–organic frameworks (MOFs) that exhibit both high surface area and electrical conductivity are emerging as a new class of materials whose applications reach beyond those typical of porous solids.^1^ Reports of electrically conductive MOFs in the last few years have addressed both the fundamentals: the nature of the charge carriers and the mechanism of transport,^2–6^ and the applications: supercapacitors,^7^ electrocatalysis,^8,9^ chemiresistive sensing,^10,11^ and thermoelectrics^12^ among others. Certain design principles have emerged from these studies, focused for instance on targeting either band-like or hopping conductors,^13^ yet some of the most basic questions governing electrical conduction in MOFs are still poorly understood. Most obvious among these is the influence of the metal ions on either the band structure of the underlying material or the charge density.

In our previous work we have shown that in two isostructural MOFs made from Mn and Fe, the latter leads to considerably improved electrical conductivity by up to six orders of magnitude.^5^ Additionally, the Fe analogs of M(1,2,3-triazolate)_2_ (M = Mg^2+^, Mn^2+^, Fe^2+^, Co^2+^, Cu^2+^, Zn^2+^, Cd^2+^)^14,15^ and M(TCNQ) (4,4′-bpy) (M = Mn^2+^, Fe^2+^, Co^2+^, Zn^2+^, Cd^2+^; TCNQ = 7,7,8,8-tetracyanoquinodimethane; 4,4′-bpy = 4,4′-bipyridyl)^16^ were reported as being electrically conductive, although the electrical conductivity in the other analogs was not reported. These isolated reports led us to believe that Fe may play an important and unique role in promoting electrical conductivity in MOFs. Here, we compare four structurally distinct classes of MOFs, totalling twenty different materials made from eight different metal ions (M = Mg^2+^, Mn^2+^, Fe^2+^, Co^2+^, Ni^2+^, Cu^2+^, Zn^2+^, Cd^2+^) and show that Fe does indeed enable high electrical conductivity in Fe-containing frameworks.

**Fig. 1:**
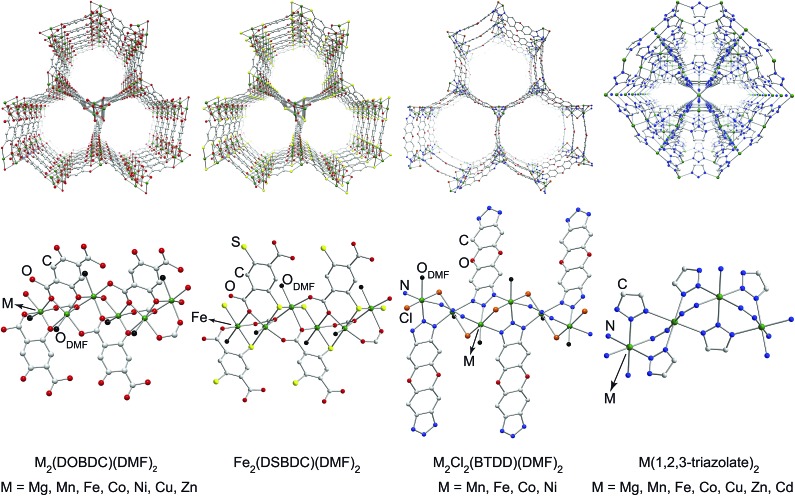
Portions of crystal structures of four families of MOFs emphasizing pores (top) and coordination environment of metal ions (bottom). H atoms and part of DMF molecules have been omitted for clarity. The structure of Mn_2_(DSBDC)(DMF)_2_ is shown in Fig. S2 in the ESI.†

To ascertain the influence of the metal cation on electrical conductivity systematically, we targeted MOFs that feature a broad array of chemical connectivity and composition. Four families of materials that provide this breadth are M_2_(DOBDC)(DMF)_2_ (M = Mg^2+^, Mn^2+^, Fe^2+^, Co^2+^, Ni^2+^, Cu^2+^, Zn^2+^),^17–24^ M_2_(DSBDC)(DMF)_2_ (M = Mn^2+^, Fe^2+^),^4,5^ M_2_Cl_2_(BTDD)(DMF)_2_ (M = Mn^2+^, Fe^2+^, Co^2+^, Ni^2+^),^25^ and M(1,2,3-triazolate)_2_ (M = Mg^2+^, Mn^2+^, Fe^2+^, Co^2+^, Cu^2+^, Zn^2+^, Cd^2+^).^14,15^ The first three families of MOFs display honeycomb structures with 1D tubular pores, whereas the M(1,2,3-triazolate)_2_ materials exhibit cubic structures with three-dimensional pore networks.‡
‡In M_2_(DOBDC)(DMF)_2_, M_2_Cl_2_(BTDD)(DMF)_2_, and M(1,2,3-triazolate)_2_, the MOFs are isostructural in each family, with the only difference among them being metal ions. Although Mn_2_(DSBDC)(DMF)_2_ and Fe_2_(DSBDC)(DMF)_2_ bear the same topology, the coordination environments of Mn and Fe differ. Whereas the former exhibits two crystallographically distinct Mn sites, the latter has only one crystallographically distinct Fe site (Fig. 1 and S2†). The connectivity in the (–Mn–S–)_∞_ chains is otherwise conserved, such that the subtle difference in the local coordination environment should not affect the electrical properties significantly. The metal ions in all these MOFs are formally divalent and octahedrally coordinated (Fig. 1 and S2†).

## Experimental results

All Mn^2+^-, Fe^2+^-, and Co^2+^-based materials were synthesized under air-free conditions. Literature procedures were available for all materials studied here, with the exception of Fe_2_Cl_2_(BTDD)(DMF)_2_ (MIT-20-Fe), which was synthesized by adapting a strategy similar to the preparation of the Mn, Co, and Ni analogs.^25^ Its structure was assigned on the basis of powder X-ray diffraction (PXRD) analysis, which revealed a pattern that matches those of the other analogs (Fig. S3c†). To ensure consistency, all MOFs were soaked successively in dry and degassed DMF and dichloromethane (DCM) under air-free conditions, and evacuated at 100 °C under vacuum for 2 h. The evacuated materials were kept in a N_2_-filled glovebox. PXRD and elemental analyses confirmed that all materials retain their structural and compositional integrity as well as phase purity during these manipulations (Fig. S3†). As reported previously, Fe_2_(DSBDC)(DMF)_2_ undergoes a spontaneous structural distortion (*i.e.* a “breathing” deformation) but maintains its connectivity.^5^ Infrared (IR) spectroscopy revealed vibrational modes at approximately 1650 cm^–1^ for M_2_(DOBDC)(DMF)_2_, M_2_(DSBDC)(DMF)_2_, and M_2_Cl_2_(BTDD)(DMF)_2_, confirming that bound DMF completes the octahedral coordination environment of the metal ions in these materials (Fig. S4†).

**Fig. 2:**
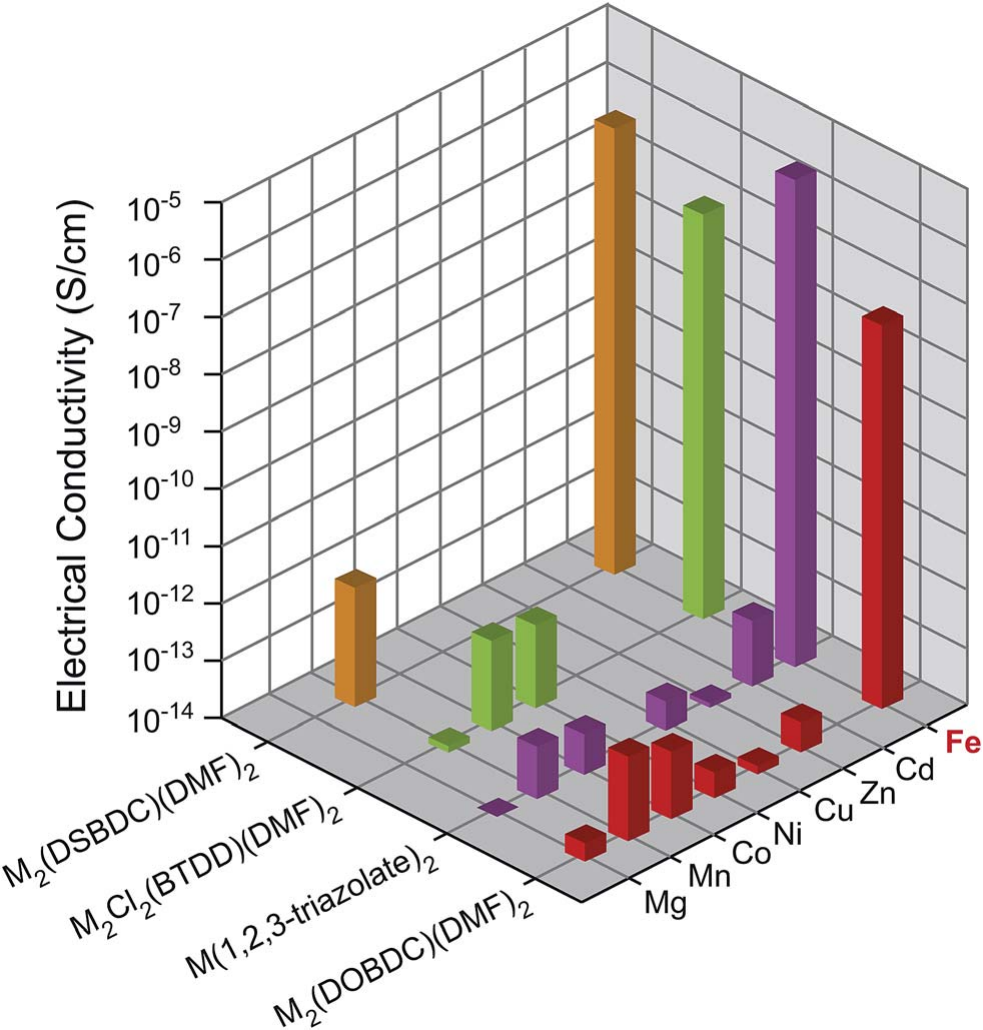
Electrical conductivity in M_2_(DOBDC)(DMF)_2_, M_2_(DSBDC)(DMF)_2_, M_2_Cl_2_(BTDD)(DMF)_2_, and M(1,2,3-triazolate)_2_ measured at 300 K, in N_2_ atmosphere, and in the dark.

Because some of the MOF crystallites were too small for single crystal studies, electrical properties were measured on pressed pellets in all cases using the standard two-contact probe method^26,27^ at 300 K, under a N_2_ atmosphere, and in the dark. PXRD analysis of the pressed pellets revealed patterns that match those of the original materials (Fig. S5†). Plots of the observed current density (*J*) *versus* electric field strength (*E*) for all MOFs are shown in Fig. S6,† and the electrical conductivity values are summarized in Fig. 2 and Table S1.† The Fe-based MOFs exhibit electrical conductivity on the order of 10^–8^–10^–6^ S cm^–1^, whereas the observed electrical conductivity in all other MOFs is six orders of magnitude lower, on the order of 10^–14^–10^–12^ S cm^–1^.

**Fig. 3:**
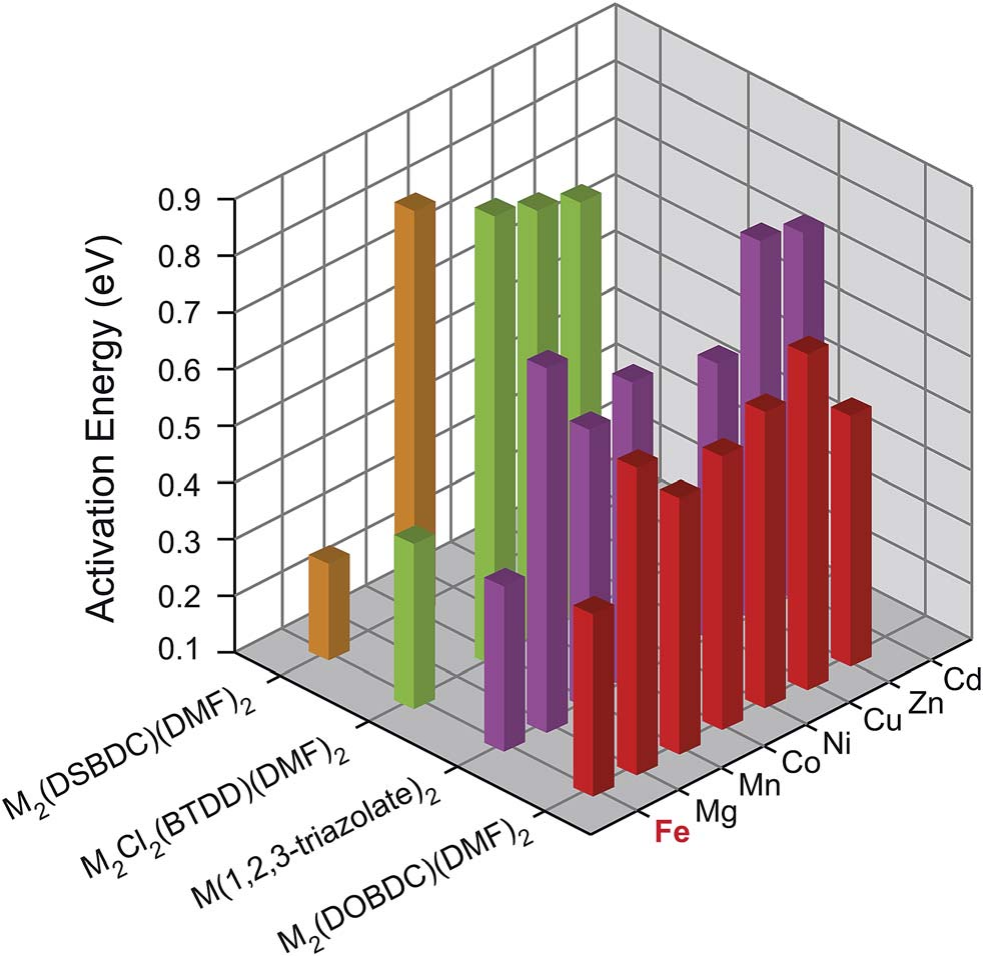
Activation energies of M_2_(DOBDC)(DMF)_2_, M_2_(DSBDC)(DMF)_2_, M_2_Cl_2_(BTDD)(DMF)_2_, and M(1,2,3-triazolate)_2_ measured at 300–350 K, in vacuum, and in the dark.

To understand the influence of Fe on the electronic structures of these MOFs, we measured the activation energy (*E*_a_) for each material by collecting current–voltage (I–V) curves between 300 K and 350 K under vacuum and in the dark (Fig. S7–S26†). Plotting the electrical conductivity *versus* temperature for each MOF indicated thermally activated electrical conduction in all cases (Fig. S27†).^28^ The activation energies were extracted by fitting the electrical conductivity–temperature relationships to the Arrhenius law (see ESI†), and are summarized in Fig. 3 and Table S2.† Here again, we found that the Fe analogs exhibit significantly smaller activation energies than the MOFs based on the other metal ions.

**Fig. 4:**
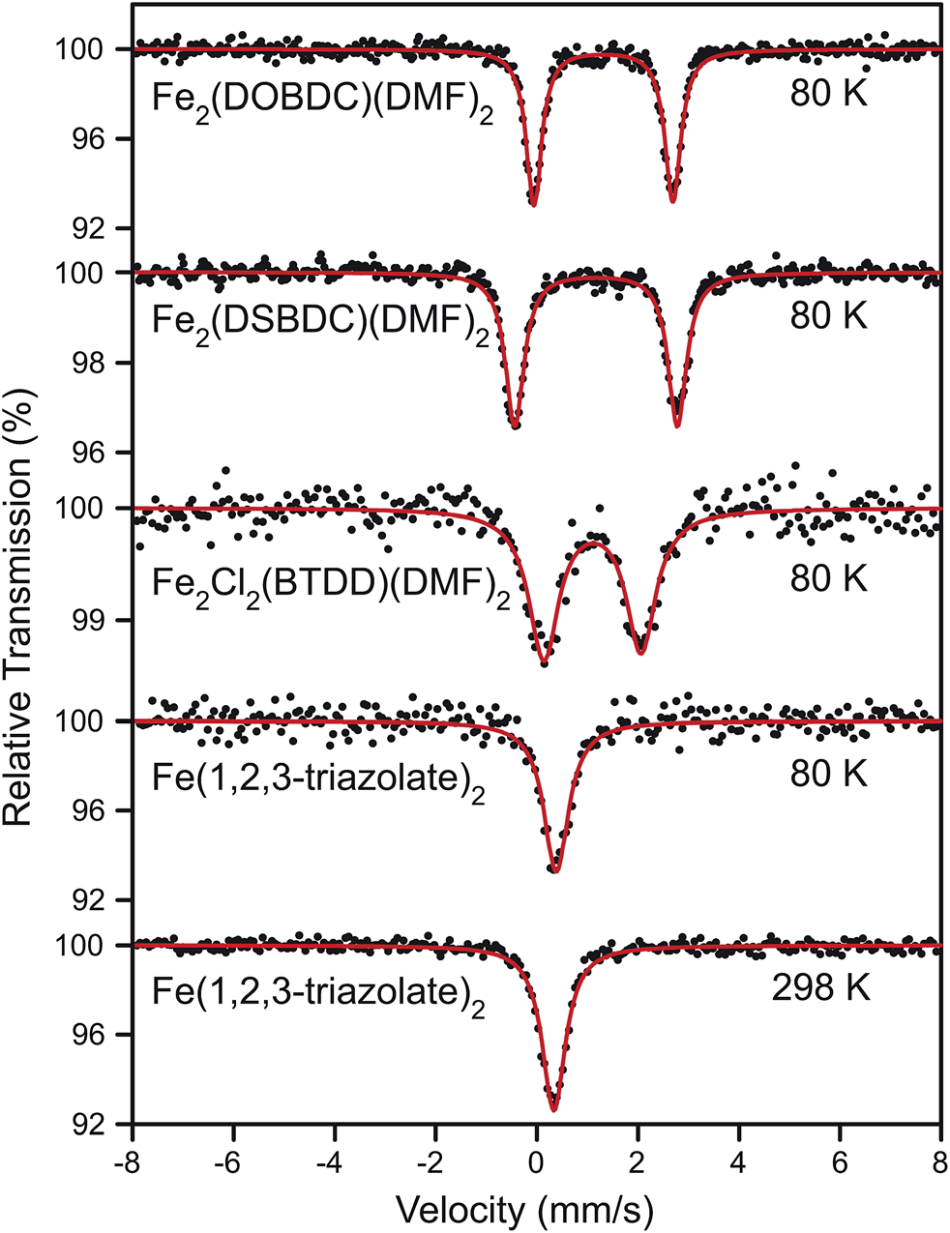
^57^Fe Mössbauer spectra of Fe_2_(DOBDC)(DMF)_2_, Fe_2_(DSBDC)(DMF)_2_, and Fe_2_Cl_2_(BTDD)(DMF)_2_ at 80 K as well as Fe(1,2,3-triazolate)_2_ at 80 and 298 K. All samples were kept in N_2_ atmosphere. Black dots represent experimental data, and red curves represent Lorentzian fitting curves.

Surmising that the oxidation and spin state of the Fe centers could affect electrical conductivity, we investigated all Fe-based MOFs by ^57^Fe Mössbauer spectroscopy. At 80 K, the ^57^Fe Mössbauer spectra of Fe_2_(DOBDC)(DMF)_2_, Fe_2_(DSBDC)(DMF)_2_, and Fe_2_Cl_2_(BTDD)(DMF)_2_ (Fig. 4) display doublets with isomer shifts *δ* = 1.318, 1.172, and 1.099 mm s^–1^, and quadrupole splittings |Δ*E*_Q_| = 2.749, 3.218, and 1.923 mm s^–1^, respectively. These isomer shifts can be unambiguously assigned to high-spin (*S* = 2) Fe^2+^ centers.^29^ At 80 K, the ^57^Fe Mössbauer spectrum of Fe(1,2,3-triazolate)_2_ exhibits a singlet with *δ* = 0.384 mm s^–1^ and no quadrupole splitting. The singlet feature, characteristic of high symmetry (*O*_h_) Fe centers, persists at 298 K although *δ* decreases slightly to 0.336 mm s^–1^ (Fig. 4). Isomer shift values in the range 0.3–0.4 mm s^–1^ can be assigned to either Fe^3+^ or low-spin (*S* = 0) Fe^2+^.^29^ We assign this singlet to low-spin (*S* = 0) Fe^2+^ because elemental analysis for Fe(1,2,3-triazolate)_2_ agrees with a majority of Fe^2+^. However, we cannot rule out the presence of Fe^3+^ that are not detectable by ^57^Fe Mössbauer spectroscopy (under our conditions, we estimate the sensitivity at approximately 1%).

**Fig. 5:**
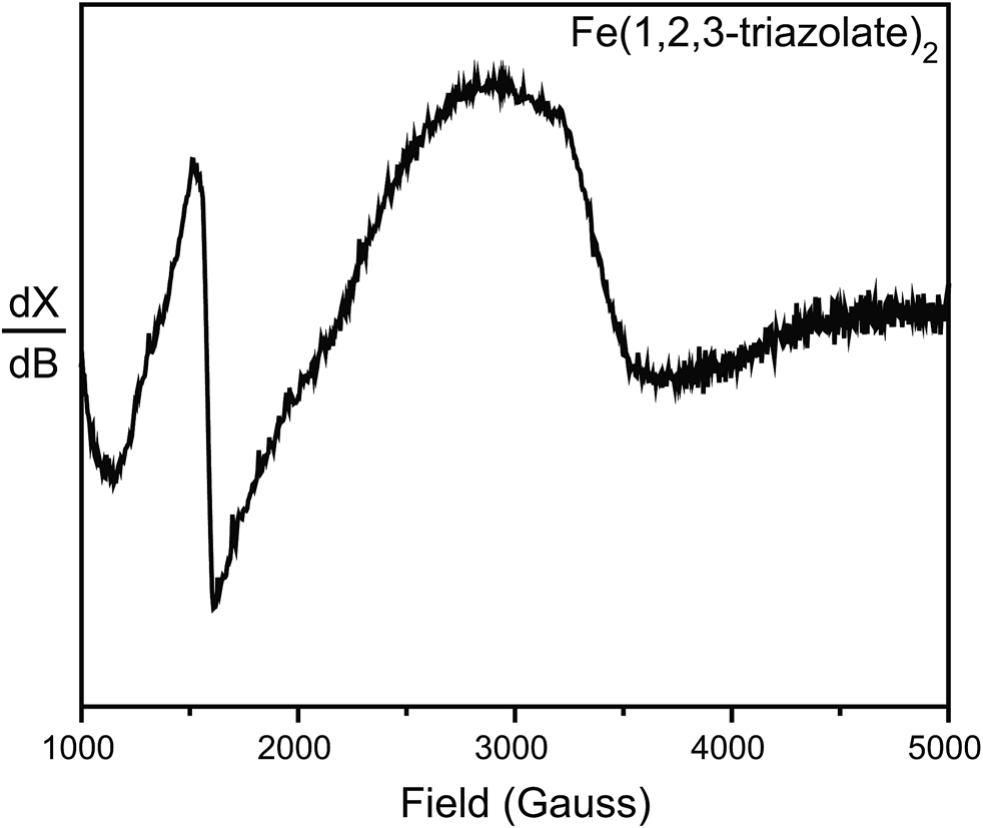
EPR spectrum of Fe(1,2,3-triazolate)_2_ collected at 77 K and in N_2_ atmosphere.

To further probe the possible existence of Fe^3+^, we performed electron paramagnetic resonance (EPR) experiments, which are sensitive to ppm-level concentrations of Fe^3+^ under our conditions. The EPR spectrum of Fe(1,2,3-triazolate)_2_ displayed a broad signal at *g* ≈ 2.0 and a sharp signal at *g* ≈ 4.3 (Fig. 5). These are diagnostic of high-spin (*S* = 5/2) Fe^3+^ centers.^30,31^ Although EPR spectra of Fe_2_(DOBDC)(DMF)_2_, Fe_2_(DSBDC)(DMF)_2_, and Fe_2_Cl_2_(BTDD)(DMF)_2_ revealed only very broad signals, likely due to significant spin–spin relaxation stemming from closely connected high-spin Fe^2+^ ions, these materials are even more air-sensitive than Fe(1,2,3-triazolate)_2_.^22^ It is therefore reasonable to operate under the assumption that all of our Fe MOFs contain Fe^3+^. Indeed, ^57^Fe Mössbauer spectroscopic studies revealed that exposure of Fe_2_(DSBDC)(DMF)_2_ and Fe_2_Cl_2_(BTDD)(DMF)_2_ to air immediately generates a large amount of Fe^3+^ (>70%, Fig. S28 and S29†), whereas exposing Fe(1,2,3-triazolate)_2_ to air for at least one month did not change the isomer shift significantly (*δ* = 0.340 mm s^–1^) (Fig. S30†).

**Fig. 6:**
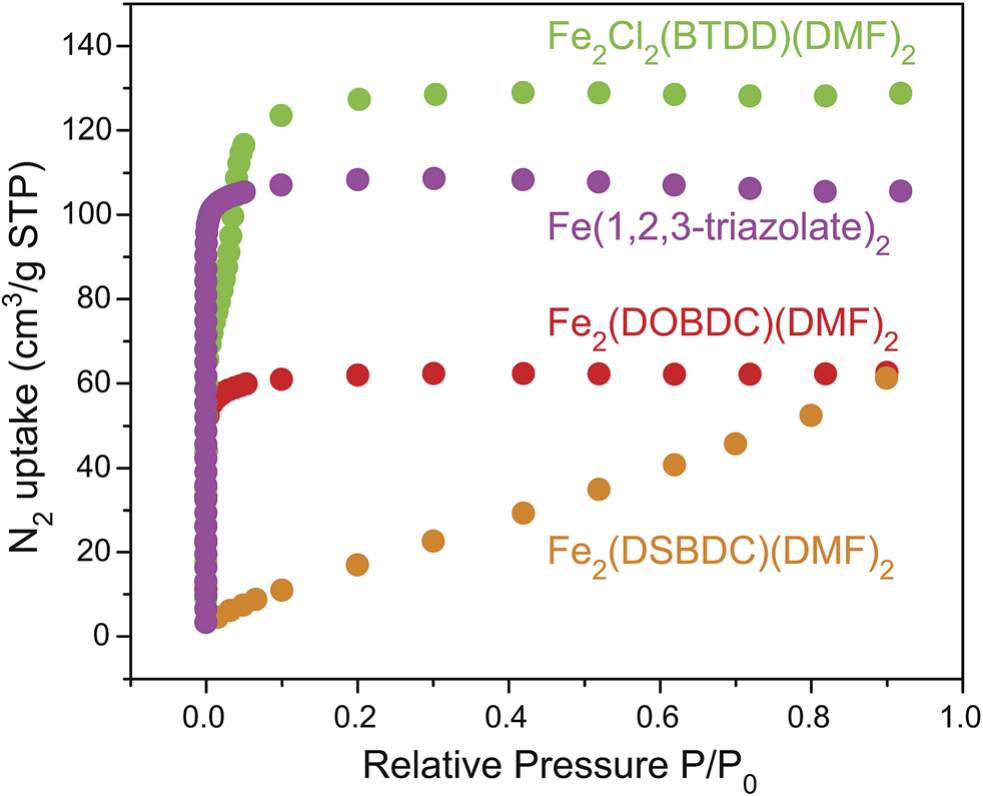
N_2_ adsorption isotherms (77 K) of Fe_2_(DOBDC)(DMF)_2_, Fe_2_(DSBDC)(DMF)_2_, Fe_2_Cl_2_(BTDD)(DMF)_2_, and Fe(1,2,3-triazolate)_2_.

N_2_ sorption measurements for the Fe-based materials revealed Type I isotherms for microporous Fe_2_(DOBDC)(DMF)_2_ and Fe(1,2,3-triazolate)_2_, and a Type IV isotherm for mesoporous Fe_2_Cl_2_(BTDD)(DMF)_2_, with comparatively little gas uptake for Fe_2_(DSBDC)(DMF)_2_ (Fig. 6). The corresponding Brunauer–Emmet–Teller (BET) apparent surface areas for Fe_2_(DOBDC)(DMF)_2_, Fe_2_(DSBDC)(DMF)_2_, Fe_2_Cl_2_(BTDD)(DMF)_2_, and Fe(1,2,3-triazolate)_2_ were 248, 83, 365, and 443 m^2^ g^–1^, respectively (Fig. S31, Table S3†), in line with previous reports and the values expected for each structural type.^5^

## Electronic structure calculations

**Fig. 7:**
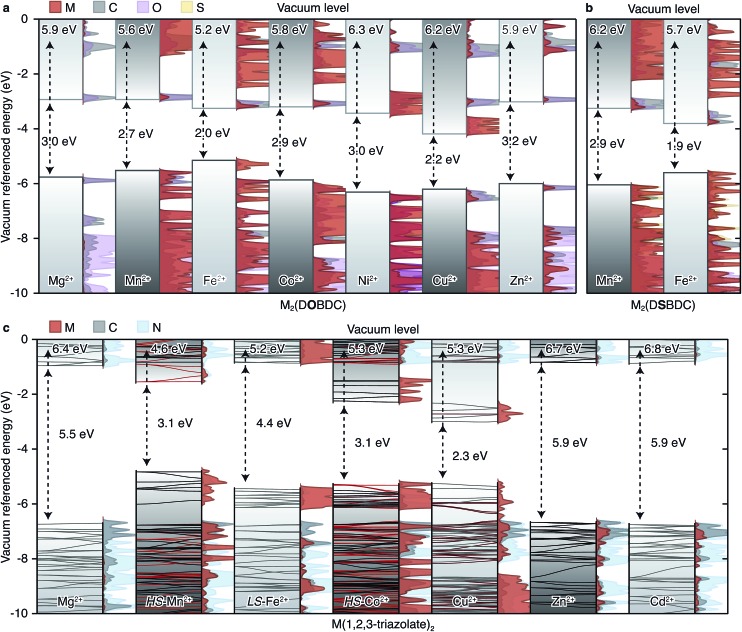
Calculated energy bands and projected density of states (PDOS) of (a) M_2_(DOBDC), (b) M_2_(DSBDC), and (c) M(1,2,3-triazolate)_2_. The electron energies are referenced to the vacuum level using the method presented in ref. 33. *E*_VBM_ are shown on the top and band gaps are shown in the middle of each sub-figure.

To further probe the influence of Fe on the electrical properties of MOFs, we evaluated the electronic structures of the M_2_(DOBDC), M_2_(DSBDC), and M(1,2,3-triazolate)_2_ families using density functional theory (DFT) calculations.§
§For DFT calculations, the coordinating DMF molecules were removed for M_2_(DOBDC) and M_2_(DSBDC). See detailed discussion in ESI.†
 The unit cell of the M_2_Cl_2_(BTDD) family proved too large and we were unable to compute its properties with reasonable computational resources. Owing to the structural similarities between the infinite Fe-based chains in Fe_2_(DEBDC) (E = O, S) and Fe_2_Cl_2_(BTDD) we infer that computational results from the former may be extended to understand the latter. In most cases, our studies yielded intuitive electron energies as presented in Fig. 7.¶
¶The spin states of Mn^2+^ ions in Mn_2_(DOBDC) and Mn(1,2,3,-triazolate)_2_ were reported to be *S* = 5/2.^14,45^ Variable-temperature direct-current magnetic susceptibility measurements for Mn_2_(DSBDC), Co_2_(DOBDC), and Co(1,2,3-triazolate)_2_ also revealed high-spin ground states for the Mn^2+^ and Co^2+^ ions, respectively. See details in the ESI, Fig. S32–S34.†
 One intriguing exception was found for the electronic structure of Co_2_(DOBDC): previous reports computed with the PBEsol functional showed a ground state high-spin (*S* = 3/2) electronic structure. In our hands, PBEsol indeed converges to a high-spin structure, but higher level computational analysis with the HSE06 functional surprisingly revealed the contrary: a high-spin Co^2+^ structure did not converge, and a stable minimum was found only for the low-spin (*S* = 1/2) configuration. This could be due to the systematic differences in equations of state that arise from the use of different functionals.^32^ We could not probe this hypothesis given the extremely expensive calculation required to geometrically optimize the Co^2+^-containing MOF with a hybrid functional.

A summary of the band alignments and accompanying projected density of states (PDOS) of the computed MOFs are presented in Fig. 7. The band structures for the M(1,2,3-triazolate)_2_ materials are superimposed over the schematic band alignment diagrams, to depict the electronic bandwidth. The valence band (VB) maximum energy (*E*_VBM_), conduction band (CB) minimum energy (*E*_CBM_), and band gap (*E*_g_) of each MOF are listed in Table S4.† The energy levels were referenced to an internal vacuum level using a method reported previously.^33^

In the M_2_(DOBDC) family, closed-shell ions, Mg^2+^ and Zn^2+^, contribute little to either VB or CB (Fig. 7a). In contrast, open-shell ions, Mn^2+^, Fe^2+^, Ni^2+^, and Cu^2+^, participate in both VB and CB. More electronegative metal ions, such as Cu^2+^, contribute to a greater extent to the CB and also lower *E*_CBM_, whereas more electropositive metals have greater contribution to the VB and raise *E*_VBM_. For instance, Fe-based orbitals dominate the VB of Fe_2_(DOBDC), which also exhibits the highest E_VBM_ (–5.2 eV) and the smallest band gap (*E*_g_ = 2.0 eV) in this family. Cu-based orbitals dominate the CB of Cu_2_(DOBDC), which exhibits the lowest *E*_CBM_ (–3.9 eV) and the second smallest band gap (*E*_g_ = 2.2 eV). All other MOFs in this family exhibit *E*_g_ of approximately 3 eV. These results are qualitatively consistent with the experimental observation that the activation energy of Fe_2_(DOBDC) is smaller than those of other analogues.

The trends observed for M_2_(DOBDC) are reproduced in the M_2_(DSBDC) family. In Fe_2_(DSBDC) *E*_VBM_ is increased by 0.5 eV and *E*_CBM_ is decreased by 0.5 eV relative to the Mn analog, together giving rise to 1.0 eV difference between the *E*_g_ values of the two materials (Fig. 7b). This is in line with the smaller activation energy observed experimentally for the Fe analog.

In the M(1,2,3-triazolate)_2_ family, closed-shell ions again give bands of different parentage than the open-shell ions. Thus, Mg^2+^, Zn^2+^, and Cd^2+^ do not participate in the VB or CB, which are primarily ligand-based and give rise to similar band gaps for the respective MOFs (*E*_g_ = 5.5–5.9 eV) (Fig. 7c). On the other hand, the PDOS for the Mn^2+^, Co^2+^, and Cu^2+^ analogs show that metal-based orbitals dominate both VB and CB, with negligible contribution from ligand-based orbitals. Charge carriers in these materials must therefore be localized on the metal ions. As in M_2_(DOBDC) and M_2_(DSBDC), *E*_VBM_ and *E*_CBM_ are determined by the electronegativity of the metal ions: Mn(1,2,3-triazolate)_2_ exhibits the highest *E*_VBM_ (–4.6 eV), and Cu(1,2,3-triazolate)_2_ exhibits the lowest *E*_CBM_ (–3.0 eV) and the smallest band gap (*E*_g_ = 2.3 eV). These trends qualitatively agree with the activation energies determined experimentally: the Mg^2+^, Zn^2+^, and Cd^2+^ materials exhibit similar activation energies that are generally higher than those of the open-shell systems.

At first glance, Fe(1,2,3-triazolate)_2_ appears to be anomalous in this family because its computed *E*_g_ is large, which should give rise to high *E*_a_ and low intrinsic electrical conductivity, in direct contrast with its experimentally determined low *E*_a_ and high electrical conductivity. The computational result appears to be particularly unusual given that the Fe^2+^ centers in this material are low-spin (*S* = 0), and are therefore unlikely to contribute high-energy charge carriers. Fe^3+^ ions, however, could provide such charge carriers.

**Fig. 8:**
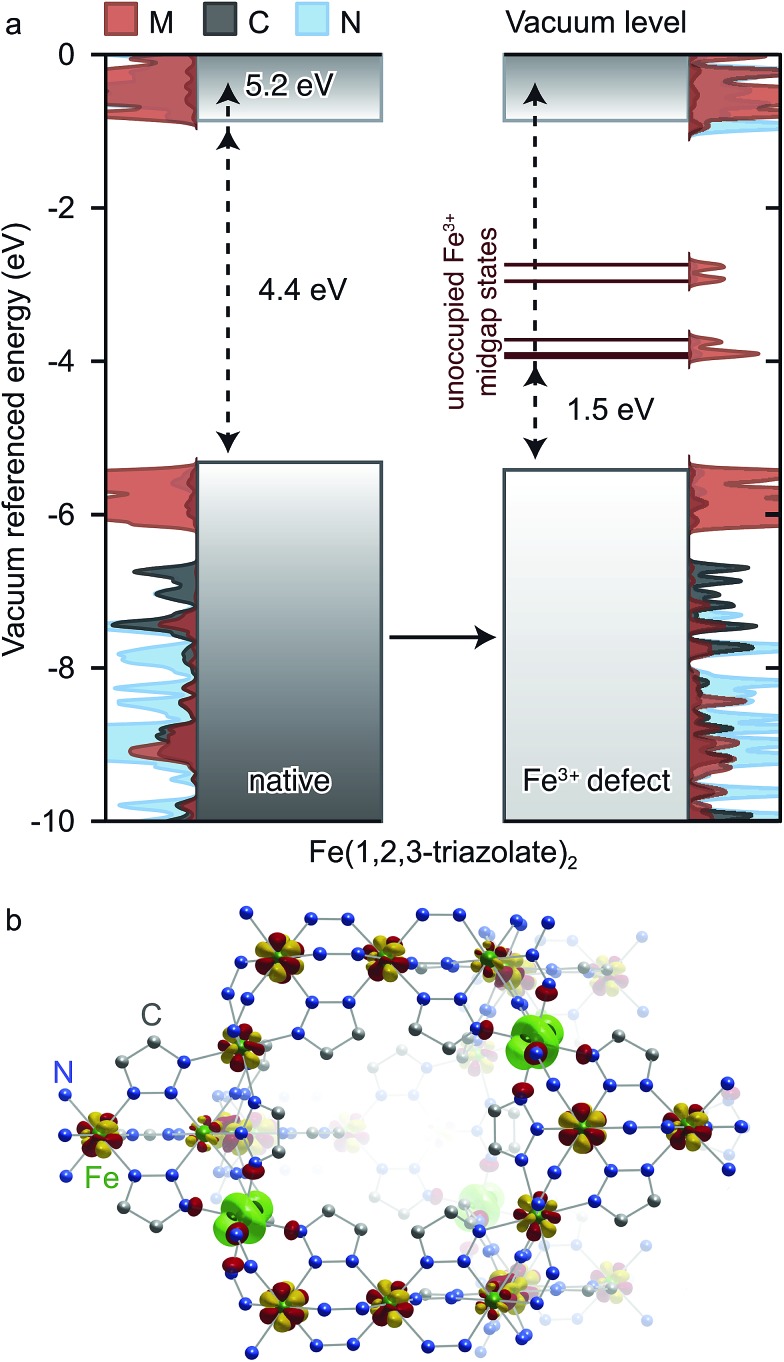
(a) Calculated energy bands and projected density of states (PDOS) of native Fe(1,2,3-triazolate)_2_, and the hypothetical material FeIII1/6FeII5/6(1,2,3-triazolate)_2_^1/6+^. *E*_VBM_ are shown on the top and band gaps are shown in the middle of each sub-figure. (b) The spin density of the hypothetical material FeIII1/6FeII5/6(1,2,3-triazolate)_2_^1/6+^ shows partially delocalized spin across the Fe centers (shown in yellow and red), with some local Fe^3+^ character emphasized in green.

Insight into the effect of Fe^3+^ on the electronic structure of Fe(1,2,3-triazolate)_2_ came from DFT analysis of a hypothetical material FeIII1/6FeII5/6(1,2,3-triazolate)_2_^1/6+^, wherein one sixth of all Fe^2+^ centers are replaced by Fe^3+^. Although this Fe^3+^ concentration is much higher than experimentally observed in Fe(1,2,3-triazolate)_2_, it simply artificially increases the DOS contributions from states arising from Fe^3+^ while simultaneously destabilizing the crystal. We were able to obtain a stable structure at this defect concentration and using a core level alignment we were able to align the defective material to the native Fe^2+^ framework. As shown in Fig. 8a, Fe^3+^ do not significantly affect the energy of the native Fe(1,2,3-triazolate)_2_ bands. Instead, they give rise to mid-gap states attributed to the Fe d-electron spin-down channels. These mid-gap states are found only 1.5 eV above *E*_VBM_. Such redox-accessible states are expected to persist even at much lower Fe^3+^ concentration. As a consequence, VB electrons in Fe^3+^-incorporated Fe(1,2,3-triazolate)_2_ may be thermally activated into the mid-gap Fe-based states, promoting the formation of hole carriers in the VB. In addition, the spin density distribution in this hypothetical material (Fig. 8b) shows that the spins, and equivalently the unpaired electrons, are partially delocalized among Fe centers. The Fe^3+/2+^ mixed valency should facilitate inter-iron charge hopping and improve charge mobility. We therefore attribute the high electrical conductivity of Fe(1,2,3-triazolate)_2_ to the presence of mixed-valent Fe^3+/2+^.

## Discussion

The unique role of Fe in promoting high electrical conductivity across four different families of MOFs that differ in both structure and organic connectivity is highlighted in Fig. 2. Although the particular reasons for this conserved role of Fe across different materials are likely convoluted, Fe stands out among the other metals considered here in several respects. First, among Mg^2+^, Mn^2+^, Fe^2+^, Co^2+^, Ni^2+^, Cu^2+^, Zn^2+^, and Cd^2+^ the ionization energy of Fe^2+^ is the smallest at 30.652 eV (Table S5†).^34^ Second, the standard reduction potential (298 K) of the aqueous Fe^3+/2+^ couple, 0.771 V, is smaller than those of the aqueous Mn^3+/2+^, Co^3+/2+^, and Cu^3+/2+^ couples (Table S5†),^35^ whereas the trivalent states of the other metal ions are essentially inaccessible under similar experimental conditions.‖
‖The inclusion of a Co^3+^ in a natively Co^2+^ framework is not expected to introduce mid-gap states as the defect site would almost certainly be low spin.


Finally, owing to its large ionic radius and small effective nuclear charge, high-spin Fe^2+^ (as found in Fe_2_(DOBDC)(DMF)_2_, Fe_2_(DSBDC)(DMF)_2_, and Fe_2_Cl_2_(BTDD)(DMF)_2_) exhibits the smallest Coulombic attraction between its nucleus and its valence electrons (Table S5†). Together, these suggest that among the metal ions studied here, the valence electrons of high-spin Fe^2+^ have the highest energy. Because Fe orbitals dominate the VB of Fe_2_(DOBDC)(DMF)_2_, Fe_2_(DSBDC)(DMF)_2_, and Fe_2_Cl_2_(BTDD)(DMF)_2_, these high-energy electrons raise the *E*_VBM_ and give rise to small *E*_g_ and *E*_a_ values. This subsequently leads to a higher probability of thermal activation at room temperature and higher charge density than available for the other metal analogs.

The same arguments do not hold for low-spin Fe^2+^. Because low-spin Fe^2+^ and 1,2,3-triazolate do not contribute charge carriers, pure Fe(1,2,3-triazolate)_2_ should accordingly be electrically insulating. This is indeed predicted by DFT calculations, which show that pure Fe(1,2,3-triazolate)_2_ exhibits a larger *E*_g_ than its Mn^2+^, Co^2+^, and Cu^2+^ analogs (Fig. 7c). Instead, we attribute the observed high electrical conductivity of Fe(1,2,3-triazolate)_2_ to the presence of a small amount of Fe^3+^. The presence of Fe^3+^, and thus the formation of a mixed-valence Fe^3+/2+^ system was confirmed by EPR spectroscopy (Fig. 5). Furthermore, DFT calculations suggest that mid-gap states, which effectively lower *E*_a_ and increase electrical conductivity, become available upon forming Fe^3+/2+^ mixed valency in Fe(1,2,3-triazolate)_2_. The presence of Fe^3+^ cannot be ruled out for the high-spin Fe^2+^ materials. The influence of Fe^3+^ would mimic that observed for Fe(1,2,3-triazolate)_2_. Indeed, Fe_2_(DOBDC)(DMF)_2_, Fe_2_(DSBDC)(DMF)_2_, and Fe_2_Cl_2_(BTDD)(DMF)_2_ are significantly more sensitive to O_2_ than Fe(1,2,3-triazolate)_2_, which makes the presence of trace amounts of Fe^3+^ in these materials likely. Because the oxidation potential of the other metals are not as accessible, they are less likely to be mixed valent under our experimental conditions.

## Conclusions

The foregoing results show a critical, conserved role of Fe in promoting high electrical conductivity across four different MOF families comprising twenty different materials and eight different metal ions. In each family, the Fe^2+^-based analog exhibits electrical conductivity and activation energy values that are at least 5 orders of magnitude higher and 0.12–0.54 eV smaller, respectively, than those of materials based on Mg^2+^, Mn^2+^, Co^2+^, Ni^2+^, Cu^2+^, Zn^2+^, and Cd^2+^ ions. Both electronic structure and thermodynamic (*i.e.* redox accessibility) arguments explain the unique role of Fe within these eight metal ions. Similar arguments might provide hints for the design and discovery of electrically conductive MOFs from other metal ions. Most notably, Cr^2+^ is a promising candidate because it has similar ionization energy and Coulombic attraction between its nucleus and valence electrons as Fe^2+^, as well as an accessible Cr^3+/2+^ redox couple (Table S5†).

More generally, our work demonstrates that mixed-valence metal ions improve the electrical conductivity in MOFs. Mixed valency is responsible for the high electrical conductivity in many inorganic solids,^36^ organic conductors,^37,38^ and coordination polymers^39^ because it improves charge density and facilitates charge delocalization. It is also applicable to MOFs, where both metal ions and organic ligands, if redox-active, can lead to mixed-valent states.^40,41^ This has been shown already with two MOFs based on 1,2,4,5-tetrahydroxybenzene and its derivatives, where the ligands coexist in the semiquinone and quinone states, which gives rise to high electrical conductivity (10^–3^ to 10^–1^ S cm^–1^).^42,43^ Therefore, redox-active metal ions and organic ligands are desirable when designing electrically conductive MOFs.

Redox matching between metal ions and organic ligands is also critical to improve electrical conductivity in MOFs.^44^ This requirement is not apparent in the materials studied here because in all four families the ligands are small and neighboring Fe centers have short interatomic distances (<4 Å) such that hopping can occur directly between metal centers. However, in MOFs with large intermetallic separations, organic ligands that have redox couples matched with those of the metal ions may mediate charge hopping. Conversely, redox-inactive or redox-mismatched ligands may block charge hopping. Ligands that facilitate charge transport by participating in hopping (*i.e.* improving metal-to-ligand charge transfer) should therefore be particularly effective in increasing electrical conductivity in MOFs that support mixed valency.
