# Supplementary material for: Is iron unique in promoting electrical conductivity in MOFs?
Source: Chem Sci. 2017 Apr 20;8(6):4450–7. doi: 10.1039/c7sc00647k (PMC5452916; doi:10.1039/c7sc00647k)
Supplement: Supplementary file 1 [file SC-008-C7SC00647K-s001.pdf]

Electronic Supplementary Material (ESI) for Chemical Science.
This journal is © The Royal Society of Chemistry 2017

Supporting Information

Is iron unique in promoting electrical conductivity in MOFs?

Lei Sun,[a] Christopher H. Hendon,[a] Sarah S. Park,[a] Yuri Tulchinsky,[a] Ruomeng Wan,[a] Fang Wang,[a] Aron Walsh,[b,c] and Mircea Dincă*[a]

[a] Department of Chemistry, Massachusetts Institute of Technology, Cambridge, MA, 02139, United States

[b] Department of Materials, Imperial College London, SW7 2AZ London, United Kingdom

[c] Department of Materials Science and Engineering, Yonsei University, Seoul, South Korea

e-mail: mdinca@mit.edu

Table of Contents

Materials S1

Infrared spectroscopy and elemental analysis S1

X-ray diffraction studies S1

Room-temperature electrical conductivity measurements S2

Variable-temperature electrical conductivity measurements S3

^{57}Fe Mössbauer spectroscopy measurements S5

Electron Paramagnetic Resonance S5

Magnetic Measurements S5

Gas sorption measurements S6

Computation details S6

Figure S1. Band structures of $Fe_2(DEBDC)(DMF)_2$ and $Fe_2(DEBDC)$ (E = O, S). S8

$Mg_2(DOBDC)(DMF)_2$ S8

$Co_2(DOBDC)(DMF)_2$ S9

$Ni_2(DOBDC)(DMF)_2$ S9

$Cu_2(DOBDC)(DMF)_2$ S9

$Zn_2(DOBDC)(DMF)_2$ S9

$Mn_2Cl_2(BTDD)(DMF)_2$ S10

$Fe_2Cl_2(BTDD)(DMF)_2$ S10

Co_2Cl_2(BTDD)$(DMF)_2$ S11
Ni_2Cl_2(BTDD)$(DMF)_2$ S11
Mg(1,2,3-triazolate)$_2$ S11
Mn(1,2,3-triazolate)$_2$ S12
Fe(1,2,3-triazolate)$_2$ S12
Co(1,2,3-triazolate)$_2$ S12
Cu(1,2,3-triazolate)$_2$ S12
Zn(1,2,3-triazolate)$_2$ S13
Cd(1,2,3-triazolate)$_2$ S13
Figure S2. Portions of the crystal structure of Mn_2(DSBDC)$(DMF)_2$. S14
Figure S3. PXRD patterns of the powder samples S15
Figure S4. IR spectra S16
Figure S5. PXRD patterns of the pressed pellets S17
Figure S6. Plots of current density (J) versus electrical field strength (E) S18
Table S1. Electrical conductivity S19
Figure S7. I-V curves of Mg_2(DOBDC)$(DMF)_2$ at various temperatures. S20
Figure S8. I-V curves of Mn_2(DOBDC)$(DMF)_2$ at various temperatures. S21
Figure S9. I-V curves of Fe_2(DOBDC)$(DMF)_2$ at various temperatures. S22
Figure S10. I-V curves of Co_2(DOBDC)$(DMF)_2$ at various temperatures. S23
Figure S11. I-V curves of Ni_2(DOBDC)$(DMF)_2$ at various temperatures. S24
Figure S12. I-V curves of Cu_2(DOBDC)$(DMF)_2$ at various temperatures. S25
Figure S13. I-V curves of Zn_2(DOBDC)$(DMF)_2$ at various temperatures. S26
Figure S14. I-V curves of Mn_2(DSBDC)$(DMF)_2$ at various temperatures. S27
Figure S15. I-V curves of Fe_2(DSBDC)$(DMF)_2$ at various temperatures. S28
Figure S16. I-V curves of Mn_2Cl_2(BTDD)$(DMF)_2$ at various temperatures. S29
Figure S17. I-V curves of Fe_2Cl_2(BTDD)$(DMF)_2$ at various temperatures. S30
Figure S18. I-V curves of Co_2Cl_2(BTDD)$(DMF)_2$ at various temperatures. S31
Figure S19. I-V curves of Ni_2Cl_2(BTDD)$(DMF)_2$ at various temperatures. S32
Figure S20. I-V curves of Mg(1,2,3-triazolate)$_2$ at various temperatures. S33
Figure S21. I-V curves of Mn(1,2,3-triazolate)$_2$ at various temperatures. S34
Figure S22. I-V curves of Fe(1,2,3-triazolate)$_2$ at various temperatures. S35
Figure S23. I-V curves of Co(1,2,3-triazolate)$_2$ at various temperatures. S36
Figure S24. I-V curves of Cu(1,2,3-triazolate)$_2$ at various temperatures. S37
Figure S25. I-V curves of Zn(1,2,3-triazolate)$_2$ at various temperatures. S38
Figure S26. I-V curves of Cd(1,2,3-triazolate)$_2$ at various temperatures. S39
Figure S27. Plots of electrical conductivity (σ) versus temperature (T) S40

Table S2. Activation energies S41
Figure S28. ^{57}Fe Mössbauer spectrum of $Fe_2(DSBDC)(DMF)_2$ S42
Figure S29. ^{57}Fe Mössbauer spectrum of $Fe_2Cl_2(BTDD)(DMF)_2$ S43
Figure S30. ^{57}Fe Mössbauer spectrum of Fe(1,2,3-triazolate)$_2$ S44
Figure S31. BET surface area analysis of Fe-based MOFs. S45
Table S3. Consistency criteria analysis of N2 sorption analysis S46
Figure S32. Variable-temperature magnetic susceptibility of Mn_2(DSBDC) S47
Figure S33. Variable-temperature magnetic susceptibility of Co_2(DOBDC)....................................... S48
Figure S34. Variable-temperature magnetic susceptibility of Co(1,2,3-triazolate)$_2$........................... S49
Table S4. Calculation results of M_2(DOBDC), M_2(DSBDC), and M(1,2,3-triazolate)$_2$ S50
Table S5. Properties of the interested 6-coordinated divalent metal ions S51
References S52

Materials

Post-synthetic treatments and characterizations of MOFs were conducted under air-free conditions. Dry and degassed *N*,*N*-dimethylformamide (DMF, VWR), methanol (Sigma Aldrich), dichloromethane (Sigma Aldrich), and toluene (Sigma Aldrich) were obtained by degassing with a vigorous flow of Ar for 45 min and then passing the solvent through two alumina columns in a Glass Contour Solvent System. These solvent were further degassed by freeze-pump-thaw method for three cycles. $Mg(NO_3)_2{\cdot}6H_2O$ (VWR), $MnCl_2{\cdot}4H_2O$ (NOAH Technologies), $Mn(NO_3)_2{\cdot}6H_2O$ (Sigma Aldrich), anhydrous $FeCl_2$ (Strem Chemicals, packed in argon), $CoCl_2{\cdot}6H_2O$ (Alfa Aesar), $NiCl_2{\cdot}6H_2O$ (Fisher Chemicals), $Cu(NO_3)_2{\cdot}3H_2O$ (Sigma Aldrich), $CuCl_2{\cdot}2H_2O$ (Alfa Aesar), $Zn(NO_3)_2{\cdot}6H_2O$ (VWR), $Cd(NO_3)_2{\cdot}4H_2O$ (Fisher Chemicals), hydrochloric acid (VWR), methanol (Sigma Aldrich), ethanol (VWR), tetrahydrofuran (Sigma Aldrich), *N*,*N*-dimethylformamide (VWR), and *N*,*N*-diethylformamide (DEF, Alfa Aesar) were used as received. 1,2,3-triazole (Sigma Aldrich) was degassed by freeze-pump-thaw method for three cycles. 2,5-dihydrylbenzene-1,4-carboxylic acid (H_4DOBDC),[1] 2,5-disulfhydrylbenzene-1,4-dicarboxylic acid (H_4DSBDC),[2] bis(1H-1,2,3-triazolo[4,5-b],[4,'5'-i]dibenzo[1,4]dioxin (H_2BTDD),[3] Mn_2(DOBDC)(DMF)$_2$,[4] Fe_2(DOBDC)(DMF)$_2$,[5] Mn-$_2$(DSBDC)(DMF)$_2$,[6] and Fe_2(DSBDC)(DMF)$_2$[7] were prepared according to the reported procedures.

Infrared spectroscopy and elemental analysis

Infrared spectra were obtained on a Bruker Alpha FT-IR Spectrometer (contained in a N_2-filled glovebox) equipped with a diamond crystal Bruker Platinum ATR accessory. Elemental analysis was performed by Complete Analysis Laboratories, Inc. in Parsippany, NJ, United States or by Robertson Microlit Laboratories, Inc. in Ledgewood, NJ, United States.

X-ray diffraction studies

Powder X-ray diffraction (PXRD) patterns were recorded with a Bruker D8 Advance diffractometer equipped with a θ/2θ Bragg-Brentano geometry and nickel-filtered Cu Kα radiation ($K\alpha_1$ = 1.5406 Å, $K\alpha_2$ = 1.5444 Å, $K\alpha_1/K\alpha_2$ = 0.5). The tube voltage and current were 40 kV and 40 mA, respectively. Samples were prepared on a glass slide in a N_2-filled glovebox. The slide was placed inside an airtight specimen holder ring with a dome-like X-ray transparent cap. Background of PXRD patterns was corrected by Bruker Diffrac.Suite EVA software.

Room-temperature electrical conductivity measurements

A home-built instrument (*in situ* press) was used to make 2-contact-probe devices of pressed pellets *in situ* in a N_2-filled glovebox for room-temperature electrical conductivity measurements.[8,9] This home-built instrument consists of two polymer-supported stainless steel rods and a glass tube (Ace glass). The diameter of the rods and the inner diameter of the tube are the same (diameter is 0.208 cm). During the measurement, we placed one rod into the tube, kept the other end of the rod at the middle of the tube, added the powder of a MOF into the tube, inserted the other rod, and used a clamp to press the two rods from two sides. This allowed pressing the powder into a dense pellet, with contacts between rods and the pellet made *in situ*. The applied pressure was approximately 200 MPa. The diameter of the pellet (d) was the same with the inner diameter of the tube, in this case 0.208 cm. The area of the pellet was 0.0338 cm^2 calculated based on its diameter. We connected the two rods to electrical plugs of the glove box by alligator-equipped copper wires. The electrical plugs were further connected to a sourcemeter (Keithley model 2450) through test leads (Keithley model 8608) for Fe^{2+}-based MOFs or to an electrometer (Keithley model 6517B) through a triax cable (Keithley model 237-ALG-2) and a banana cable (Keithley model 8607) for other MOFs. Keithley 6517B electrometer was able to measure highly resistive materials thanks to its high inner impedance (approximately 10^{16} Ω), so it was chosen to measure the electrical conductivity of Mg-, Mn-, Co-, Ni-, Cu-, Zn-, and Cd-based materials to achieve accurate results. Temperature was not controlled, and we assumed the temperature of the pressed pellets to be the same with room temperature, which was 300 K during the electrical measurements. The pellets were kept in the N_2-filled glove box and in the dark during the electrical measurements. Current−voltage (I−V) curves were scanned at various voltage ranges that were selected according to the resistance of the pellet and the sensitivity of the instrument. All I−V curves were linear, verifying Ohmic contacts between the rods and the pellet. After each electrical measurement, we disassembled the *in situ* press instrument. The pellet remained inside the glass tube. We removed the pellet from the tube by a long stainless steel rod, measured its thickness by a micrometer (Mitutoyo), and measured the powder X-ray diffraction pattern of each pellet to confirm that the MOF retained its structure. The thickness of the pellets ranges from 200 μm to 1 mm depending on the amount of the added sample. Blank experiment was also performed by replacing the powder of MOFs with N_2. The observed current was a complete noise and was below 10 fA under a voltage scanned between –100 V

and 100 V. Therefore, the glass tube, the N_2 gas, and the supporting polymer are insulating enough that they do not affect electrical measurements of MOFs.

According to Ohm's law, current-density−electric-field-strength (J−E) curve was used to calculate conductivity,

$$\sigma = \frac{J}{E}, J = \frac{I}{A}, E = \frac{V}{t}$$

where σ is electrical conductivity, J is current density, E is electric field strength, I is current, V is voltage, A is the area of the pellet, and t is the thickness of the pellet. To correct the random error, we extracted electrical conductivity by linear regression fitting of the J−E curves. The advantage of J−E curves versus I−V curves is that J−E curves normalize the thickness and area of pellets so that they clearly show different conductivity values of different materials.

Because conductivity of Fe^{2+}-based MOFs and other MOFs are very different, we plotted J−E curves in log scale (Fig. S6),

$$\lg(J) = \lg(\sigma) + \lg(E)$$

Therefore, in log scale, the slope of each J−E curve is 1, and the intercept on the y-axis is lg(σ).

Variable-temperature electrical conductivity measurements

We used a home-built setup (*in situ* screw press) to make 2-contact-probe devices of pressed pellets of samples in a N_2-filled glovebox *in* situ for variable-temperature electrical conductivity measurements. This setup consists of two screws made of stainless steel, a short tube made of Garolite-10, and a sample mounting chuck of a miniature transfer chamber of a probe station (Janis Cryogenics). One screw is longer than the other one. The tube has screw threads that fit the two screws on its inner wall. First, we screwed the short screw into the tube to close one end, and brought these two parts into a N_2-filled glovebox together with the long screw and the sample mounting chuck. In a N_2-filled glovebox, we added the powder of a MOF into the above tube, screwed the long screw into the open end of the tube, and tightened the two screws simultaneously to make a pressed pellet, with contacts between screws and the pellet made *in situ*. The applied pressure was approximately 50 MPa. The long screw protruded out of the tube. The whole setup was then mounted onto the sample mounting chuck, which was made of OFHC copper. By this way, both electrical and thermal connections were established between the long screw and the sample mounting chuck, so

the sample mounting chuck could be used as an electrode. The short screw was used as the second electrode. This setup allows us to measure electrical conductance of air-sensitive samples with 2-contact probe method safely and conveniently, and consumed very little sample (~ 3 mg). Up to eight samples can be loaded at once.

The above sample mounting chuck was part of a Janis probe station patent pending transportable miniature transfer chamber (Janis Cryogenics). The whole *in situ* screw press setup was transferred into the probe station chamber through the miniature transfer chamber to keep the samples from air. Electrical contacts were made by touching the short screw or the inner plate of the sample mounting chuck by gold-coated tungsten probes (Janis 7B-100G). Probes were connected to a sourcemeter (Keithley model 2450) through triax cables (Keithley model 7078-TRX-10) for Fe^{2+}-based MOFs or to an electrometer (Keithley model 6517B) through a triax cable (Keithley model 7078-TRX-10) and a triax-to-banana plug (Keithley model 237-BAN-3A) for other MOFs. Temperature was balanced by the heater of the probe station chuck and liquid nitrogen, and was controlled by a temperature controller (Scientific Instruments model 9700). All samples were measured at 300 − 350 K with 10 K interval, in vacuum, and in the dark. An I−V curve was collected at each temperature at various voltage range according to the resistance of the pellet and the sensitivity of the electronic instrument. At least five I–V points were collected for each I–V curve. All I−V curves were linear, verifying Ohmic contacts between screws and the pellet. The electrical conductance of MOFs was extracted by linear regression fitting of the I−V curves. The thickness and area of pellets were difficult to measure due to the irregular pellet shape. Therefore, the electrical conductivity at 300 K was normalized to the values obtained by the abovementioned room-temperature electrical conductivity measurements (the last section) in the N_2-filled glovebox. The electrical conductivity at other temperatures was scaled to the value at 300 K. Activation energy was calculated based on Arrhenius law,

$$\sigma = \sigma_0 \exp(-\frac{E_a}{kT})$$

where σ is electrical conductivity, E_a is activation energy, k is Boltzmann constant, T is absolute temperature, and σ_0 is a prefactor. When plotting in $\lg(\sigma)$ vs. $\frac{1}{T}$ (Fig. S28), the slope of the curve is $-\frac{\lg(e)}{\lg(10)} \times \frac{E_a}{k}$, so the activation energy E_a can be extracted.

^{57}Fe Mössbauer spectroscopy measurements

All Fe-based MOFs were synthesized under air-free conditions, stored in a N_2-filled glovebox, kept intact to air when they were being transferred into the chamber of the Mössbauer spectrometer, and measured at 80 K under air-free conditions. Fe(1,2,3-triazolate)$_2$ was warmed up to 298 K inside the chamber of the Mössbauer spectrometer, and was measured again. In addition, powder samples of Fe_2(DSBDC)(DMF)$_2$ and Fe_2Cl_2(BTDD)(DMF)$_2$ were exposed to air for less than 5 seconds and were measured at 80 K and 298 K, respectively. A sample of Fe(1,2,3-triazolate)$_2$ was kept in air for a month and was measured at 298 K.

Solid samples of Fe^{2+}-based MOFs were suspended in Apiezon M grease and placed inside a nylon sample holder in a N_2-filled glovebox. The Mössbauer spectra were then recorded on an MSI spectrometer (WEB Research Co.) using a ^{57}Co source in a Rh matrix kept at room temperature. Velocity calibration was achieved using metallic iron foil at room temperature. Isomer shift (δ) values are reported with respect to the metallic iron (δ = 0.00 mm/s). The spectra were fitted with Lorentzian lines.

Electron Paramagnetic Resonance

EPR spectra were collected using Bruker EleXsys E-500 CW-EPR spectrometer. About 5 mg of sample was placed in toluene and was sealed in a low pressure/vac Suprasil EPR tube (Wilmad-Labglass) in a N_2 glovebox. The spectra were measured at 77 K with a microwave power of 0.20 mW and frequency of 9.423 GHz.

Magnetic Measurements

Magnetic susceptibility data were collected using a Quantum Design MPMS-XL SQUID magnetometer. A gelatin capsule was filled with Mn_2(DSBDC), Co_2(DOBDC), or Co(1,2,3-triazolate)$_2$, which were completely desolvated with literature procedures, respectively.[6,10,11] Direct-current (DC) magnetic susceptibility measurements were obtained under a DC field of 1 T for Mn_2(DSBDC) and Co_2(DOBDC) and 1000 Oe for Co(1,2,3-triazolate)$_2$ between 2 K and 300 K. All data were corrected for diamagnetic contributions from the capsule. Pascal's constants were used to account for diamagnetic correction for the sample itself.[12] The product of molar magnetic susceptibility (χ_M) and absolute temperature (T) at room temperature (300 K) was used to calculate effective magnetic moment (μ_B):

$$\mu_{eff} = 2.828\sqrt{\chi_M T}\, B.M.$$

Gas sorption measurements

A Micromeritics ASAP 2020 Surface Area and Porosity Analyzer was used to measure nitrogen adsorption isotherms. An oven-dried sample tube equipped with a TranSeal™ (Micromeritics) was evacuated and tared. The sample was transferred to the sample tube, which was then capped by a TranSeal™. The sample was heated to 100 °C and held at this temperature until the outgas rate was less than 2 mtorr/minute. The evacuated sample tube was weighed again and the sample mass was determined by subtracting the mass of the previously tared tube. An N_2 adsorption isotherm was measured using a liquid nitrogen bath (77 K). Ultra high purity grade (99.999% purity) N_2 and He, oil-free valves and gas regulators were used for all free space corrections and measurements.

Computation details

All electronic and structural calculations were performed within the Kohn−Sham density functional theory (DFT) framework as implemented in *Vienna ab initio simulation package* (VASP), a plane-wave basis set code (with PAW scalar-relativistic pseudopotentials). Starting with the experimentally determined unit cells for the M_2(DOBDC), M_2(DSBDC), and M(1,2,3-triazolate)$_2$ frameworks, the lattice parameters and atomic positions were relaxed with the semi-local Perdew−Burke−Ernzerhof exchange−correlation functional revised for solids (PBEsol) using Gamma point only sampling of the Brillouin zone for each of the materials. A 500 eV plane-wave cutoff was found to be suitable for convergence of electronic wave functions to give total energies within 0.01 eV/atom. In circumstances where magnetic ordering was possible (*i.e.* spin polarized materials) a ferromagnetic arrangement was invoked, to minimize the magnetic unit cell. This approximation is reasonable given the typically very low temperatures for magnetic ordering in MOFs, and is not expected to alter the computed electronic properties dramatically.[13]

The key electronic properties, including electron density, electrostatic potential, and band gap, were computed using a hybrid exchange−correlation functional (HSE06) with 25% of the short- range semi-local exchange replaced by the exact nonlocal Hartree−Fock exchange. The band structures were calculated by explicit sampling at high symmetry points according to the Bilbao Crystallographic Server Database. A single point HSE06 calculation was then performed on each optimized structure to obtain more accurate electronic properties.

In contrast to molecular quantum-chemical calculations, within periodic boundary conditions, the electronic eigenvalues resulting from the solution of the Kohn−Sham equations are given with respect to an internal reference (for VASP it is the average electrostatic potential of the repeating cell). The consequence is that absolute values of band energies cannot be compared between two or more frameworks: there is no common vacuum level. It should be noted that for solids, unlike finite systems, the highest occupied Kohn−Sham eigenvalue and the electron removal energy ($N \rightarrow N - 1$ system) are equivalent in the dilute limit.

For the reference electrostatic potential we use a spherical average of the Hartree potential in a sphere of r = 2 Å with an origin at the center of the pore. The analysis code for this calculation, which can also calculate planar and macroscopic averages of electrostatic potentials and charge densities, is freely available (https://github.com/WMD-Bath/MacroDensity).

In the M_2(DOBDC) and M_2(DSBDC) families, the coordinating DMF molecules were not included in the calculation because they greatly increase the calculation time and the difficulty of geometrical optimization due to the free rotation around the M-O_{DMF} bond. Indeed, the inclusion of coordinating DMF appears to systematically increase the E_{VBM}. From our previous publication[7] we computed the electronic structures of M_2(DEBDC) (M = Mn, Fe; X = O, S) with and without explicit coordinated DMF. The electron energy level alignment is presented in Fig. S1. We note that the electrostatic potential alignment requires a stable potential plateau in the pore void. In materials with coordinated DMF, the potential reaches a stable constant, but the pore volume is substantially diminished. Therefore, the rigid shift in work function towards the vacuum level could be attributed to two compounding factors: i) a misalignment of the reference potential, as the process is dependent on the electrostatic potential variance, which is further dependent on establishing a suitably large pore (the explicit DMF reduces porosity to the limit of our confidence in the alignment procedure), and ii) the chemical differences associated with DMF binding. It is impossible to decouple them, and hence to minimize these variables we elected to discuss the trends for the DMF-free M_2(DOBDC) and DMF-free M_2(DSBDC). Importantly, the omission of DMF augments the d-energy level splitting, but should not grossly affect the framework. We do note that this could be the reason HSE06 calculations do not produce a stable high-spin minimum energy

structure of DMF-free Co_2(DOBDC), but it could also be attributed to the negative pressure installed by the HSE06 functional when used as a single point on the PBEsol structure.

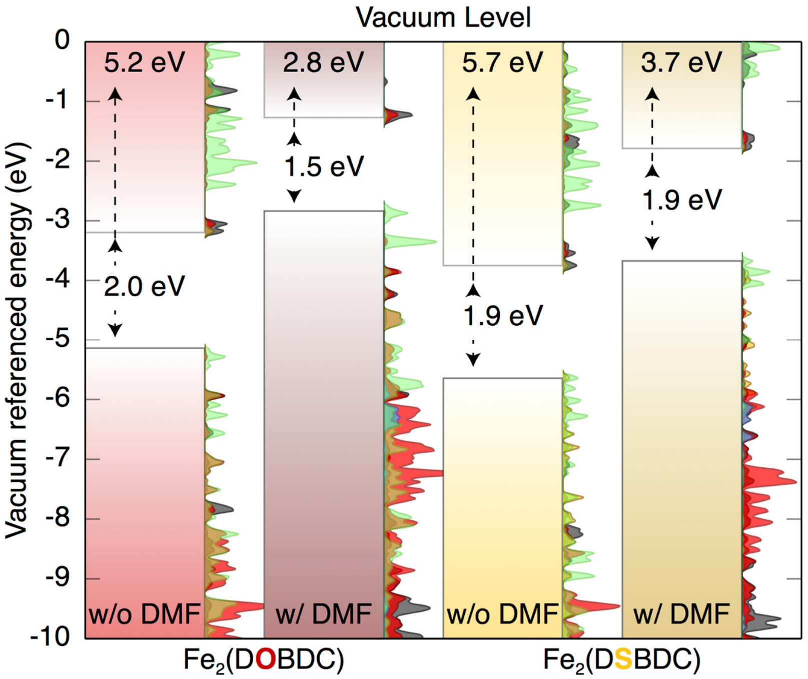

Figure S1. Band structures of Fe_2(DEBDC)$(DMF)_2$ and Fe_2(DEBDC) (E = O, S). The inclusion of metal-coordinated DMF results in a systematic decrease in work function.

The Fe^{3+} defective triazolate framework was computed by removing $1e^-$ from the native Fe^{2+} framework. Because the periodic cell obtains a formal charge, the work function alignment is no longer applicable. Instead, we elected to align the work function based on the relative C-*s* core-level alignment to the native Fe^{2+} material. Using this technique, we are able to recover a reasonable E_{VBM} with very similar atomic contributions to the frontier bands (Fig. 8). In other words, we have confidence in the vacuum alignment of the neutral material, and confidence in the core-electron energies of a remote carbon atom. Thus, we are able to estimate with reasonable confidence the energies of the Fe^{3+} mid-gap states.

Mg_2(DOBDC)$(DMF)_2$

Mg_2(DOBDC) was prepared according to a reported procedure.[14] The as-synthesized Mg_2(DOBDC) was soaked in 10 mL of dry and degassed DMF for three times and successively 10 mL of dry and degassed DCM for three times, and was evacuated under vacuum at 100 °C for 2 h. Elemental analysis calcd. For $Mg_2(C_8H_2O_6)(C_3H_7NO)_2$: C, 43.23; H, 4.15; N, 7.20. Found:

C, 43.28; H, 4.17; N, 6.98. IR (Diamond-ATR, cm^{-1}): 2925 (w), 2888 (sh), 1664 (s), 1576 (s), 1498 (w), 1474 (w), 1454 (w), 1418 (s), 1393 (w), 1370 (w), 1353 (w), 1232 (w), 1214 (s), 1114 (w), 1103 (w), 1060 (w), 911 (w), 886 (w), 829 (w), 818 (w), 677 (w), 641 (w), 581 (w).

Co_2(DOBDC)(DMF)$_2$

Co_2(DOBDC) was prepared according to a reported procedure.[15] The as-synthesized Co_2(DOBDC) was soaked in 10 mL of dry and degassed DMF for three times and successively 10 mL of dry and degassed DCM for three times, and was evacuated under vacuum at 100 °C for 2 h. Elemental analysis calcd. For $Co_2(C_8H_2O_6)(C_3H_7NO)_2(CH_2Cl_2)_{0.8}$: C, 33.79; H, 3.37; N, 5.32. Found: C, 33.42; H, 3.30; N, 5.61. IR (Diamond-ATR, cm^{-1}): 2928 (w), 2888(sh), 1657 (s), 1552 (s), 1438 (sh), 1410 (s), 1366 (w), 1240 (w), 1198 (s), 1118 (w), 1101 (w), 1060 (w), 910 (w), 884 (w), 812 (s), 676 (w), 633 (w), 581 (w).

Ni_2(DOBDC)(DMF)$_2$

Ni_2(DOBDC) was prepared according to a reported procedure.[16] The as-synthesized Ni_2(DOBDC) was soaked in 10 mL of dry and degassed DMF for three times and successively 10 mL of dry and degassed DCM for three times, and was evacuated under vacuum at 100 °C for 2 h. Elemental analysis calcd. For $Ni_2(C_8H_2O_6)(C_3H_7NO)_2$: C, 36.73; H, 3.52; N, 6.12. Found: C, 36.81; H, 3.52; N, 6.03. IR (Diamond-ATR, cm^{-1}): 2925 (w), 2884 (sh), 1655 (s), 1560 (s), 1497 (w), 1446 (w), 1410 (s), 1362 (w), 1236 (w), 1202 (s), 1117 (w), 1103 (w), 1060 (w), 910 (w), 887 (w), 825 (w), 813 (w), 679 (w), 641 (w), 588 (w).

Cu_2(DOBDC)(DMF)$_2$

Cu_2(DOBDC) was prepared according to a reported procedure.[17] The as-synthesized Cu_2(DOBDC) was soaked in 10 mL of dry and degassed DMF for three times and successively 10 mL of dry and degassed DCM for three times, and was evacuated under vacuum at 100 °C for 2 h. Elemental analysis calcd. For $Cu_2(C_8H_2O_6)(C_3H_7NO)_2(CH_2Cl_2)_{0.35}$: C, 34.67; H, 3.39; N, 5.64. Found: C, 35.07; H, 3.59; N, 5.23. IR (Diamond-ATR, cm^{-1}): 2926 (w), 2875 (w), 1650 (s), 1545 (s), 1505 (sh), 1447 (w), 1417 (s), 1385 (w), 1246 (w), 1192 (s), 1118 (w), 1097 (w), 1063 (w), 890 (w), 828 (w), 801 (w), 788 (w), 665 (w), 648 (w), 585 (w).

Zn_2(DOBDC)(DMF)$_2$

Zn_2(DOBDC) was prepared according to a reported procedure.[18] The as-synthesized Zn_2(DOBDC) was soaked in 10 mL of dry and degassed DMF for three times and successively

10 mL of dry and degassed DCM for three times, and was evacuated under vacuum at 100 °C for 2 h. Elemental analysis calcd. For $Zn_2(C_8H_2O_6)(C_3H_7NO)_2$: C, 35.69; H, 3.42; N, 5.95. Found: C, 35.64; H, 3.25; N, 5.60. IR (Diamond-ATR, cm^{-1}): 2928 (w), 1652 (s), 1554 (s), 1497 (w), 1444 (sh), 1411 (s), 1366 (w), 1295 (w), 1240 (w), 1196 (w), 1117 (w), 1103 (w), 1060 (w), 911 (w), 881 (w), 812 (w), 675 (w), 577 (w).

Mn_2Cl_2(BTDD)(DMF)$_2$

Mn_2Cl_2(BTDD) was prepared according to a reported procedure.[19] The as-synthesized Mn_2Cl_2(BTDD) was soaked in 10 mL of dry and degassed DMF for three times and successively 10 mL of dry and degassed DCM for three times, and was evacuated under vacuum at 100 °C for 2 h. Elemental analysis calcd. For $Mn_2Cl_2(C_{12}H_4N_6O_2)(C_3H_7NO)_2(CH_2Cl_2)_{0.5}$: C, 35.06; H, 3.02; N, 17.68. Found: C, 34.68; H, 2.77; N, 17.66. IR (Diamond-ATR, cm^{-1}): 3080 (w), 2926 (w), 1651 (s), 1569 (w), 1458 (s), 1419 (w), 1379 (w), 1343 (s), 1287 (w), 1226 (w), 1175 (s), 1152 (w), 1110 (w), 1061 (w), 914 (w), 856 (w), 804 (w), 774 (w), 677 (w), 523 (w).

Fe_2Cl_2(BTDD)(DMF)$_2$

Inside a N_2-filled glovebox a 100 ml Schlenk flask was loaded with 48.0 mg (0.38 mmol) of anhydrous $FeCl_2$. The flask was then capped with a rubber septum, removed from the glovebox and flushed with Ar. 50 ml of EtOH, previously degassed by three successive freeze-pump-thaw cycles, and 1.5 ml of similarly degassed concentrated HCl, were then cannula-transferred into the flask forming a nearly colorless clear solution. Another Schlenk flask was loaded with 50.0 mg (0.19 mmol) of H_2BTDD and 50 ml of DMF (not anhydrous), and heated to 130°C until a clear solution was obtained. The obtained solution was then cooled to room temperature, degassed by three successive freeze-pump-thaw cycles, and cannulated into the first Schlenk flask. This was then tightly capped with a Teflon screw-cap and kept at 65°C for 4 – 5 days, during which a yellow crystalline precipitate has formed. The precipitate was then collected on a glass frit by vacuum-filtration under Ar, washed with degassed DMF and MeOH, and briefly dried under vacuum, resulting in a light yellow crystalline powder. The whole synthesis must be performed under rigorously air-free conditions, since even a trace amount of O_2 results in an immediate oxidation, as seen by change of the color from yellow to dark brown.

The as-synthesized Fe_2Cl_2(BTDD) was transferred into a N_2-filled glovebox. It was soaked in 10 mL of dry and degassed DMF for three times and successively 10 mL of dry and degassed DCM

for three times, and was evacuated under vacuum at 100 °C for 2 h. Elemental analysis calcd. For $Fe_2Cl_2(C_{12}H_4N_6O_2)(C_3H_7NO)_2(CH_2Cl_2)_{0.6}$: C, 34.69; H, 3.01; N, 17.40. Found: C, 34.71; H, 3.06; N, 17.76. IR (Diamond-ATR, cm^{-1}): 3080 (w), 2939 (w), 1648 (s), 1572 (w), 1455 (s), 1375 (w), 1345 (s), 1237 (w), 1182 (s), 1111 (w), 1059 (w), 915 (w), 855 (w), 805 (w), 736 (w), 702 (w), 682 (w), 533 (w).

Co_2Cl_2(BTDD)(DMF)$_2$

Co_2Cl_2(BTDD) was prepared according to a reported procedure.[19] The as-synthesized Co_2Cl_2(BTDD) was soaked in 10 mL of dry and degassed DMF for three times and successively 10 mL of dry and degassed DCM for three times, and was evacuated under vacuum at 100 °C for 2 h. Elemental analysis calcd. For $Co_2Cl_2(C_{12}H_4N_6O_2)(C_3H_7NO)_2(CH_2Cl_2)_{0.5}$: C, 34.63; H, 2.99; N, 17.46. Found: C, 34.68; H, 2.78; N, 17.62. IR (Diamond-ATR, cm^{-1}): 3091 (w), 2935 (w), 1654 (s), 1570 (w), 1460 (s), 1380 (w), 1348 (s), 1230 (w), 1188 (s), 1105 (w), 918 (w), 858 (w), 804 (w), 680 (w), 533 (w).

Ni_2Cl_2(BTDD)(DMF)$_2$

Ni_2Cl_2(BTDD) was prepared according to a reported procedure.[19] The as-synthesized Ni_2Cl_2(BTDD) was soaked in 10 mL of dry and degassed DMF for three times and successively 10 mL of dry and degassed DCM for three times, and was evacuated under vacuum at 100 °C for 2 h. Elemental analysis calcd. For $Ni_2Cl_2(C_{12}H_4N_6O_2)(C_3H_7NO)_2(CH_2Cl_2)$: C, 33.38; H, 2.95; N, 16.39. Found: C, 33.13; H, 2.77; N, 16.36. IR (Diamond-ATR, cm^{-1}): 2951 (w), 1654 (s), 1572 (w), 1460 (s), 1385 (w), 1348 (s), 1298 (w), 1237 (w), 1193 (s), 1103 (w), 920 (s), 857 (w), 805 (w), 682 (w), 537 (w).

Mg(1,2,3-triazolate)$_2$

Mg(1,2,3-triazolate)$_2$ was prepared according to a reported procedure.[11] The as-synthesized Mg(1,2,3-triazolate)$_2$ was soaked in 10 mL of dry and degassed DMF for three times and successively 10 mL of dry and degassed DCM for three times, and was evacuated under vacuum at 100 °C for 2 h. Elemental analysis calcd. For $Mg(C_2H_2N_3)_2$: C, 29.94; H, 2.51; N, 52.39. Found: C, 29.72; H, 2.58; N, 52.25. IR (Diamond-ATR, cm^{-1}): 2885 (w), 1628 (s), 1454 (w), 1409 (w), 1349 (s), 1189 (w), 1110 (w), 979 (w), 798 (w), 731 (w).

Mn(1,2,3-triazolate)$_2$

Mn(1,2,3-triazolate)$_2$ was prepared according to a reported procedure.[11] The as-synthesized Mn(1,2,3-triazolate)$_2$was soaked in 10 mL of dry and degassed DMF for three times and successively 10 mL of dry and degassed DCM for three times, and was evacuated under

vacuum at 100 °C for 2 h. Elemental analysis calcd. For $Mn(C_2H_2N_3)_2$: C, 25.14; H, 2.11; N, 43.99. Found: C, 25.24; H, 2.08; N, 43.87. IR (Diamond-ATR, cm^{-1}): 3141 (w), 1723 (w), 1654 (w), 1623 (w), 1457 (w), 1420 (w), 1224 (w), 1202 (w), 1178 (s), 1097 (s), 993 (w), 974 (s), 795 (s), 721 (w).

Fe(1,2,3-triazolate)$_2$

Fe(1,2,3-triazolate)$_2$ was prepared according to a reported procedure.[11] The as-synthesized Fe(1,2,3-triazolate)$_2$ was soaked in 10 mL of dry and degassed DMF for three times and successively 10 mL of dry and degassed DCM for three times, and was evacuated under vacuum at 100 °C for 2 h. Elemental analysis calcd. For $Fe(C_2H_2N_3)_2$: C, 25.02; H, 2.10; N, 43.79. Found: C, 25.21; H, 2.85; N, 43.80. IR (Diamond-ATR, cm^{-1}): 3153 (w), 1685 (w), 1621 (w), 1565 (w), 1555 (w), 1475 (w), 1421 (w), 1382 (w), 1275 (w), 1227 (w), 1190 (w), 1127 (s), 1110 (w), 1001 (w), 981 (w), 781 (s), 728 (w),

Co(1,2,3-triazolate)$_2$

Co(1,2,3-triazolate)$_2$ was prepared according to a reported procedure.[11] The as-synthesized Co(1,2,3-triazolate)$_2$ was soaked in 10 mL of dry and degassed DMF for three times and successively 10 mL of dry and degassed DCM for three times, and was evacuated under vacuum at 100 °C for 2 h. Elemental analysis calcd. For $Co(C_2H_2N_3)_2$: C, 24.63; H, 2.07; N, 43.09. Found: C, 24.48; H, 2.18; N, 43.14. IR (Diamond-ATR, cm^{-1}): 3146 (w), 1722 (w), 1652 (w), 1464 (w), 1421 (w), 1240 (w), 1216 (w), 1111 (s), 998 (w), 978 (s), 795 (s), 723 (w).

Cu(1,2,3-triazolate)$_2$

Cu(1,2,3-triazolate)$_2$ was prepared according to a reported procedure.[11] The as-synthesized Cu(1,2,3-triazolate)$_2$ was soaked in 10 mL of dry and degassed DMF for three times and successively 10 mL of dry and degassed DCM for three times, and was evacuated under vacuum at 100 °C for 2 h. Elemental analysis calcd. For $Cu(C_2H_2N_3)_2(CH_2Cl_2)_{0.15}$: C, 23.47; H, 2.04; N, 39.57. Found: C, 23.16; H, 1.98; N, 39.58. IR (Diamond-ATR, cm^{-1}): 3146 (w), 1650 (w), 1617 (w), 1444 (w), 1424 (w), 1295 (w), 1241 (w), 1220 (w), 1193 (w), 1111 (w), 999 (w), 978 (s), 796 (s), 717 (w).

Zn(1,2,3-triazolate)$_2$

Zn(1,2,3-triazolate)$_2$ was prepared according to a reported procedure.[11] The as-synthesized Zn(1,2,3-triazolate)$_2$ was soaked in 10 mL of dry and degassed DMF for three times and

successively 10 mL of dry and degassed DCM for three times, and was evacuated under vacuum at 100 °C for 2 h. Elemental analysis calcd. For $Zn(C_2H_2N_3)_2$: C, 23.84; H, 2.00; N, 41.71. Found: C, 24.05; H, 1.86; N, 41.61. IR (Diamond-ATR, cm^{-1}): 3145 (w), 1723 (w), 1654 (w), 1603 (w), 1461 (w), 1423 (w), 1298 (w), 1236 (w), 1213 (w), 1189 (w), 1107 (s), 996 (w), 976 (s), 796 (s), 721 (w).

Cd(1,2,3-triazolate)$_2$

Cd(1,2,3-triazolate)$_2$ was prepared according to a reported procedure.[20] The as-synthesized Cd(1,2,3-triazolate)$_2$ was soaked in 10 mL of dry and degassed DMF for three times and successively 10 mL of dry and degassed DCM for three times, and was evacuated under vacuum at 100 °C for 2 h. Elemental analysis calcd. For $Cd(C_2H_2N_3)_2$: C, 19.33; H, 1.62; N, 33.82. Found: C, 19.47; H, 1.68; N, 33.82. IR (Diamond-ATR, cm^{-1}): 1651 (w), 1454 (w), 1421 (w), 1203 (w), 1181 (s), 1097 (s), 992 (w), 972 (s), 794 (s), 714 (w).

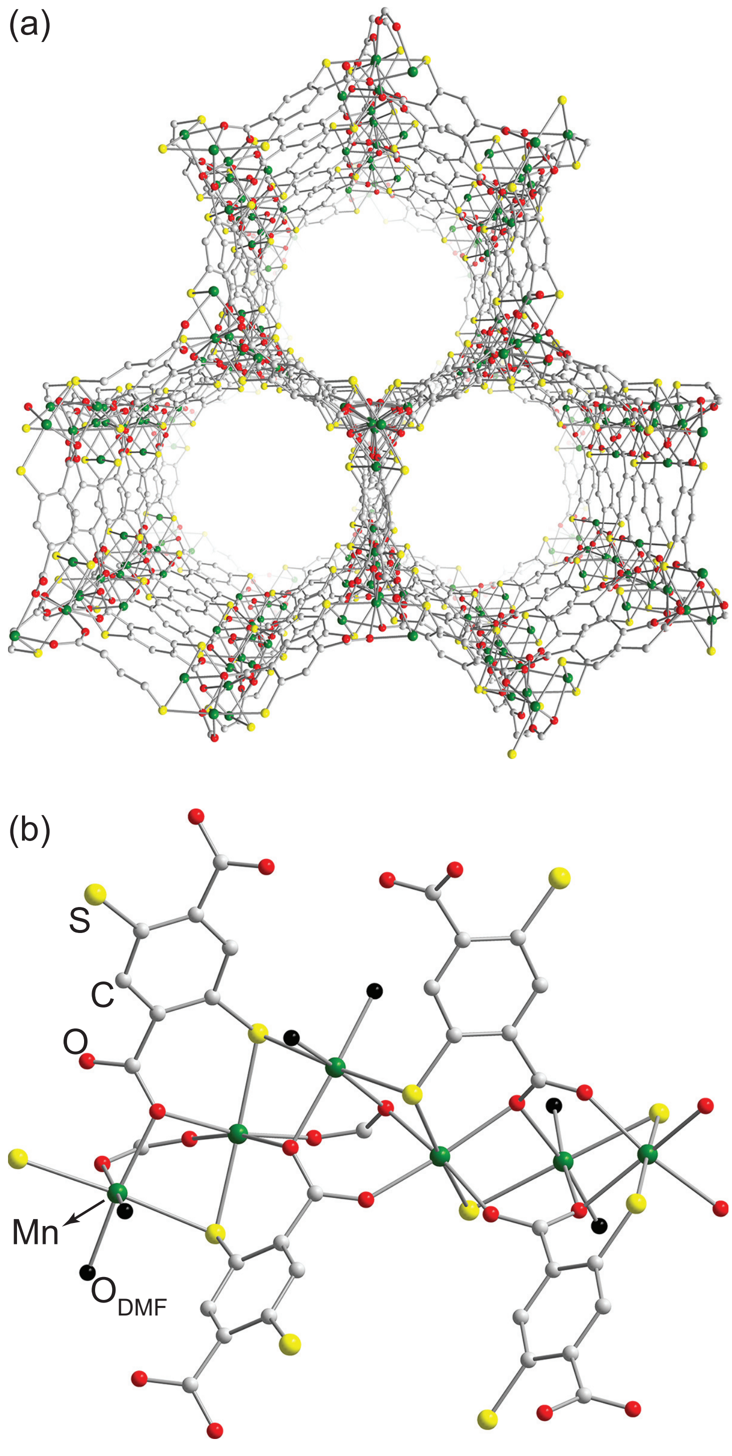

Figure S2. Portions of the crystal structure of $Mn_2(DSBDC)(DMF)_2$. (a) The honeycomb structure of $Mn_2(DSBDC)(DMF)_2$. (b) Coordination environment of Mn centers. Hydrogen atoms and part of DMF molecules have been omitted for clarity.

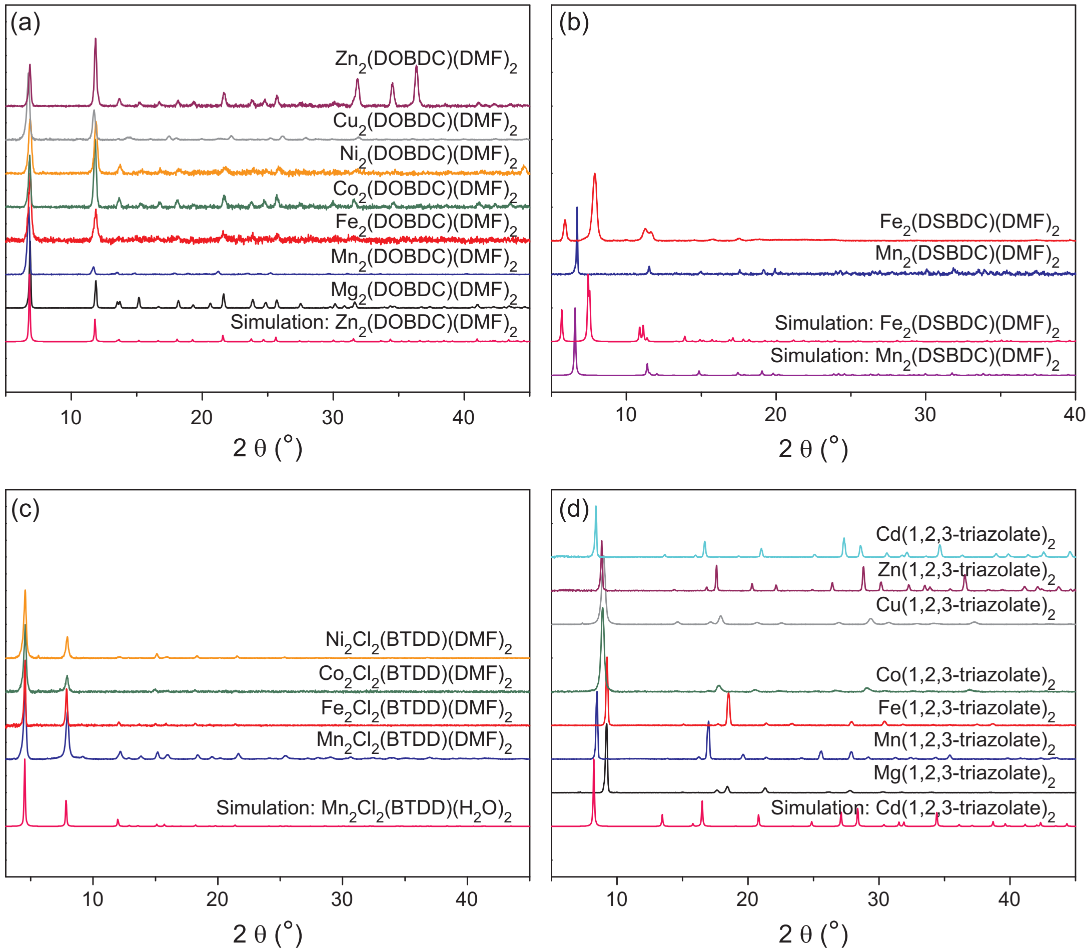

Figure S3. PXRD patterns of the powder samples of (a) M_2(DOBDC)$(DMF)_2$, (b) M_2(DSBDC)$(DMF)_2$, (c) M_2Cl_2(BTDD)$(DMF)_2$, and (d) M(1,2,3-triazolate)$_2$. All materials were desolvated.

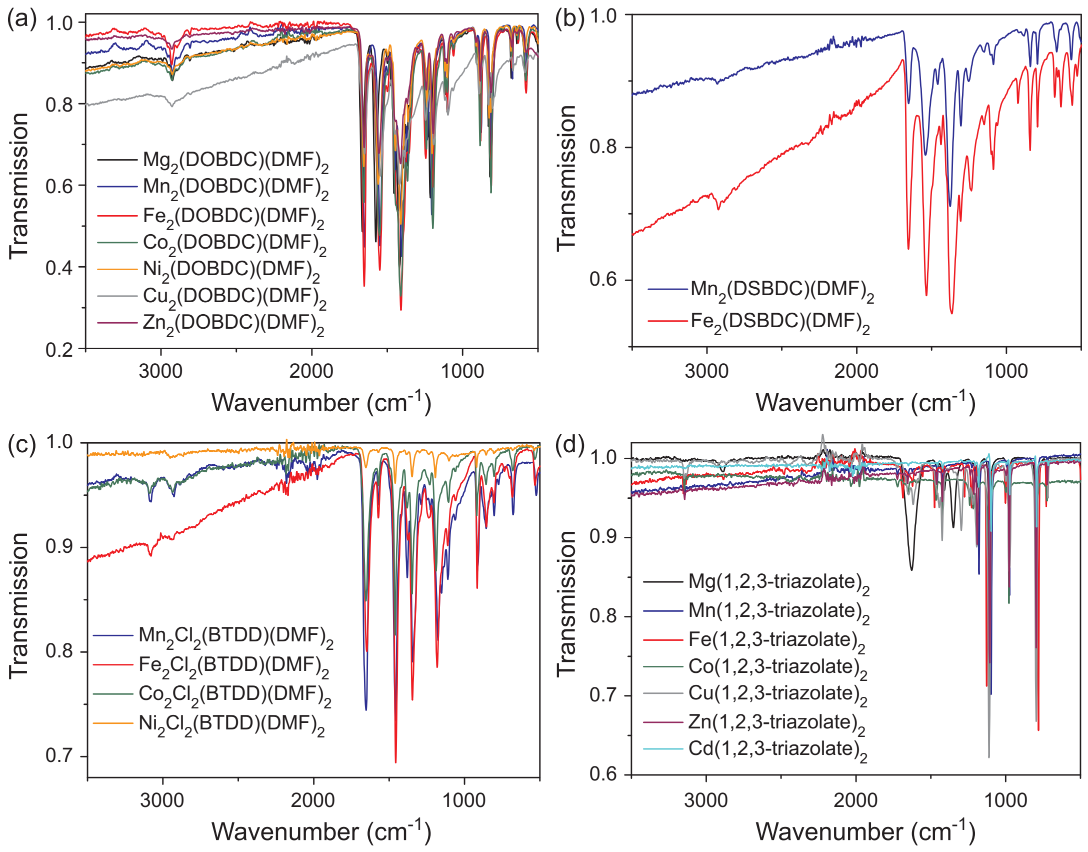

Figure S4. IR spectra of (a) $M_2(DOBDC)(DMF)_2$, (b) $M_2(DSBDC)(DMF)_2$, (c) $M_2Cl_2(BTDD)(DMF)_2$, and (d) M(1,2,3-triazolate)$_2$.

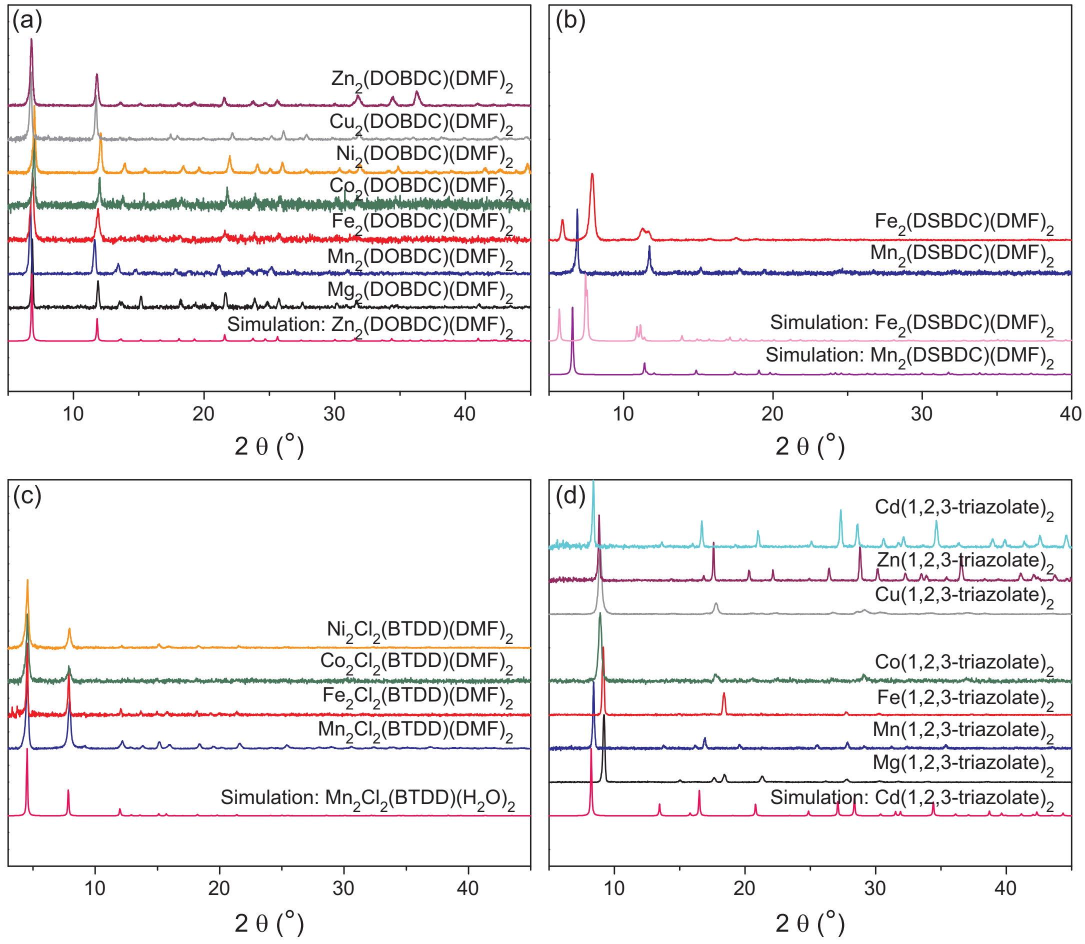

Figure S5. PXRD patterns of the pressed pellets of (a) $M_2(DOBDC)(DMF)_2$, (b) $M_2(DSBDC)(DMF)_2$, (c) $M_2Cl_2(BTDD)(DMF)_2$, and (d) $M(1,2,3\text{-triazolate})_2$.

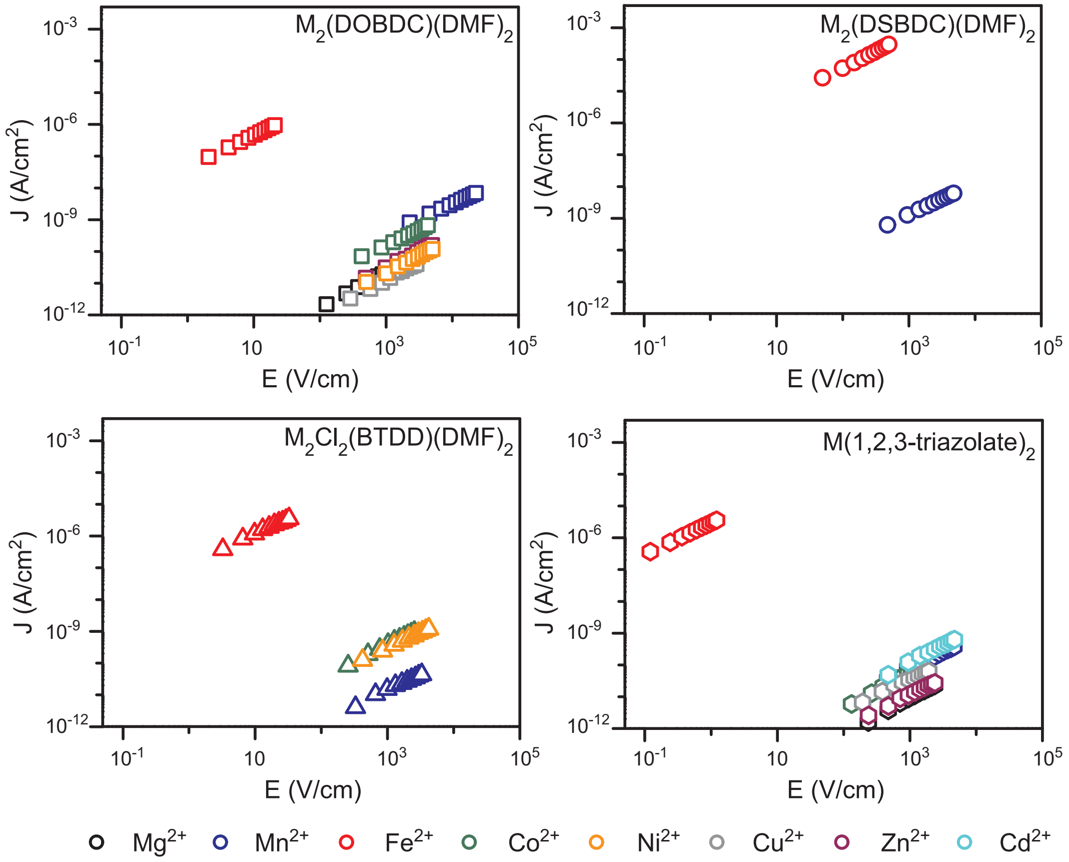

Figure S6. Plots of current density (J) versus electrical field strength (E) for $M_2(DOBDC)(DMF)_2$, $M_2(DSBDC)(DMF)_2$, $M_2Cl_2(BTDD)(DMF)_2$, and $M(1,2,3\text{-triazolate})_2$. The colors of various metal ions are specified at the bottom.

Table S1. Electrical conductivity (σ) of $M_2(DOBDC)(DMF)_2$, $M_2(DSBDC)(DMF)_2$, $M_2Cl_2(BTDD)(DMF)_2$, and $M(1,2,3\text{-triazolate})_2$ measured at 300 K, in N_2 atmosphere, and in the dark.

Metal ion	σ [M_2(DOBDC)($DMF)_2$] (S/cm)	σ [M_2(DSBDC)($DMF)_2$] (S/cm)	σ [M_2Cl_2(BTDD)($DMF)_2$] (S/cm)	σ [M(1,2,3-triazolate$)_2$] (S/cm)
Mg^{2+}	2.1×10^{-14}			9.9×10^{-15}
Mn^{2+}	3.0×10^{-13}	1.2×10^{-12}	1.3×10^{-14}	8.2×10^{-14}
Fe^{2+}	4.8×10^{-8}	5.8×10^{-7}	1.1×10^{-7}	3.0×10^{-6}
Co^{2+}	1.5×10^{-13}		3.7×10^{-13}	5.1×10^{-14}
Ni^{2+}	2.8×10^{-14}		2.8×10^{-13}	
Cu^{2+}	1.4×10^{-14}			3.2×10^{-14}
Zn^{2+}	3.3×10^{-14}			1.2×10^{-14}
Cd^{2+}				1.4×10^{-13}

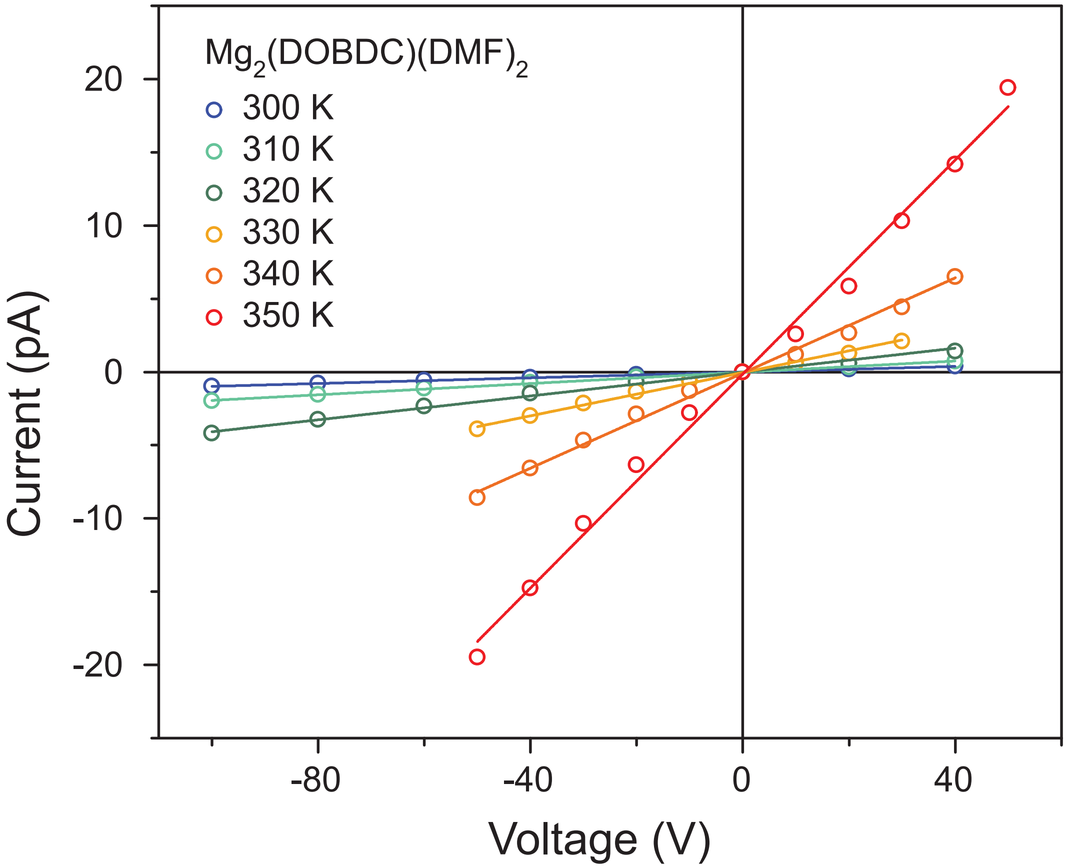

Figure S7. I-V curves of $Mg_2(DOBDC)(DMF)_2$ at various temperatures. Circles represent experimental data, and lines are linear regression fitting curves.

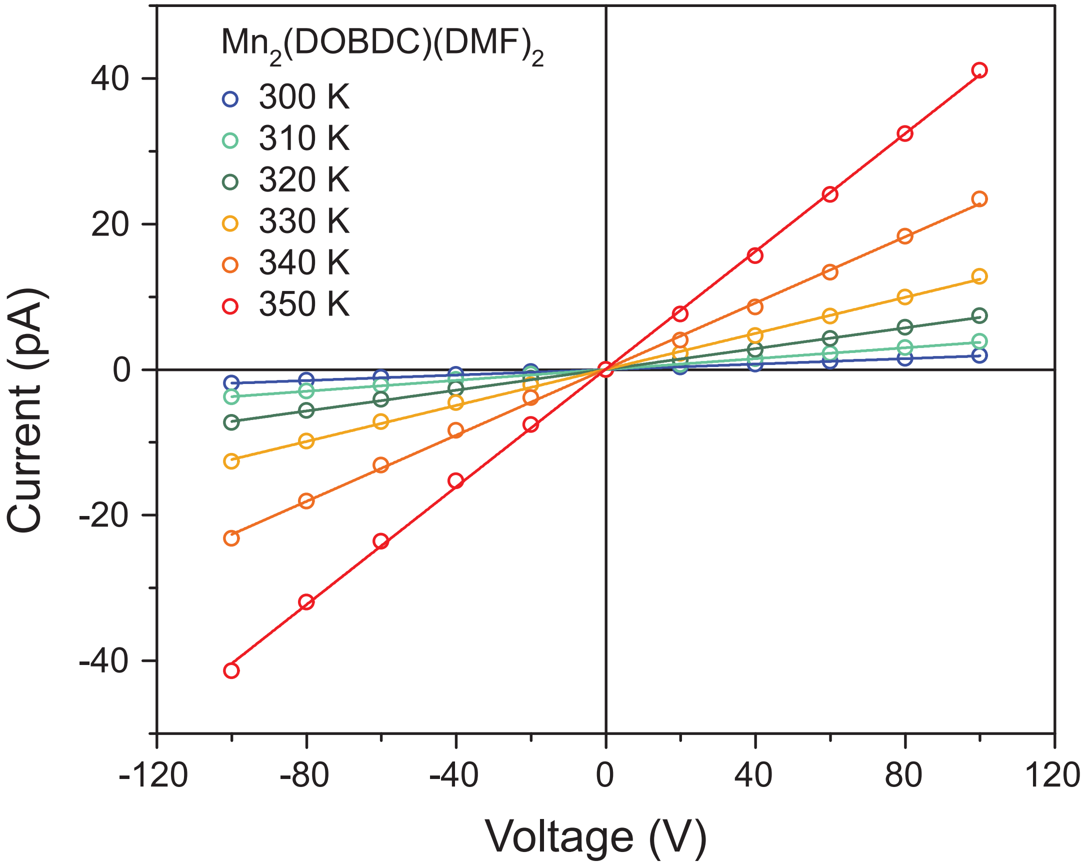

Figure S8. I-V curves of $Mn_2(DOBDC)(DMF)_2$ at various temperatures. Circles represent experimental data, and lines are linear regression fitting curves.

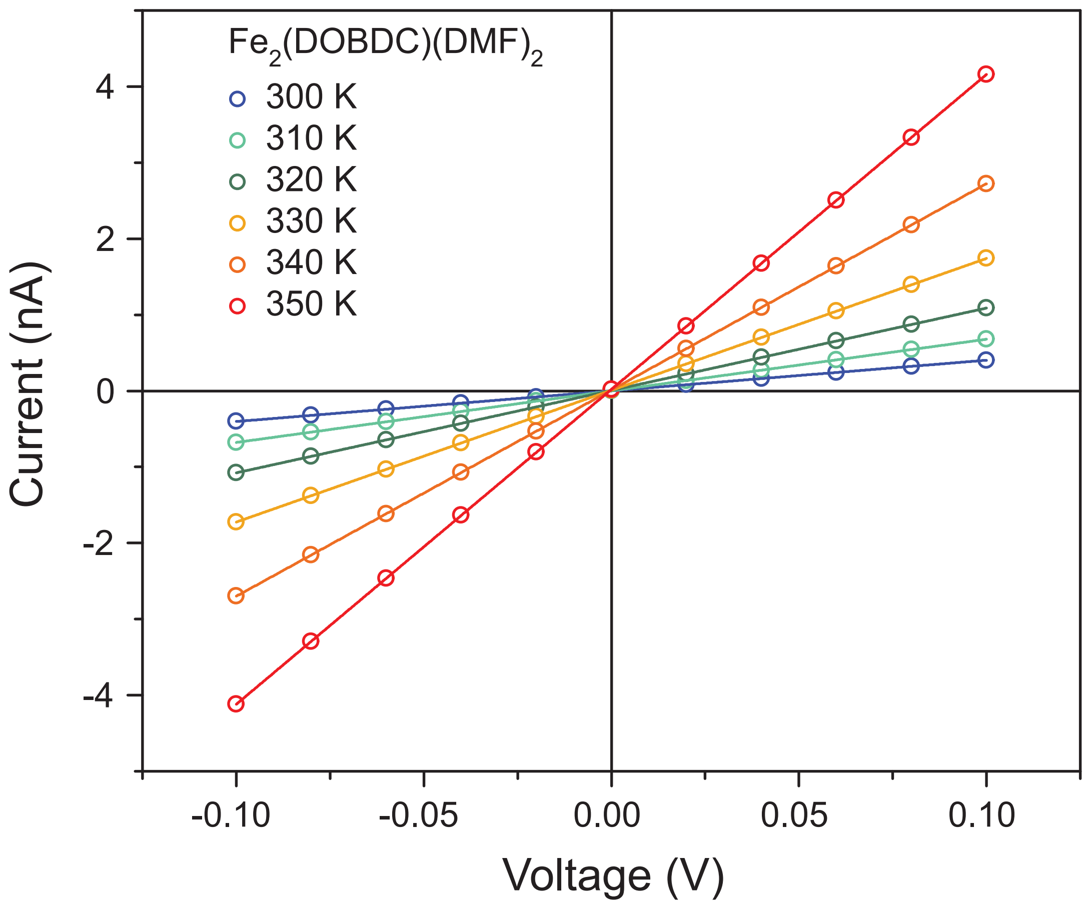

Figure S9. I-V curves of $Fe_2(DOBDC)(DMF)_2$ at various temperatures. Circles represent experimental data, and lines are linear regression fitting curves.

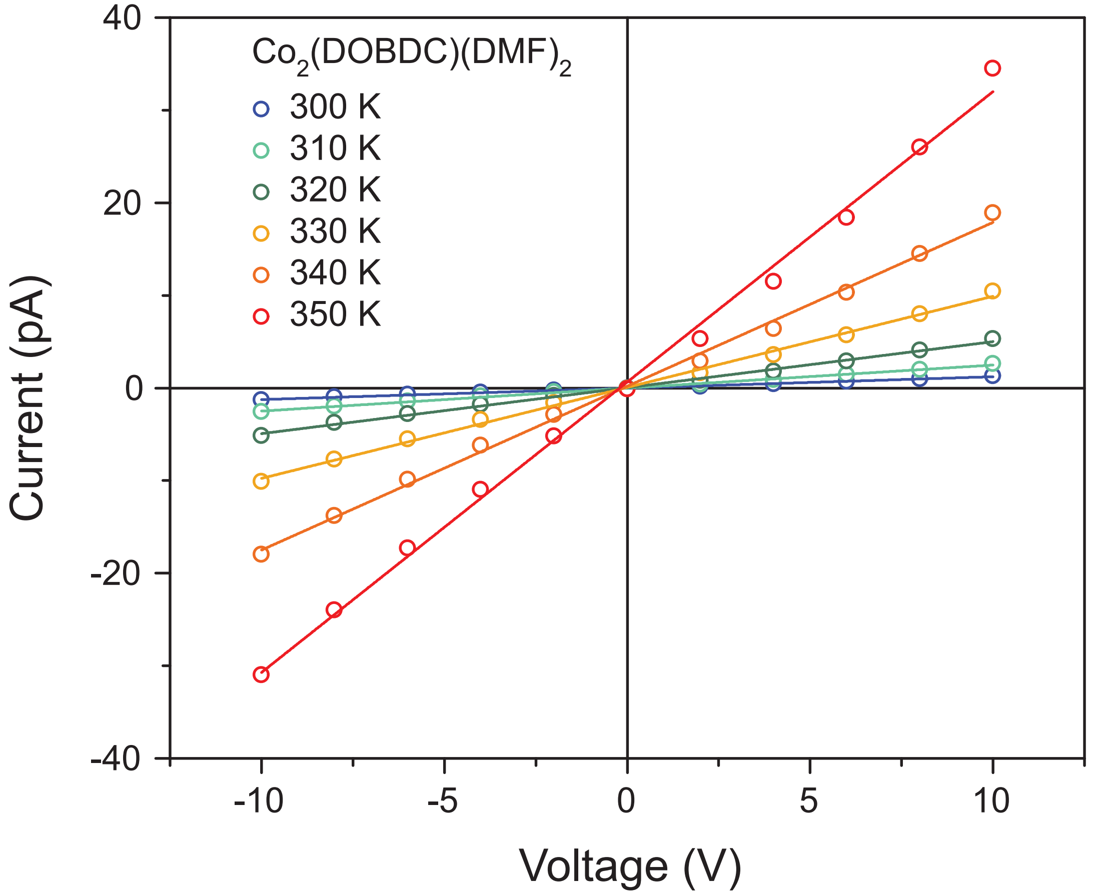

Figure S10. I-V curves of $Co_2(DOBDC)(DMF)_2$ at various temperatures. Circles represent experimental data, and lines are linear regression fitting curves.

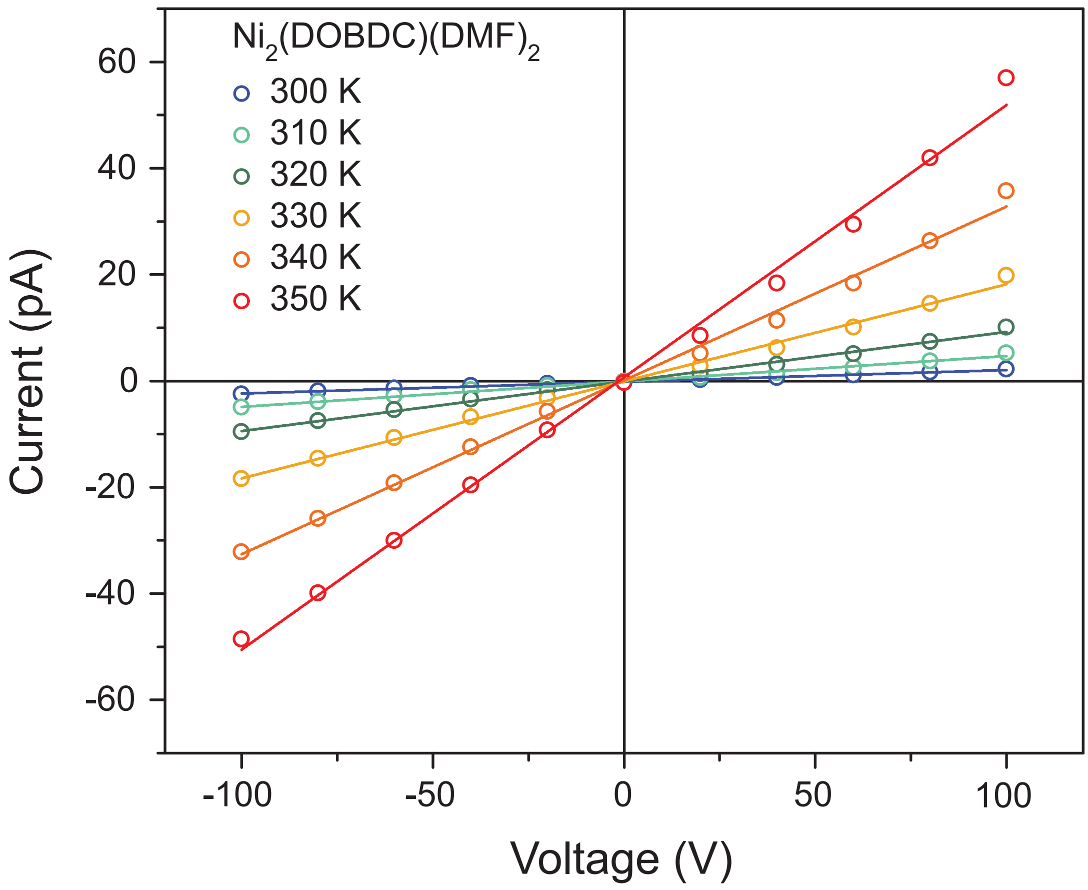

Figure S11. I-V curves of $Ni_2(DOBDC)(DMF)_2$ at various temperatures. Circles represent experimental data, and lines are linear regression fitting curves.

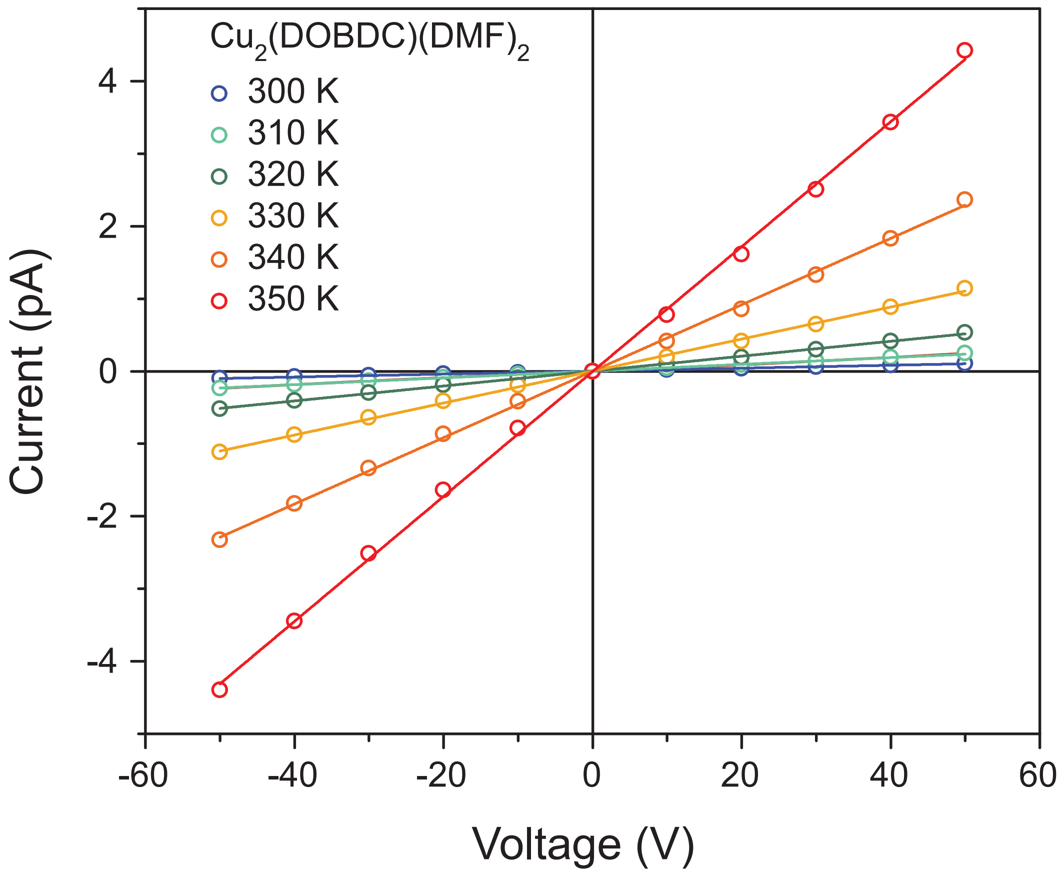

Figure S12. I-V curves of $Cu_2(DOBDC)(DMF)_2$ at various temperatures. Circles represent experimental data, and lines are linear regression fitting curves.

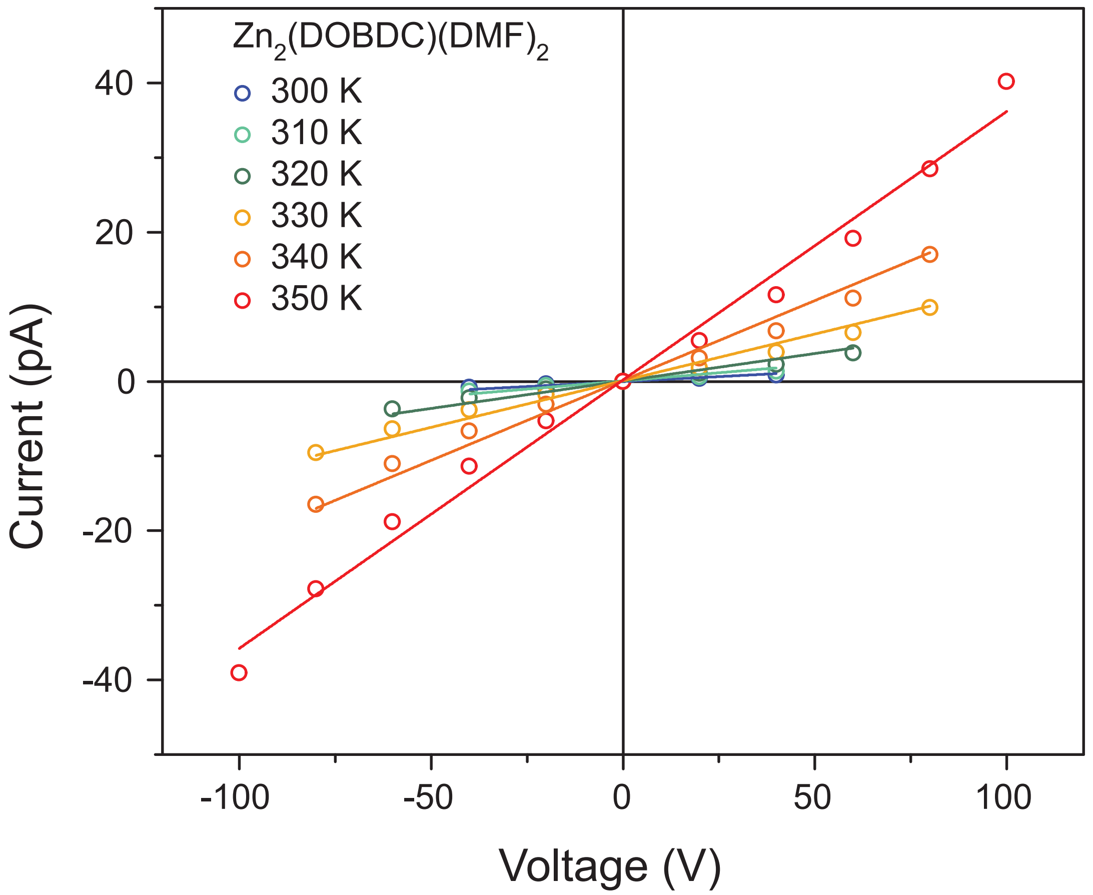

Figure S13. I-V curves of $Zn_2(DOBDC)(DMF)_2$ at various temperatures. Circles represent experimental data, and lines are linear regression fitting curves.

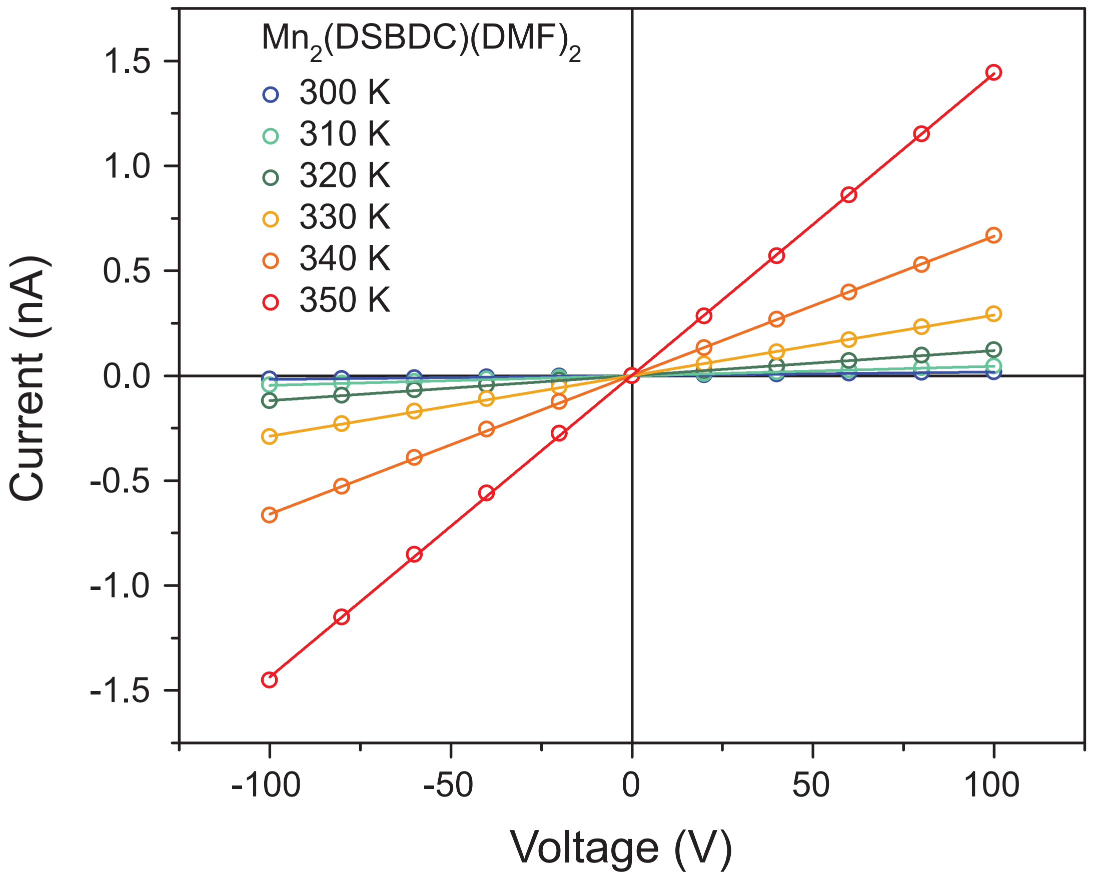

Figure S14. I-V curves of $Mn_2(DSBDC)(DMF)_2$ at various temperatures. Circles represent experimental data, and lines are linear regression fitting curves.

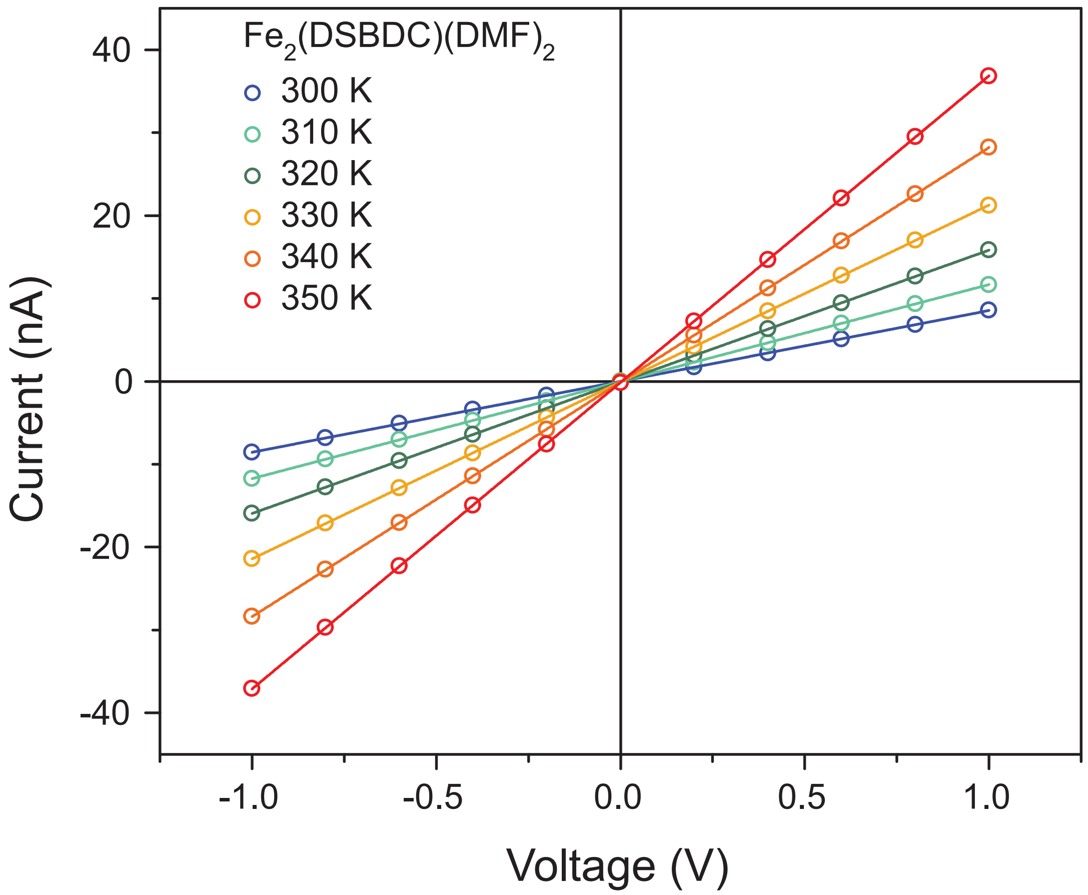

Figure S15. I-V curves of $Fe_2(DSBDC)(DMF)_2$ at various temperatures. Circles represent experimental data, and lines are linear regression fitting curves.

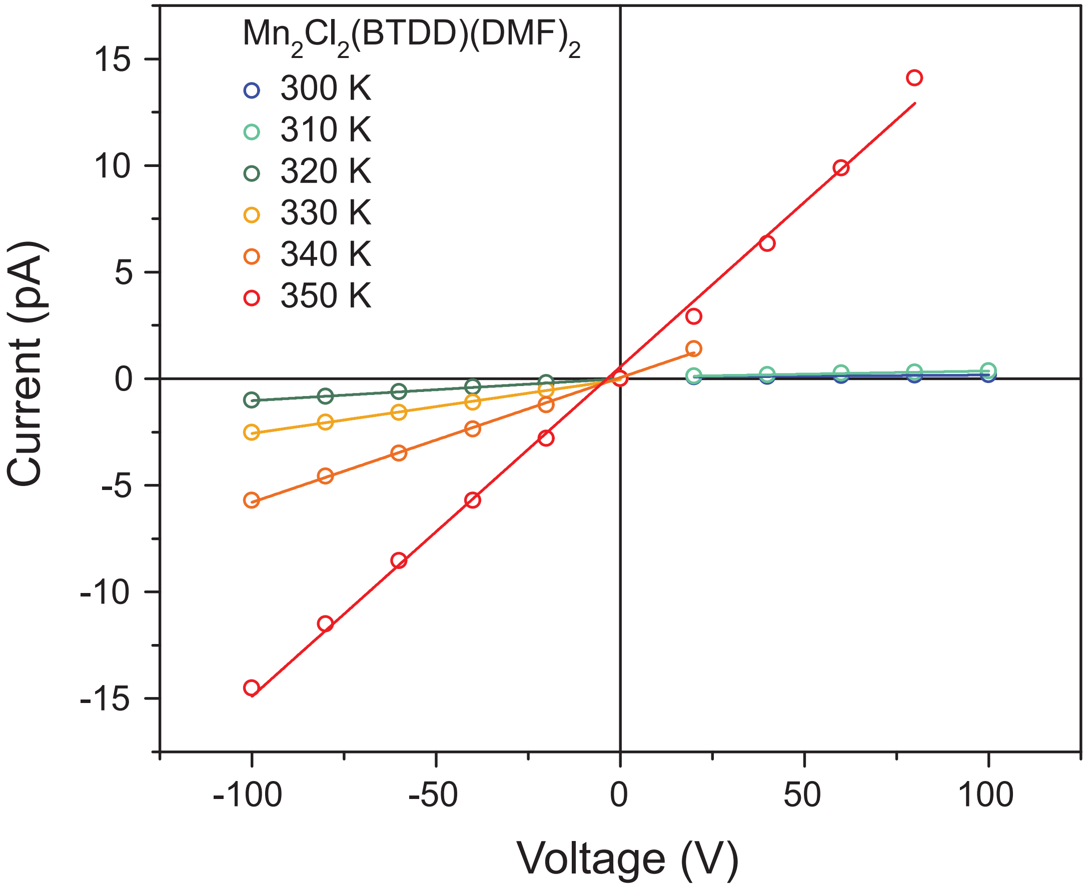

Figure S16. I-V curves of $Mn_2Cl_2(BTDD)(DMF)_2$ at various temperatures. Circles represent experimental data, and lines are linear regression fitting curves.

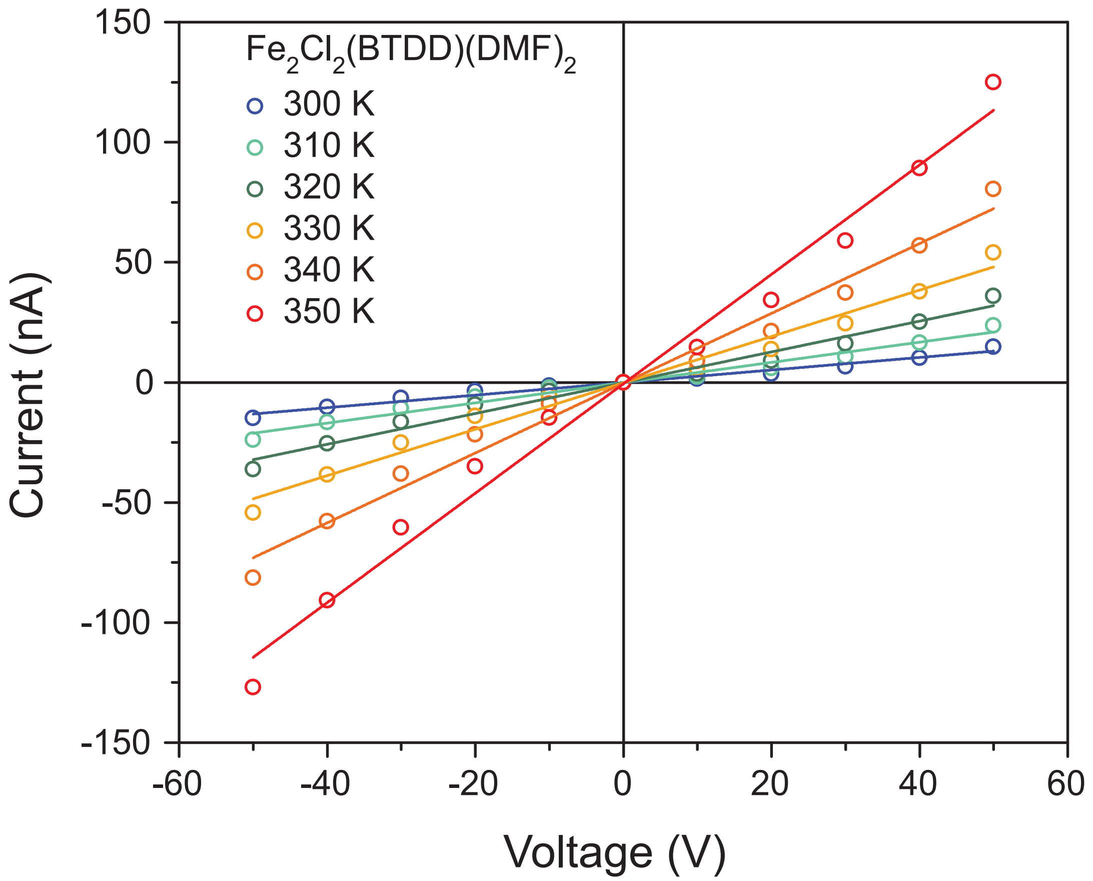

Figure S17. I-V curves of Fe_2Cl_2(BTDD)$(DMF)_2$ at various temperatures. Circles represent experimental data, and lines are linear regression fitting curves.

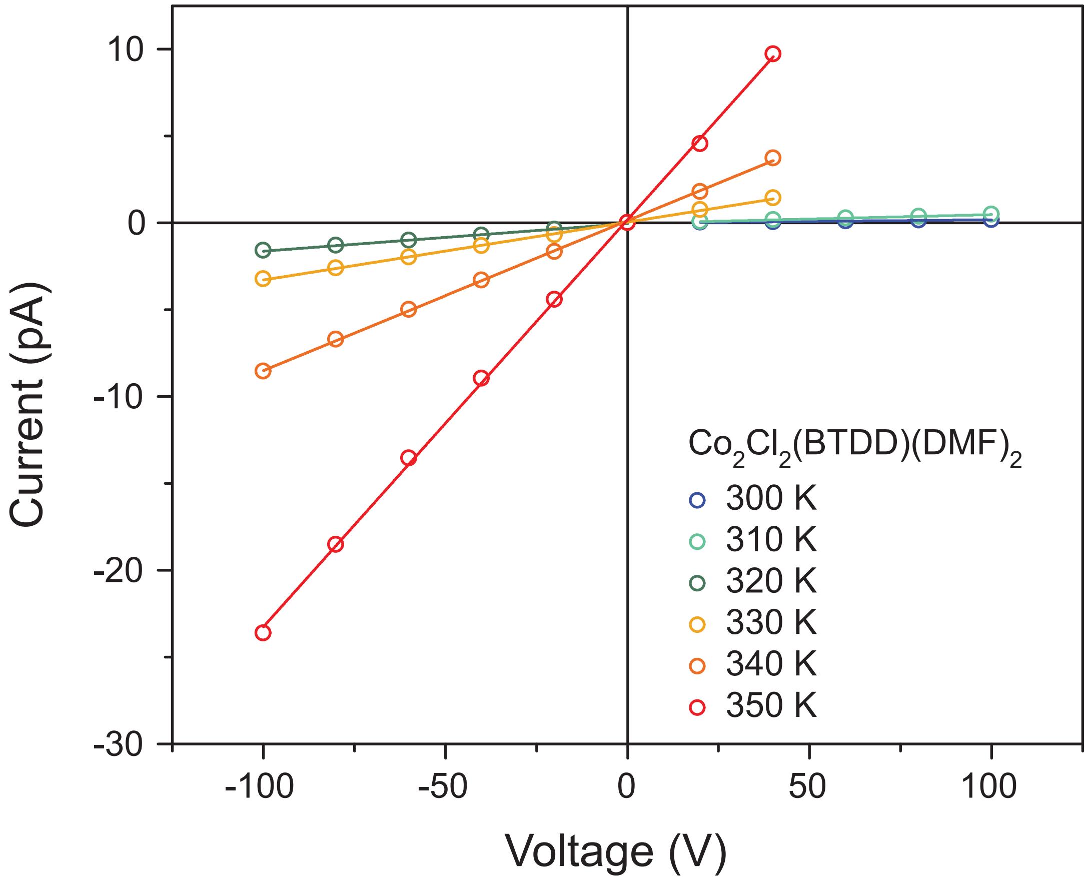

Figure S18. I-V curves of $Co_2Cl_2(BTDD)(DMF)_2$ at various temperatures. Circles represent experimental data, and lines are linear regression fitting curves.

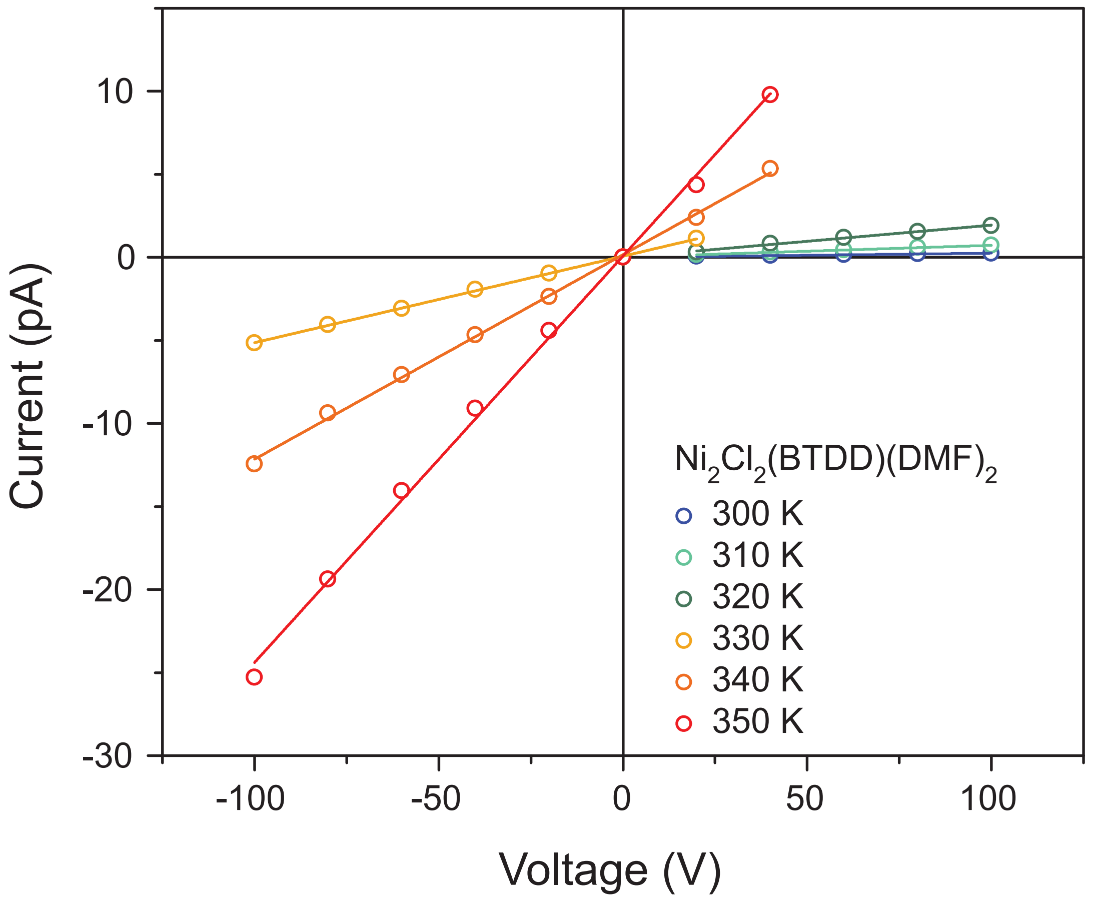

Figure S19. I-V curves of $Ni_2Cl_2(BTDD)(DMF)_2$ at various temperatures. Circles represent experimental data, and lines are linear regression fitting curves.

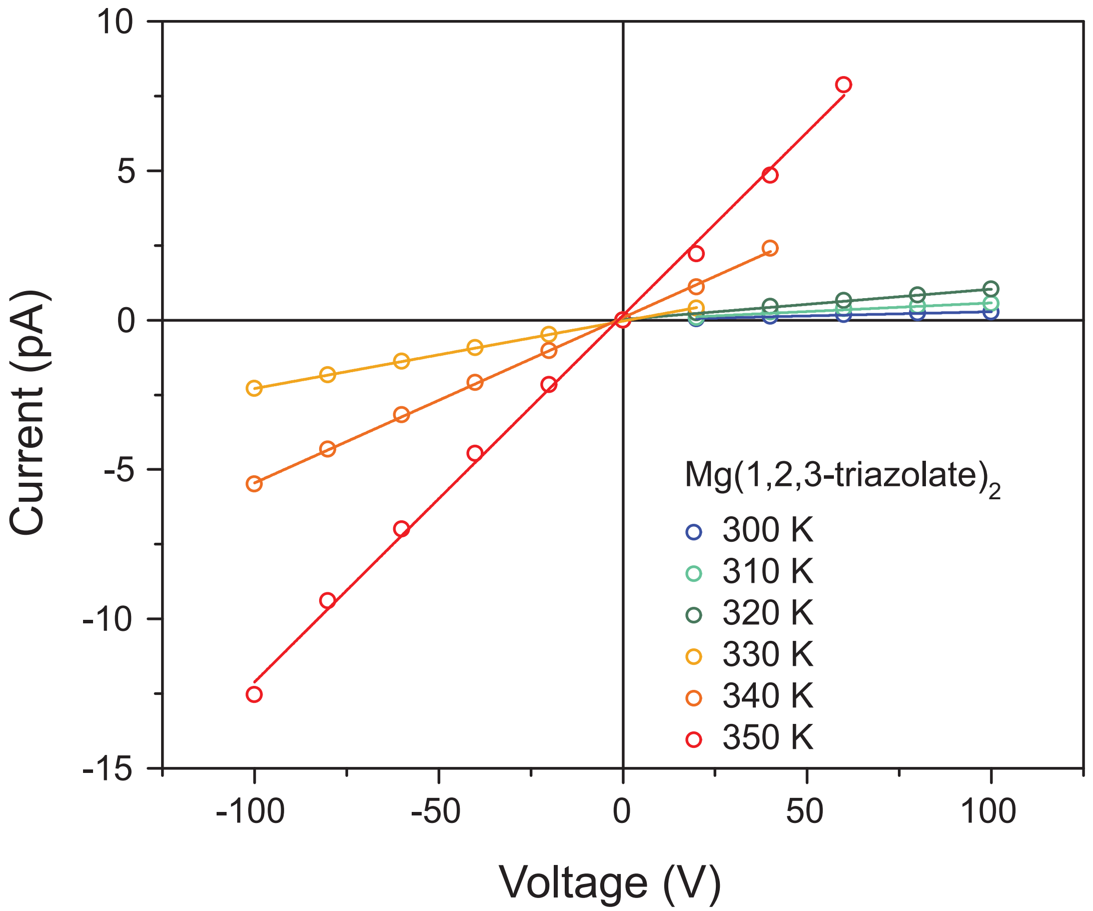

Figure S20. I-V curves of Mg(1,2,3-triazolate)$_2$ at various temperatures. Circles represent experimental data, and lines are linear regression fitting curves.

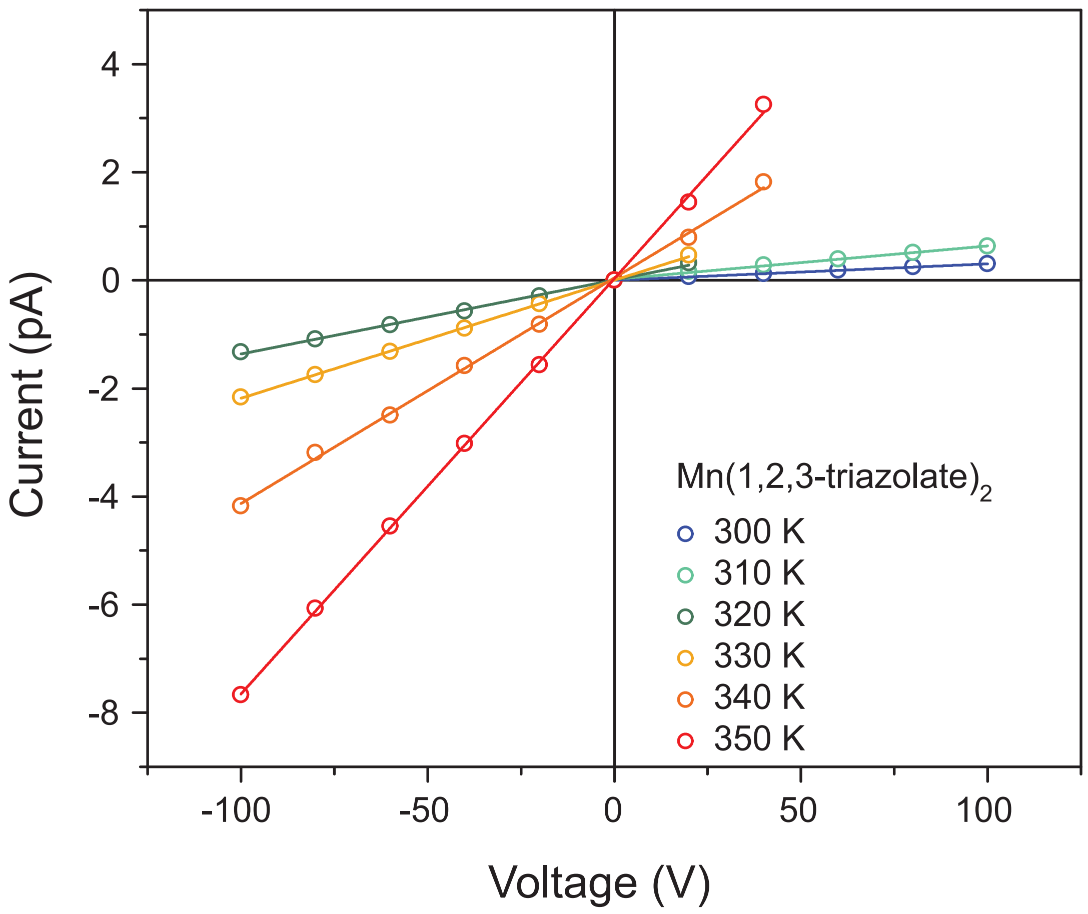

Figure S21. I-V curves of Mn(1,2,3-triazolate)$_2$ at various temperatures. Circles represent experimental data, and lines are linear regression fitting curves.

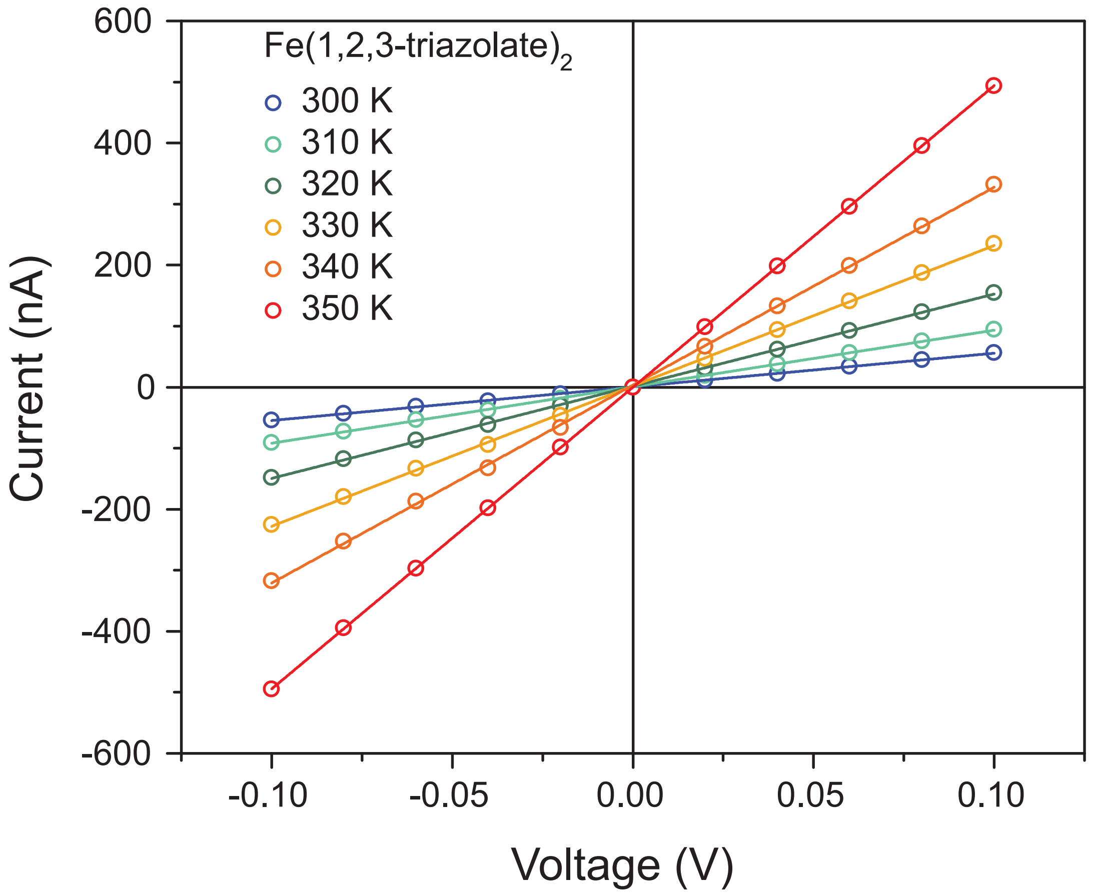

Figure S22. I-V curves of Fe(1,2,3-triazolate)$_2$ at various temperatures. Circles represent experimental data, and lines are linear regression fitting curves.

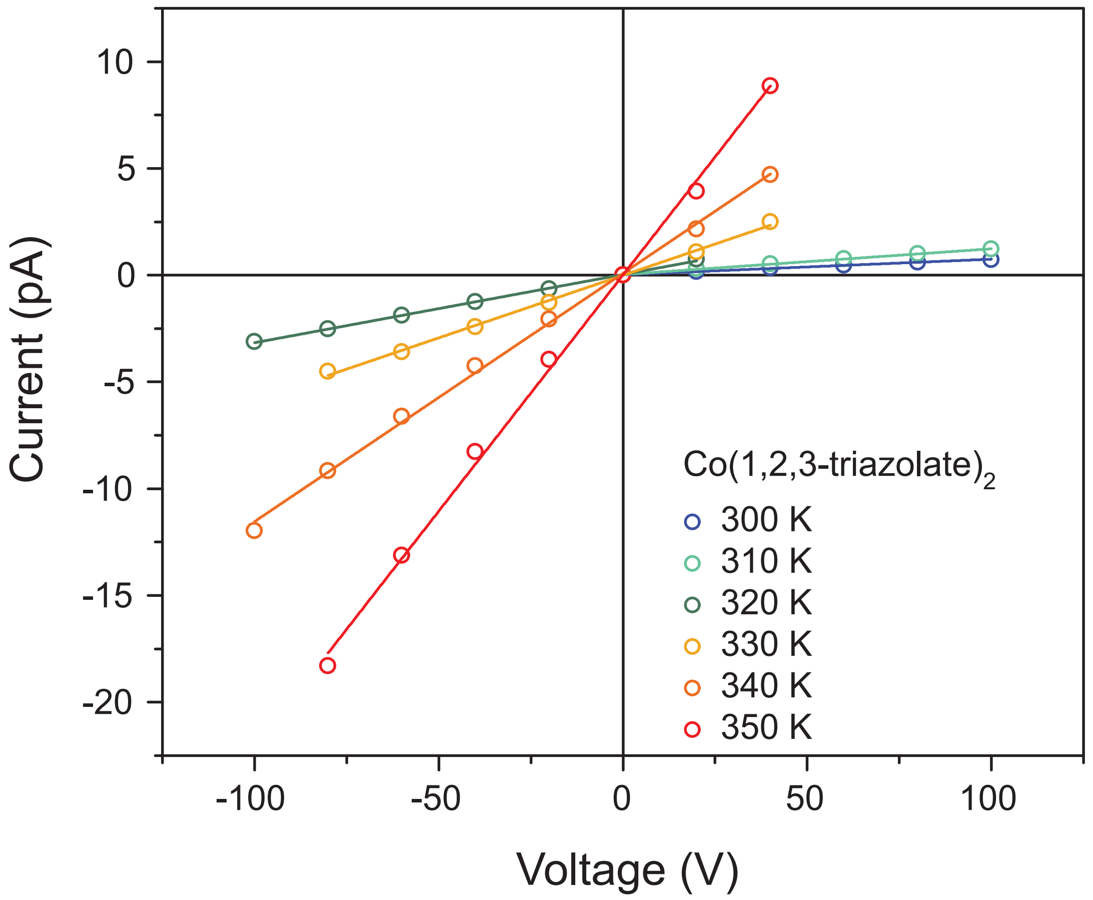

Figure S23. I-V curves of Co(1,2,3-triazolate)$_2$ at various temperatures. Circles represent experimental data, and lines are linear regression fitting curves.

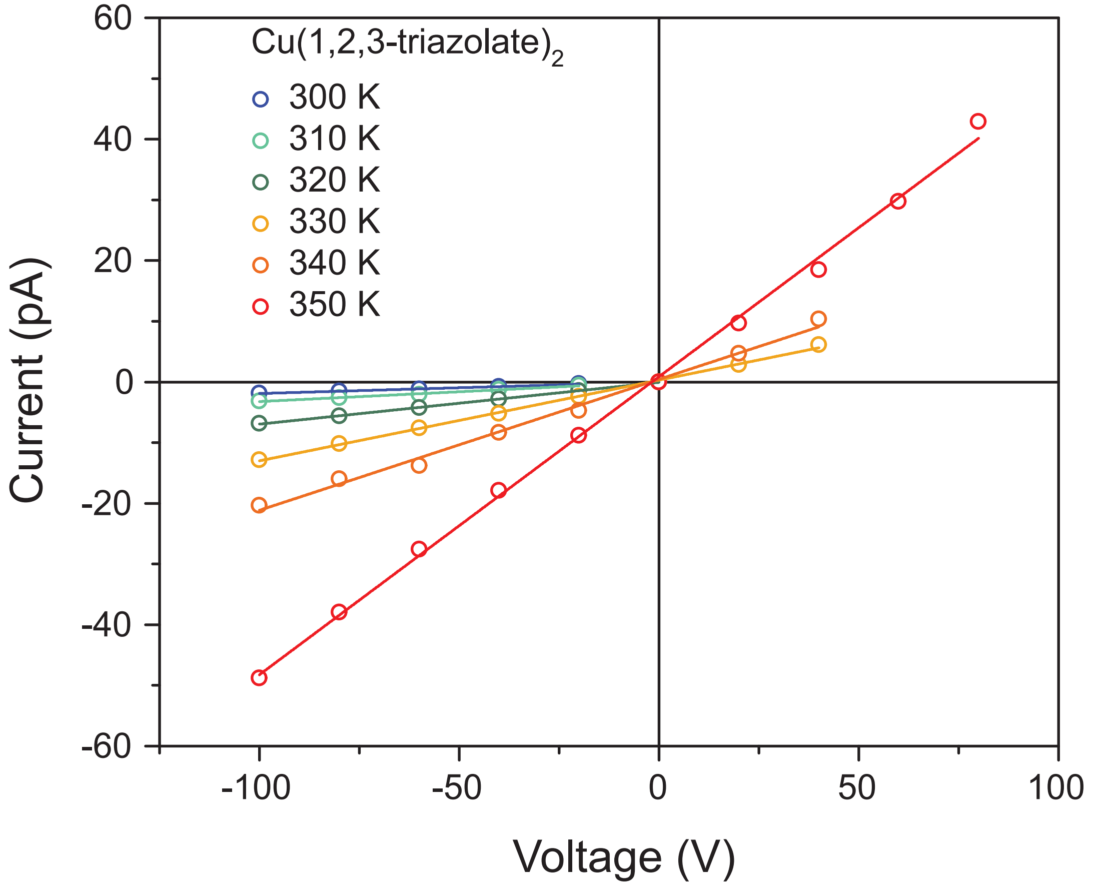

Figure S24. I-V curves of Cu(1,2,3-triazolate)$_2$ at various temperatures. Circles represent experimental data, and lines are linear regression fitting curves.

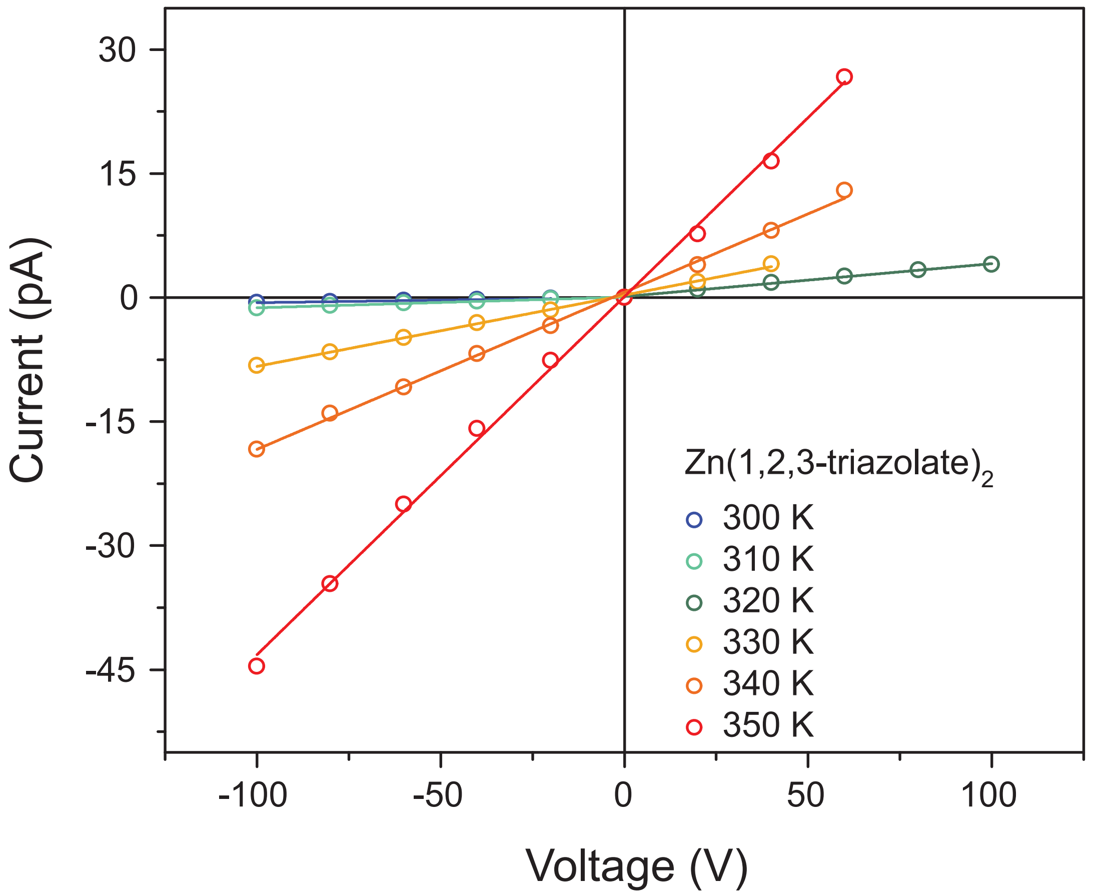

Figure S25. I-V curves of Zn(1,2,3-triazolate)$_2$ at various temperatures. Circles represent experimental data, and lines are linear regression fitting curves.

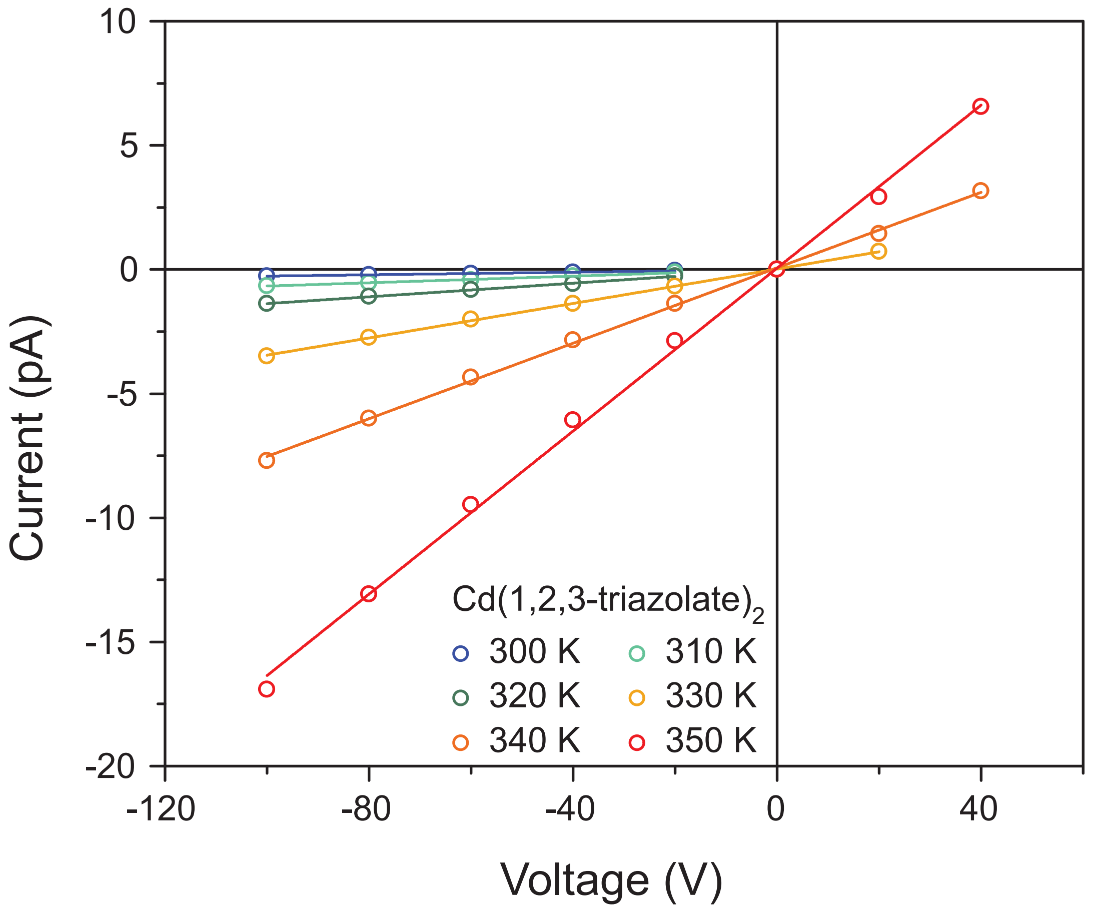

Figure S26. I-V curves of Cd(1,2,3-triazolate)$_2$ at various temperatures. Circles represent experimental data, and lines are linear regression fitting curves.

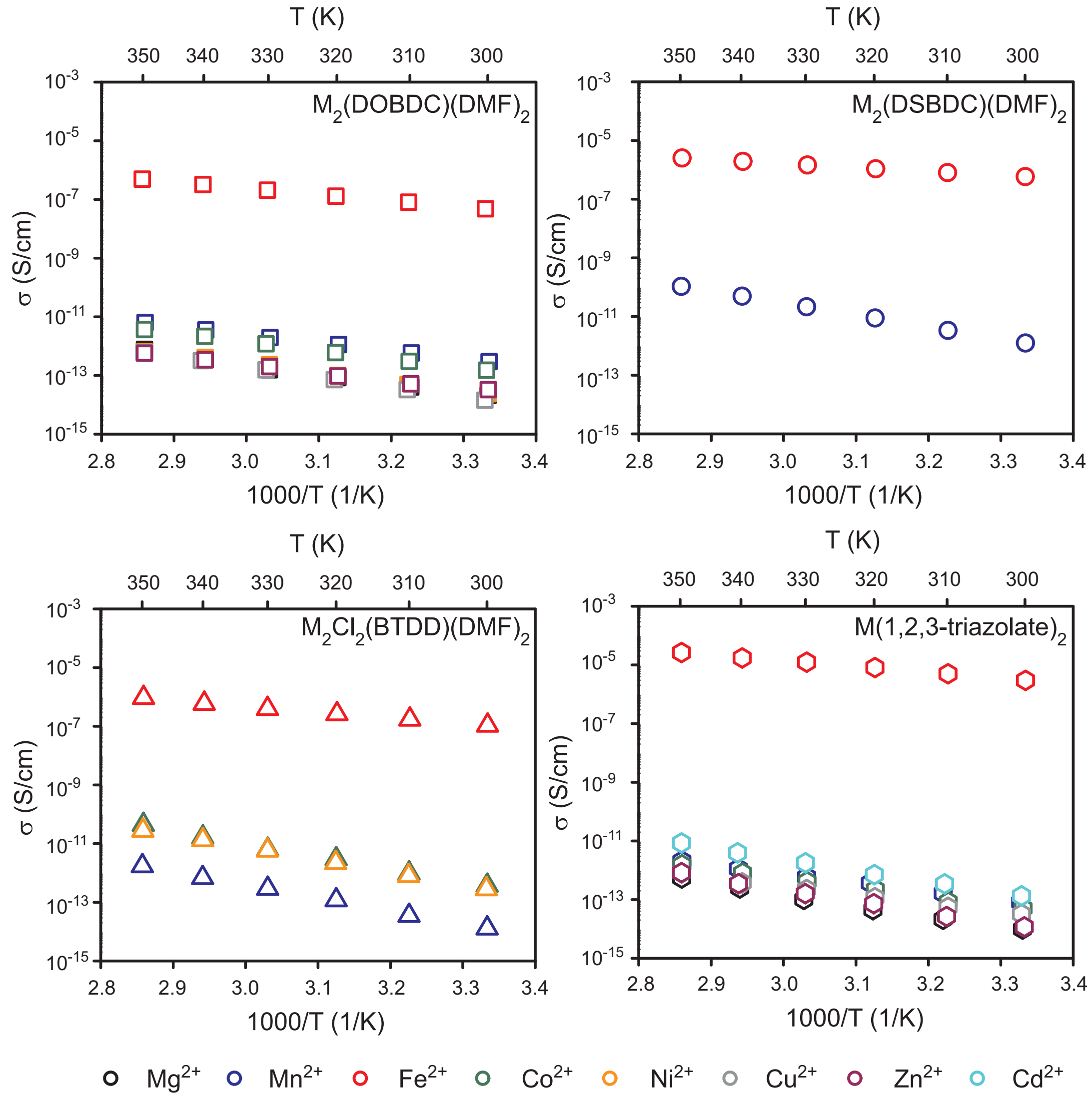

Figure S27. Plots of electrical conductivity (σ) versus temperature (T) for $M_2(DOBDC)(DMF)_2$, $M_2(DSBDC)(DMF)_2$, $M_2Cl_2(BTDD)(DMF)_2$, and M(1,2,3-triazolate)$_2$. The colors of various metal ions are specified at the bottom.

Table S2. Activation energies (E_a) of $M_2(DOBDC)(DMF)_2$, $M_2(DSBDC)(DMF)_2$, $M_2Cl_2(BTDD)(DMF)_2$, and M(1,2,3-triazolate)$_2$ measured at 300 – 350 K, in vacuum, and in the dark.

Metal ion	E_a [$M_2(DOBDC)(DMF)_2$] (eV)	E_a [$M_2(DSBDC)(DMF)_2$] (eV)	E_a [$M_2Cl_2(BTDD)(DMF)_2$] (eV)	E_a [M(1,2,3-triazolate)$_2$] (eV)
Mg^{2+}	0.64			0.74
Mn^{2+}	0.55	0.81	0.89	0.59
Fe^{2+}	0.42	0.27	0.39	0.39
Co^{2+}	0.58		0.86	0.64
Ni^{2+}	0.62		0.84	
Cu^{2+}	0.69			0.59
Zn^{2+}	0.54			0.77
Cd^{2+}				0.75

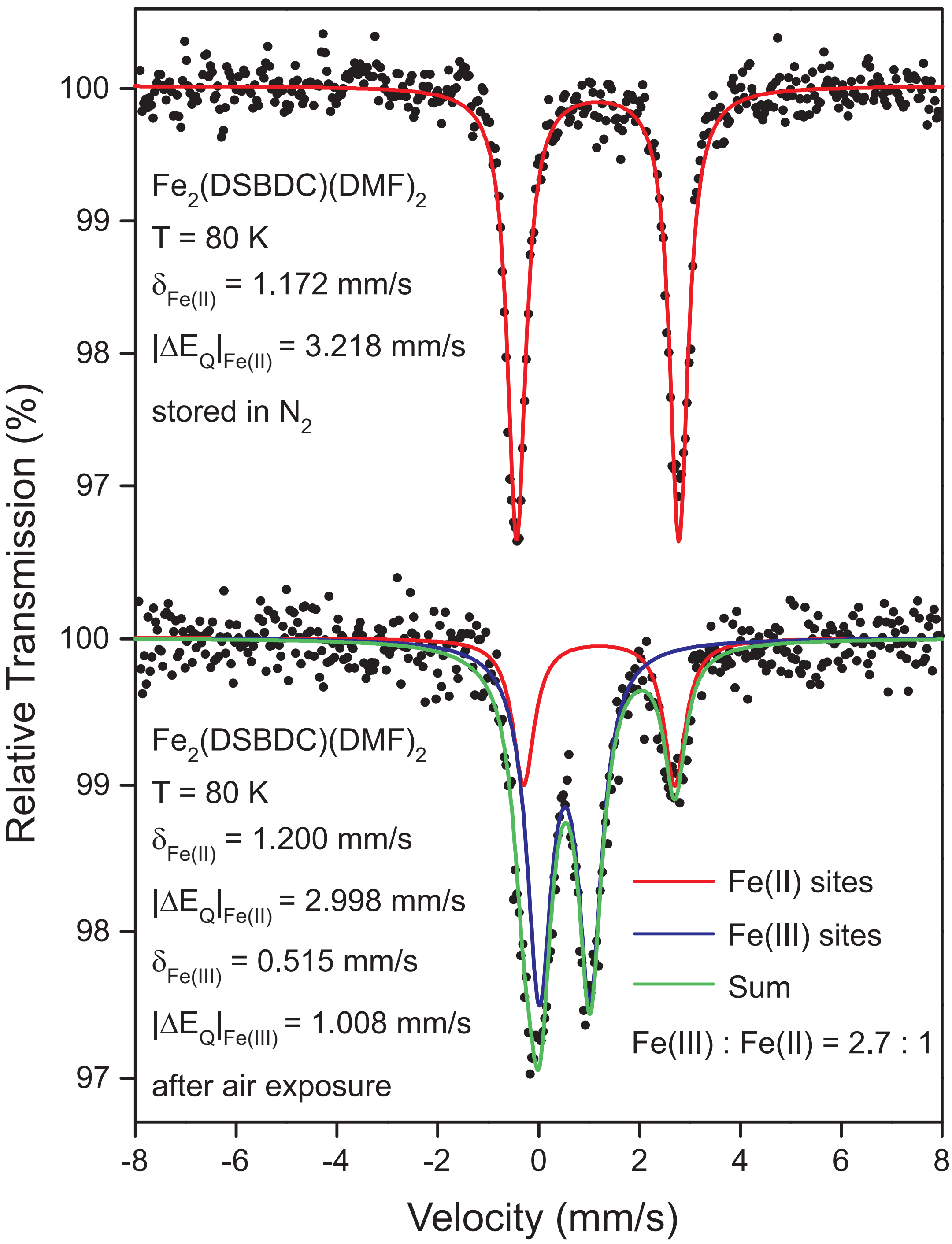

Figure S28. ^{57}Fe Mössbauer spectrum of Fe_2(DSBDC)(DMF)$_2$ that was exposed to air in comparison with the spectrum of Fe_2(DSBDC)(DMF)$_2$ that was stored in N_2 atmosphere. After air exposure for less than 5 seconds, approximately 73% of Fe^{2+} centers were oxidized to Fe^{3+}. Black dots represent experimental data. Red, blue, and green curves represent fitting curves of Fe^{2+} sites, Fe^{3+} sites, and the sum of these two curves, respectively. The isomer shift (δ) and the quadrupole splitting ($|\Delta E_Q|$) of each site as well as the measurement temperature (T) and the ratio between Fe^{3+} and Fe^{2+} sites in the sample are shown in the figure.

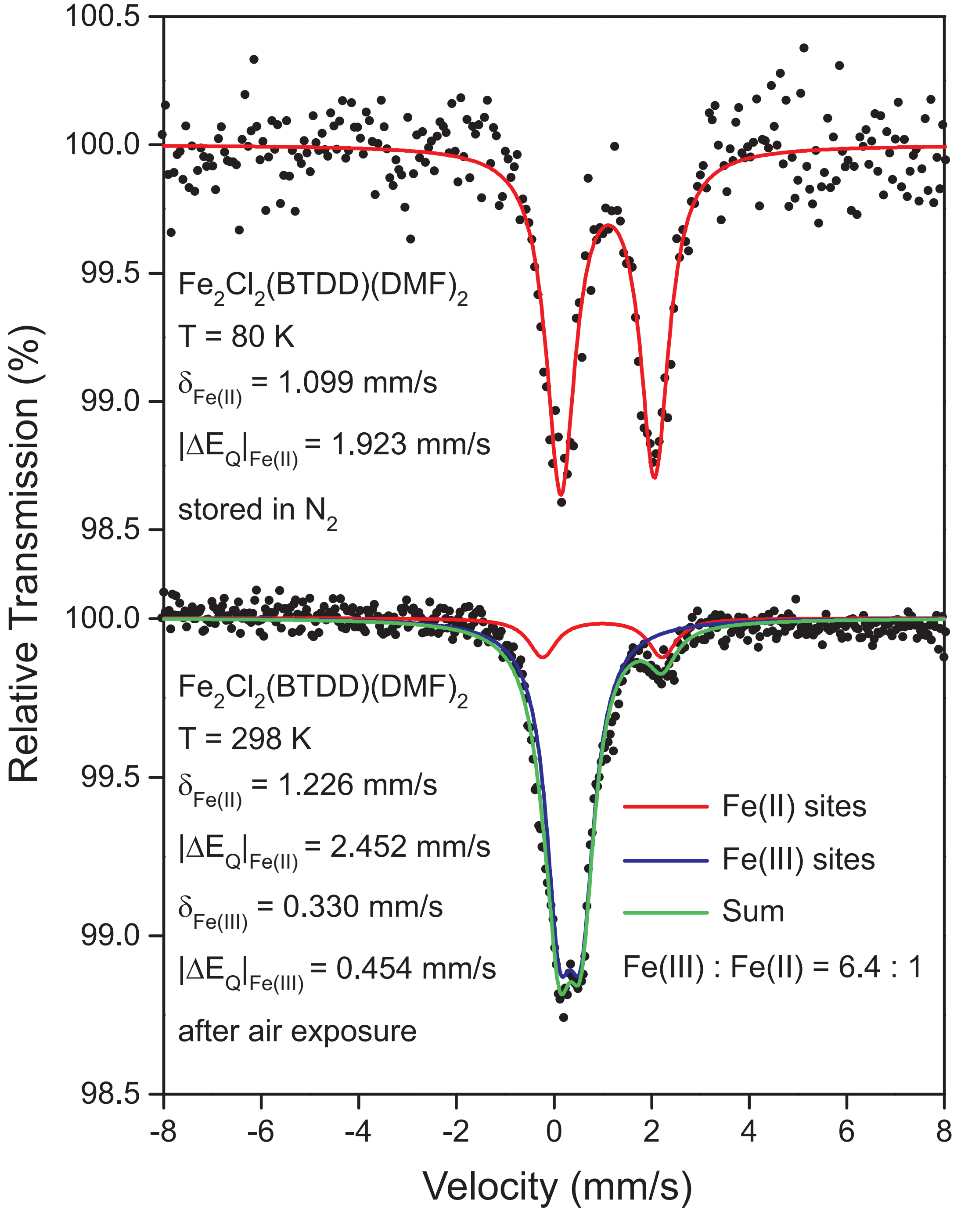

Figure S29. ^{57}Fe Mössbauer spectrum of $Fe_2Cl_2(BTDD)(DMF)_2$ that was exposed to air in comparison with the spectrum of $Fe_2Cl_2(BTDD)(DMF)_2$ that was stored in N_2 atmosphere. After air exposure for less than 5 seconds, approximately 86% of Fe^{2+} centers were oxidized to Fe^{3+}. Black dots represent experimental data. Red, blue, and green curves represent fitting curves of Fe^{2+} sites, Fe^{3+} sites, and the sum of these two curves, respectively. The isomer shift (δ) and the quadrupole splitting ($|\Delta E_Q|$) of each site as well as the measurement temperature (T) and the ratio between Fe^{3+} and Fe^{2+} sites in the sample are shown in the figure.

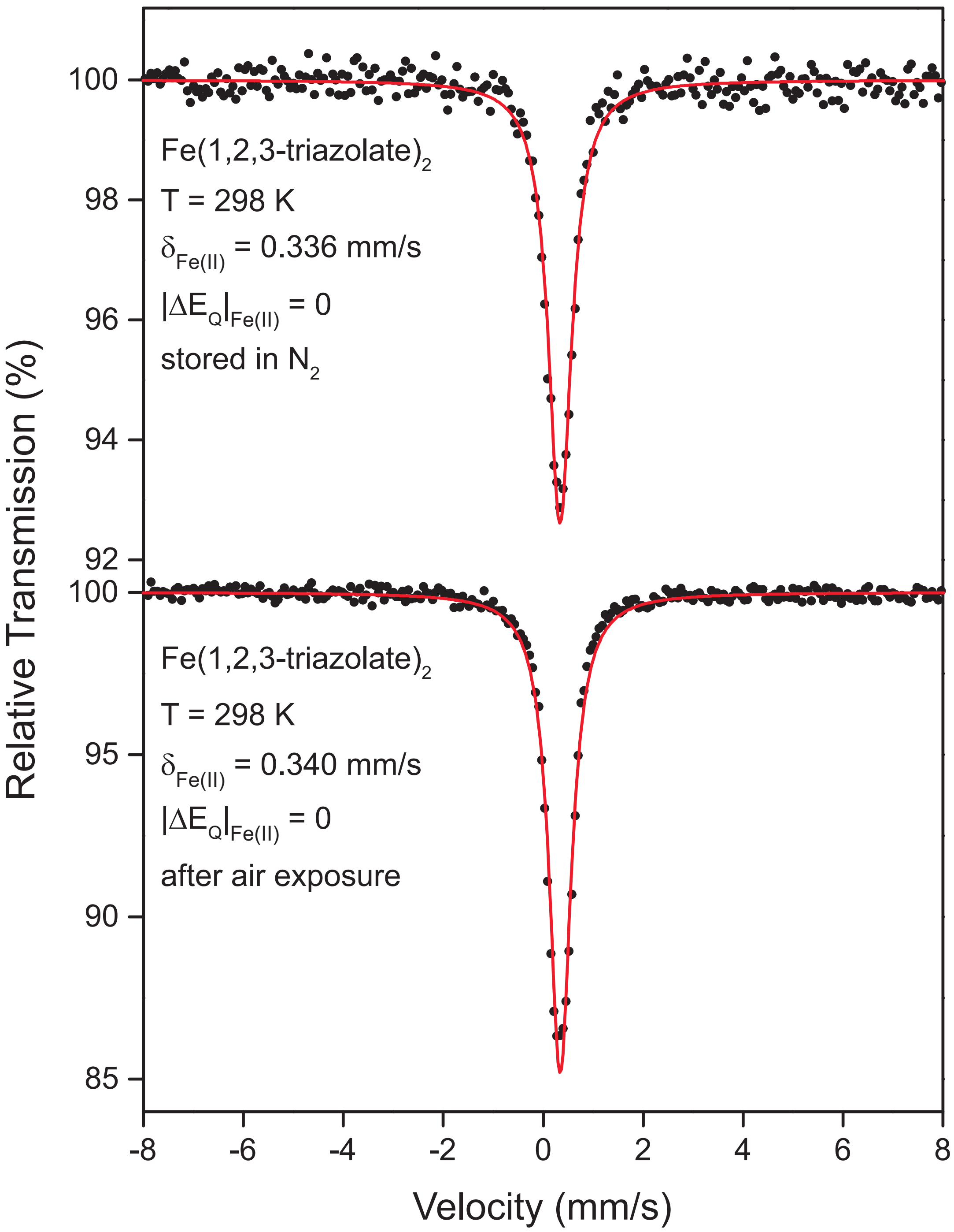

Figure S30. ^{57}Fe Mössbauer spectrum of Fe(1,2,3-triazolate)$_2$ that was exposed to air in comparison with the spectrum of Fe(1,2,3-triazolate)$_2$ that was stored in N_2 atmosphere. Fe^{2+} centers remained +2 oxidation state and low-spin state after air exposure. Black dots represent experimental data. Red curves represent fitting curves of Fe^{2+} sites. The isomer shift (δ) and the quadrupole splitting ($|\Delta E_Q|$) of the Fe^{2+} sites as well as the measurement temperature (T) are shown in the figure.

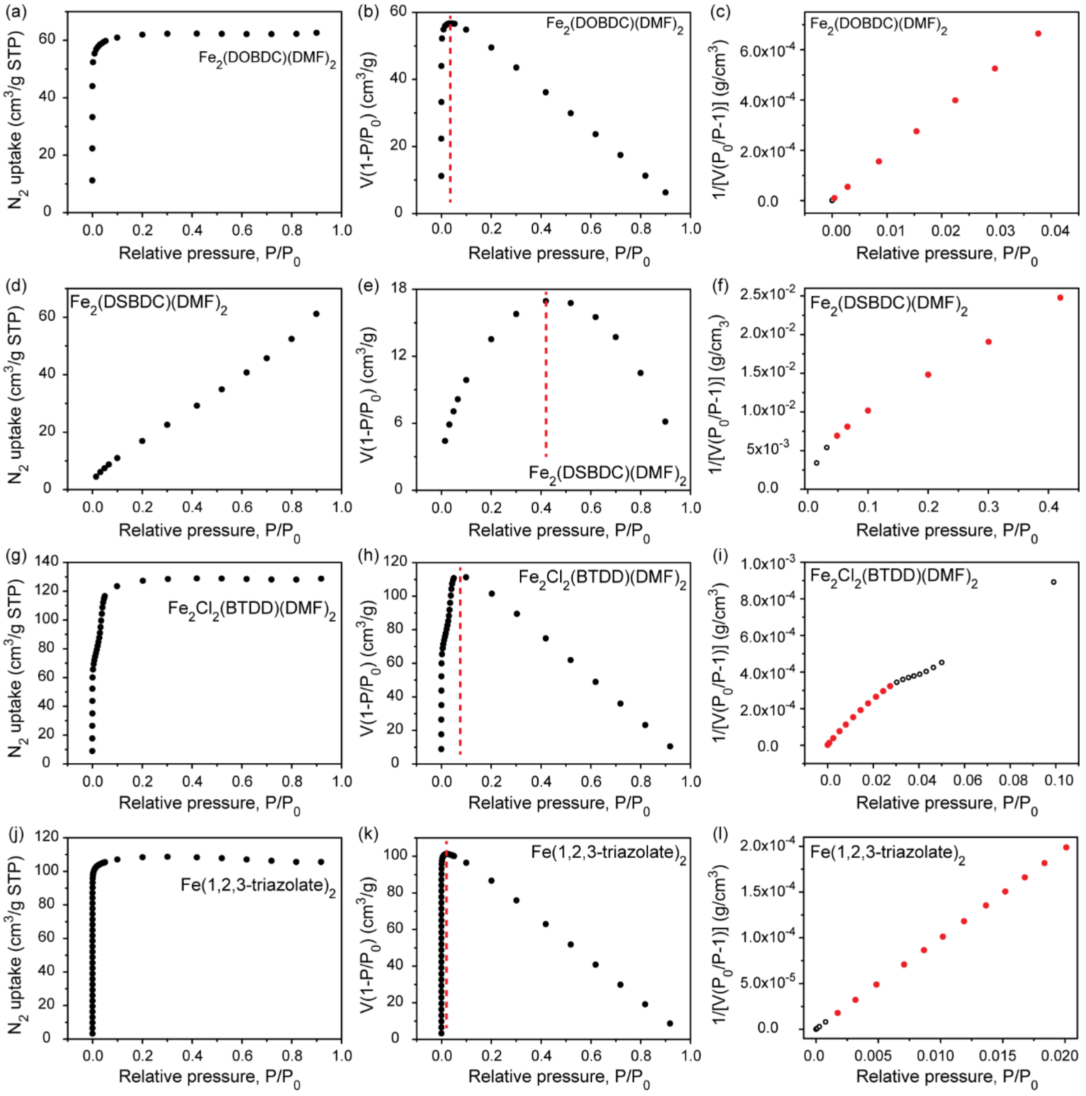

Figure S31. BET surface area analysis of Fe-based MOFs. (a)(d)(g)(j) N_2 adsorption isotherms collected at 77 K; (b)(e)(h)(k) $V(1–P/P_0)$ vs. P/P_0 plots used to determine the upper limit for BET linear fit, which is pointed out by the red dash line; (c)(f)(i)(l) BET linear fits. Red dots are linearly fitted to extract BET surface areas.

Table S3. Consistency criteria analysis of N2 sorption analysis.[a]

MOF	$Fe_2(DOBDC)(DMF)_2$	$Fe_2(DSBDC)(DMF)_2$	$Fe_2Cl_2(BTDD)(DMF)_2$	Fe(1,2,3-triazolate)$_2$
BET Surface Area	248 m^2/g	83 m^2/g	365 m^2/g	443 m^2/g
C	4101	10	1308	10109
Correlation coefficient	0.99998	0.998	0.995	0.999997
Fit range (P/P_0)	4.46×10^{-4} ~ 0.03766	0.04884 ~ 0.4196	7.942×10^{-5} ~ 0.02746	0.001741 ~ 0.02011
V_m	57.02 cm^3/g STP	19.14 cm^3/g STP	83.83 cm^3/g STP	101.68 cm^3/g STP
P/P_0 @ V_m	0.0154	0.237	0.0269	0.00985
$1/(\sqrt{C}+1)$	0.0154	0.237	0.0269	0.00985

[a] The consistency criteria analysis was performed based on a reported procedure.[21,22]

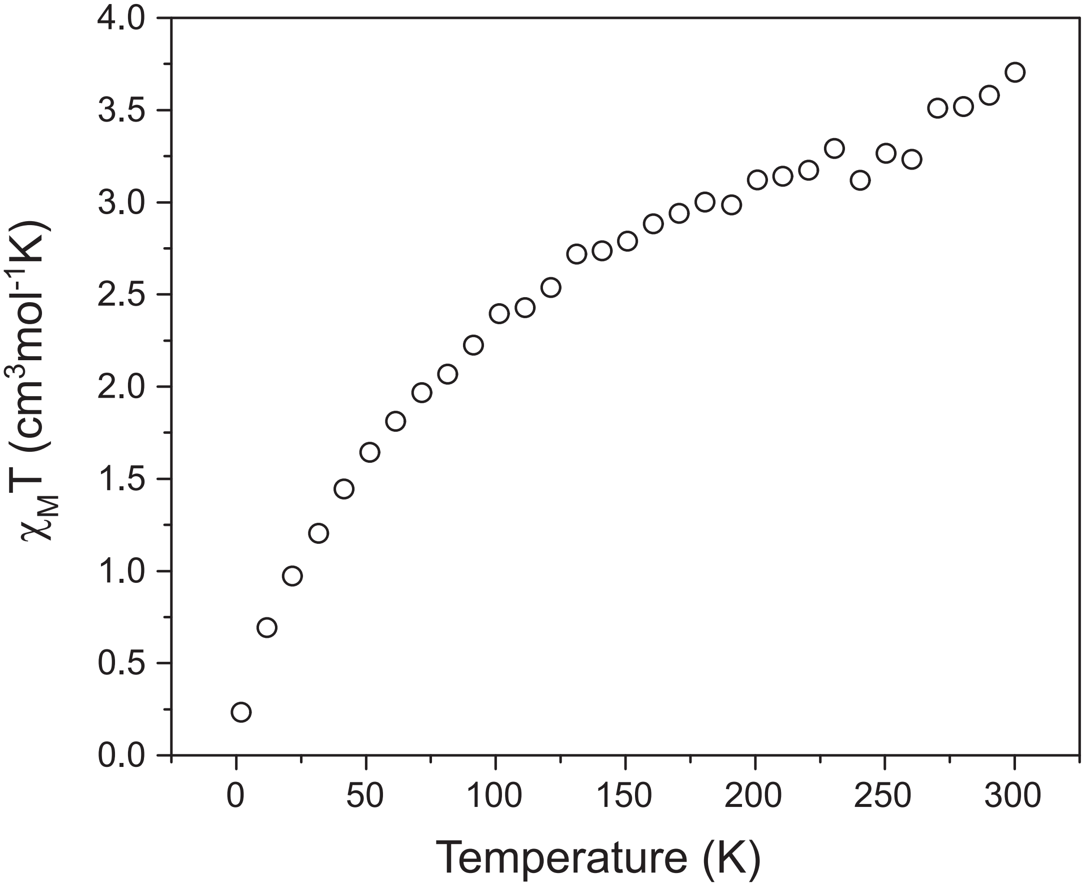

Figure S32. Variable-temperature magnetic susceptibility of Mn_2(DSBDC) in an applied field of 1 T. The room-temperature (300 K) effective magnetic moment, μ_{eff}, is 5.44 μ_B. This is slightly smaller than the value of 5.92 μ_B expected for high-spin Mn^{2+} with S = 5/2, possibly due to antiferromagnetic coupling between neighbouring Mn^{2+} sites and neighbouring $(-Mn-S-)_\infty$ chains. A similar phenomenon was observed in Mn_2(DOBDC).[4]

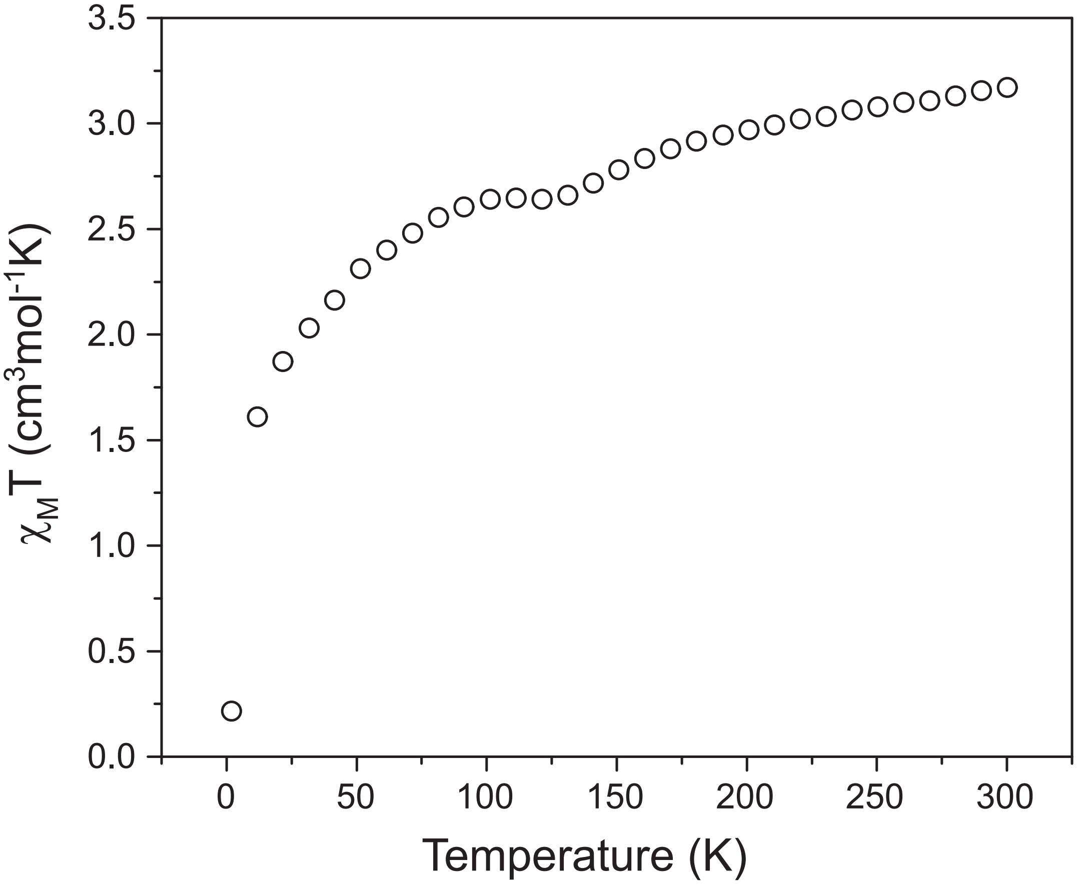

Figure S33. Variable-temperature magnetic susceptibility of Co_2(DOBDC) in an applied field of 1 T. The room-temperature (300 K) effective magnetic moment, μ_{eff}, is 5.03 μ_B. This is significantly higher than the spin-only value of 3.88 μ_B expected for high-spin Co^{2+} with S = 3/2, but falls in the range of 4.1-5.2 μ_B that is typically observed for high-spin octahedral Co^{2+}. The high μ_{eff} is attributed to the contribution of orbital angular momentum.[23]

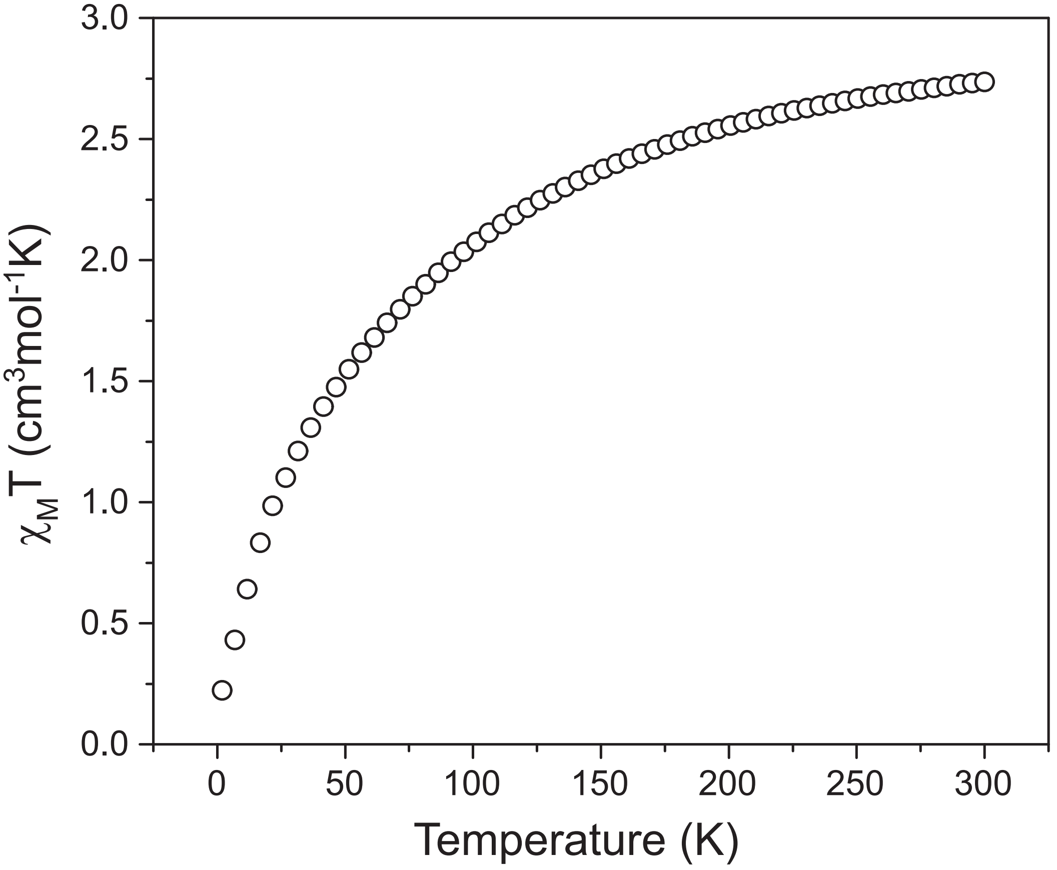

Figure S34. Variable-temperature magnetic susceptibility of Co(1,2,3-triazolate)$_2$ in an applied field of 1000 Oe. The room-temperature (300 K) magnetic moment, μ_{eff}, is 4.68 μ_B. This is significantly higher than the spin-only value of 3.88 μ_B expected for high-spin Co^{2+} with S = 3/2, but falls in the range of 4.1-5.2 μ_B that is typically observed for high-spin octahedral Co^{2+}. The high μ_{eff} is attributed to the contribution of orbital angular momentum.[23]

Table S4. Calculation results of M_2(DOBDC), M_2(DSBDC), and M(1,2,3-triazolate)$_2$.

MOF	E_{VBM} (eV)[a]	E_{CBM} (eV)[b]	E_g (eV)[c]	Experimental Ea (eV)[d]
Mg_2(DOBDC)	−5.9	−2.9	3.0	0.64
Mn_2(DOBDC)	−5.6	−3.2	2.4	0.55
Fe_2(DOBDC)	−5.2	−3.2	2.0	0.42
Ni_2(DOBDC)	−6.3	−3.3	3.0	0.62
Cu_2(DOBDC)	−6.1	−3.9	2.2	0.69
Zn_2(DOBDC)	−5.9	−2.7	3.2	0.54
Mn_2(DSBDC)	−6.2	−3.3	2.9	0.81
Fe_2(DSBDC)	−5.7	−3.8	1.9	0.27
Mg(1,2,3-triazolate)$_2$	−6.4	−0.9	5.5	0.74
Mn(1,2,3-triazolate)$_2$	−4.6	−1.5	3.1	0.59
Fe(1,2,3-triazolate)$_2$	−5.2	−0.8	4.4	0.39
Co(1,2,3-triazolate)$_2$	−5.3	−2.2	3.1	0.64
Cu(1,2,3-triazolate)$_2$	−5.3	−3.0	2.3	0.59
Zn(1,2,3-triazolate)$_2$	−6.7	−0.8	5.9	0.77
Cd(1,2,3-triazolate)$_2$	−6.8	−0.9	5.9	0.75

[a] Valence band maximum.

[b] Conduction band minimum.

[c] Band gap = conduction band minimum – valence band maximum.

[d] Experimentally observed activation energy.

Table S5. Properties of the interested 6-coordinated divalent metal ions.

	Mg^{2+}	Cr^{2+}	Mn^{2+}	Fe^{2+}	Co^{2+}	Ni^{2+}	Cu^{2+}	Zn^{2+}	Cd^{2+}
IE (eV)[a]	80.1437	30.96	33.668	30.652	33.50	35.19	36.841	39.723	37.48
E^0 (M^{3+}/M^{2+}) (V)[b]	N/A	-0.407	1.5415	0.771	1.92	N/A	2.4	N/A	N/A
E^0 (M^{2+}/M) (V)[b]	–2.70	–0.913	–1.85	–0.447	–0.28	–0.257	0.3419[c]	–0.7618	–0.4030
Z_{eff} [d]	4.521	3.599	3.735	3.482	3.564	3.687	3.675	3.879	4.260
$r_{crystal}$ (pm)[e]	86	94 HS<br>87 LS	97 HS<br>81 LS	92 HS<br>75 LS	88.5 HS<br>79 LS	83	87	88	109
$Z_{eff}/r_{crystal}$ (pm^{-1})[f]	0.053	0.038 HS<br>0.041 LS	0.039 HS<br>0.046 LS	0.038 HS<br>0.046 LS	0.040 HS<br>0.045 LS	0.044	0.042	0.044	0.049

[a] Ionization energies of divalent metal ions. These are equivalent to the 3rd ionization energies of the corresponding elements.[24]

[b] Standard reduction potentials of M^{3+}/M^{2+} pairs at 25 °C and a pressure of 101.325 kPa. The concentration of all ions is 1.000 mol/L.[25]

[c] E^0 (Cu^{2+}/Cu^{+}) = 0.153 V, E^0 (Cu^{+}/Cu) = 0.521 V.[25]

[d] Effective nuclear charge of divalent metal ions.[26] Data of Mg^{2+}, Cr^{2+}, Mn^{2+}, Fe^{2+}, Co^{2+}, Zn^{2+}, and Cd^{2+} are the reported Z_{eff}^{IE} in Ref. 26. Data of Ni^{2+} and Cu^{2+} are calculated based on the conclusion of Ref. 26 with ionization energies reported in Ref. 24.

[e] Crystal radii of 6-coordinated divalent metal ions.[27]

[f] Representative part of the Coulombic potential (E) of the valence electrons of divalent metal ions. $\mathrm{E} = -\frac{eZ_{eff}}{4\pi\varepsilon r_{crystal}}$, where e is elemental charge of an electron, and ε is dielectric constant in vacuum.

References

1 D.J. Sikkema, A. M. Reichwein, N. V. Akzo Nobel, U. S. Patent 09/071,782, May 1, 1998.

2 L. Vial, R. F. Ludlow, J. Leclaire, R. Pérez-Fernández and S. Otto, *J. Am. Chem. Soc.*, 2006, **128**, 10253–10257.

3 D. Denysenko, M. Grzywa, M. Tonigold, B. Streppel, I. Krkljus, M. Hirscher, E. Mugnaioli, U. Kolb, J. Hanss and D. Volkmer, *Chem. –Eur. J.*, 2011, **17**, 1837–1848.

4 A. F. Cozzolino, C. K. Brozek, R. D. Palmer, J. Yano, M. Li and M. Dincă, *J. Am. Chem. Soc.*, 2014, **136**, 3334–3337.

5 E. D. Bloch, L. J. Murray, W. L. Queen, S. Chavan, S. N. Maximoff, J. P. Bigi, R. Krishna, V. K. Peterson, F. Grandjean, G. J. Long, B. Smit, S. Bordiga, C. M. Brown and J. R. Long, *J. Am. Chem. Soc.*, 2011, **133**, 14814–14822.

6 L. Sun, T. Miyakai, S. Seki and M. Dincă, *J. Am. Chem. Soc.*, 2013, **135**, 8185–8188.

7 L. Sun, C. H. Hendon, M. A. Minier, A. Walsh and M. Dincă, *J. Am. Chem. Soc.*, 2015, **137**, 6164–6167.

8 F. Wudl and M. R. Bryce, *J. Chem. Educ.*, 1990, **67**, 717–718.

9 L. Sun, S. S. Park, D. Sheberla and M. Dincă, *J. Am. Chem. Soc.*, 2016, **138**, 14772–14782.

10 J. A. Mason, M. Veenstra and J. R. Long, *Chem. Sci.*, 2014, **5**, 32–51.

11 F. Gándara, F. J. Uribe-Romo, D. K. Britt, H. Furukawa, L. Lei, R. Cheng, X. Duan, M. O'Keeffe and O. M. Yaghi, *Chem. - A Eur. J.*, 2012, **18**, 10595–10601.

12 G. A. Bain and J. F. Berry, *J. Chem. Educ.*, 2008, **85**, 532–536.

13 K. T. Butler, C. H. Hendon and A. Walsh, *J. Am. Chem. Soc.*, 2014, **136**, 2703–2706.

14 P. D. C. Dietzel, R. Blom and H. Fjellvåg, *Eur. J. Inorg. Chem.*, 2008, 3624–3632.

15 P. D. C. Dietzel, Y. Morita, R. Blom and H. Fjellvåg, *Angew. Chem., Int. Ed.*, 2005, **44**, 6354–6358.

16 P. D. C. Dietzel, B. Panella, M. Hirscher, R. Blom and H. Fjellvåg, *Chem. Commun.*, 2006, 959–961.

17 W. L. Queen, M. R. Hudson, E. D. Bloch, J. A. Mason, M. I. Gonzalez, J. S. Lee, D. Gygi, J. D. Howe, K. Lee, T. A. Darwish, M. James, V. K. Peterson, S. J. Teat, B. Smit, J. B. Neaton, J. R.

Long and C. M. Brown, *Chem. Sci.*, 2014, **5**, 4569–4581.

18 N. L. Rosi, J. Kim, M. Eddaoudi, B. Chen, M. O'Keeffe and O. M. Yaghi, *J. Am. Chem. Soc.*, 2005, **127**, 1504–1518.

19 A. J. Rieth, Y. Tulchinsky and M. Dincă, *J. Am. Chem. Soc.*, 2016, **138**, 9401–9404.

20 X. Zhou, Y. Peng, X. Du, J. Zuo and X. You, *Cryst. Eng. Commun.*, 2009, **11**, 1964–1970.

21 J. Rouquerol, F. Rouquerol, P. Llewellyn, G. Maurin and K. S. W. Sing, *Adsorption by Powders and Porous Solids: Principles, Methodology and Applications*, Academic Press, London, United Kingdom, 2013.

22 T. C. Wang, W. Bury, D. A. Gómez-Gualdrón, N. A. Vermeulen, J. E. Mondloch, P. Deria, K. Zhang, P. Z. Moghadam, A. A. Sarjeant, R. Q. Snurr, J. F. Stoddart, J. T. Hupp and O. K. Farha, *J. Am. Chem. Soc.*, 2015, **137**, 3585–3591.

23 R. S. Drago, in *Physical Methods for Chemists*, Surfside Scientific Publishers, Gainesville, FL, 2nd edn., 1992, pp. 469–499.

24 In *CRC Handbook of Chemistry and Physics, Section 10 Atomic, Molecular, and Optical Physics*, CRC Press, Boca Raton, United States, 97th edn., 2016, pp. 10-197–10-199.

25 In *CRC Handbook of Chemistry and Physics, Section 5 Thermo, Electro & Solution Chemistry*, CRC Press, Boca Raton, United States, 97th edn., 2016, pp. 5-78–5-84.

26 A. Stokłosa, J. Zajęcki and S. S. Kurek, *Mater. Sci.*, 2004, **22**, 35–45.

27 R. D. Shannon, *Acta Crystallogr.*, 1976, **A32**, 751–767.
